# Osteopontin mediates acquired resistance to hypoxia-inducing antiangiogenics and promotes anti–PD-L1 refractoriness in breast cancer models

**DOI:** 10.1172/JCI174092

**Published:** 2026-07-15

**Authors:** Jose Luis Ruiz-Sepulveda, Maria J. Bueno, Silvana Mouron, Veronica Jimenez-Renard, Manuel Muñoz, Manuel Moradiellos, Leonardo D. Garma, Luis García-Jimeno, Adam W. Watson, Ghassan Mouneimne, Solip Park, Rebeca Jimeno, Miguel Quintela-Fandino

**Affiliations:** 1 Breast Cancer Clinical Research Unit and; 2 Computational Cancer Genomics Group, Centro Nacional de Investigaciones Oncológicas, Madrid, Spain.; 3 MeCo Diagnostics Holdings, Inc., San Diego, California, USA.; 4 University of Arizona Cancer Center, Tucson, Arizona, USA.; 5 Centro de Biología Molecular Severo Ochoa–CSIC, Universidad Autónoma de Madrid, Cantoblanco, Madrid, Spain.; 6 Endowed Chair of Personalized Precision Medicine, Universidad Autónoma de Madrid– Fundación Instituto Roche, Madrid, Spain.; 7 Medical Oncology Department, Hospital Universitario de La Princesa, Madrid, Spain.

**Keywords:** Immunology, Oncology, Breast cancer, Cancer immunotherapy, Hypoxia

## Abstract

Resistance to antiangiogenics is a major challenge in cancer therapy. These agents can either normalize or exacerbate tumor vascular abnormality and hypoxia. The mechanisms of resistance remain unclear in the latter setting. By integrating data from mouse models and clinical trials, we showed that hypoxia-inducing anti-VEGF therapy upregulated programmed cell death ligand 1 (PD-L1), yet failed to sensitize tumors to PD-L1 blockade. Mechanistically, early hypoxic stress triggered epithelial osteopontin (SPP1) production, which recruited monocytes and skewed macrophages toward M2 states, suppressing T cell cytotoxicity. Pharmacological SPP1 depletion impeded the development of hypoxia, reduced M2 infiltration, restored T cell activity, and enabled synergy between antiangiogenics and anti–PD-L1. Genetic dissection — tumor-epithelial *Spp1*-KO grafts and bone marrow chimeras generated by lethal irradiation and reconstitution with *Spp1*^–/–^ or WT hematopoietic donors — showed that myeloid SPP1 contributed only marginally compared with epithelial SPP1. These findings identified SPP1 as a central mediator of resistance to hypoxia-inducing antiangiogenics, contributed to a comprehensive model of antiangiogenic resistance, and supported SPP1-targeted strategies to personalize immunotherapy and antiangiogenic therapy according to tumor hypoxia.

## Abstract 2

This study reveals osteopontin’s key role in breast cancer’s resistance to antiangiogenic and anti-PD-L1 therapies, suggesting new treatment strategies.

## Introduction

Tumor vasculature is abnormal because of an imbalance between pro- and antiangiogenic signals, which sustains hypoxia and other protumorigenic features (1). Antiangiogenic therapy can transiently restore this balance and normalize the vasculature, thereby improving drug delivery and delaying progression (1). In breast cancer, this translated into consistent improvements in progression-free survival, but not in overall survival (OS), highlighting the lack of biomarkers able to identify the patients and treatment contexts most likely to benefit (2–5). We and others have shown that antiangiogenic exposure does not produce a uniform vascular response: tumors may either normalize or become more abnormal and hypoxic, even within the same histologic setting and after treatment with the same drug (6–9). This divergence, given the profound implications of hypoxia in tumor biology, is likely to condition subsequent adaptation to therapy.

We previously investigated resistance after vascular normalization and found in preclinical models that a shift from glycolysis to mitochondrial respiration sustained tumor growth in that setting (6); we then validated those findings in a prospective clinical trial (9). Here, we focused on the opposite trajectory, namely, tumors that respond to antiangiogenics by increasing vascular abnormality and hypoxia. Because hypoxia emerging after antiangiogenic treatment upregulates programmed cell death ligand 1 (PD-L1), we initially hypothesized that this branch might be therapeutically vulnerable to PD-L1 blockade (10, 11). However, our clinical and preclinical observations suggested that PD-L1 induction alone was insufficient to restore antitumor immunity; in fact, in a clinical trial, we observed that the efficacy of the combination of bevacizumab with the anti–PD-L1 agent durvalumab was only effective in patients with tumors with normalized vasculature (10). These observations raise the possibility that hypoxic tumors develop a broader immunosuppressive state that limits lymphocyte reinvigoration despite anti–PD-L1 therapy (10–15).

By studying murine breast tumors treated with antiangiogenics and focusing on the subgroup that develops increased hypoxia, we show that these tumors become refractory to PD-L1 blockade despite upregulating PD-L1. This phenotype is associated with expansion of a protumor myeloid infiltrate, particularly macrophage states linked to immune suppression. Mechanistically, we found that the initial hypoxic stimulus triggers tumor-epithelial secretion of SPP1, which recruits monocytes, promotes M2-like polarization, increases VEGF production, and establishes a feed-forward immunosuppressive loop involving both myeloid and lymphoid populations. Antibody-mediated SPP1 depletion disrupts this loop, restores antitumor immunity, and resensitizes tumors to PD-L1 blockade.

## Results

### Hypoxia-inducing antiangiogenic treatment upregulates the PD-1/PD-L1 axis, but blocking it in this context lacks efficacy both in animals and in patients.

**Figure 1:**
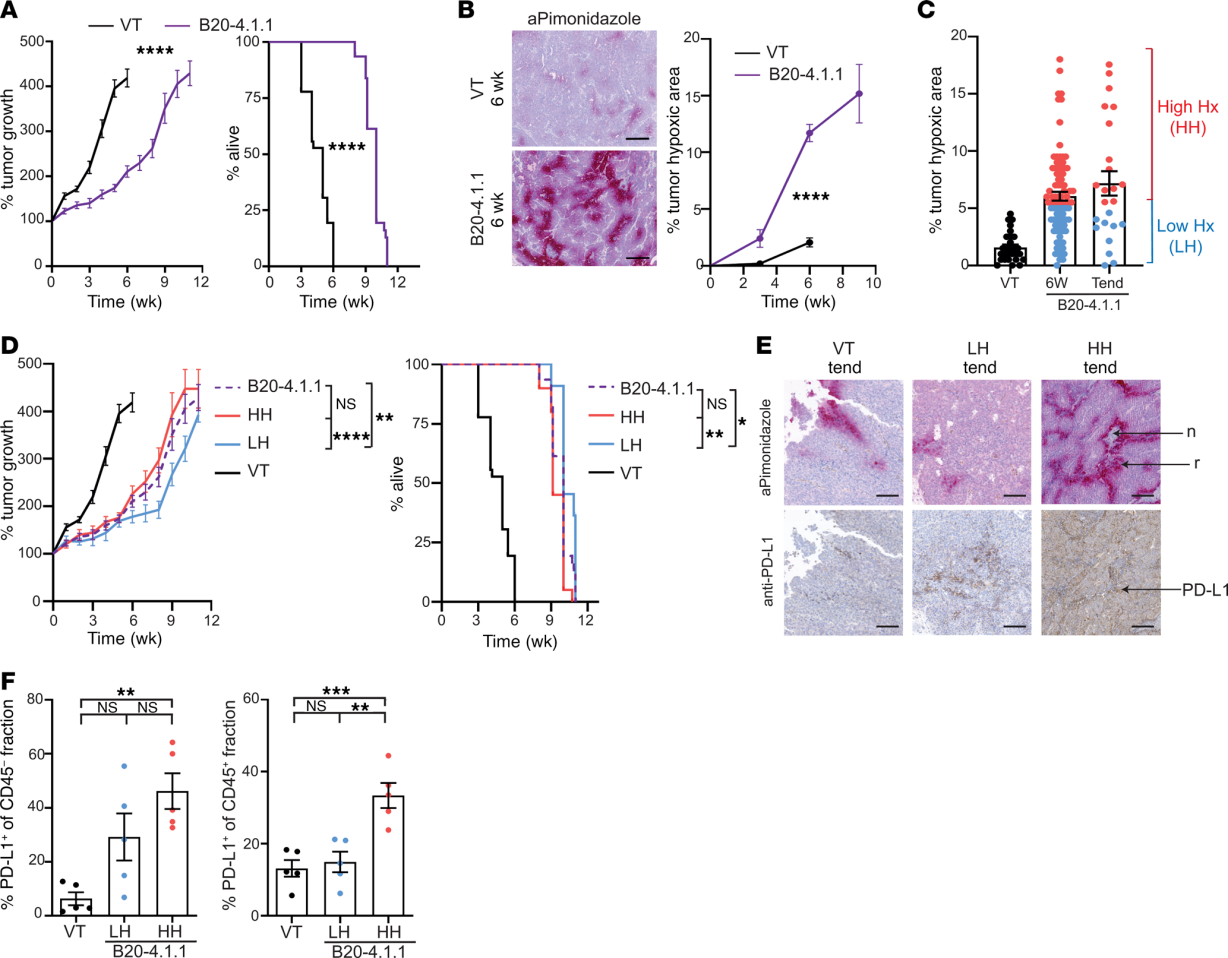
Anti-VEGF treatment generates hypoxic and nonhypoxic response patterns associated with differential PD-L1 induction. (**A**) Tumor growth and OS in response to B20-4.1.1 (*n* = 31) or isotype control (*n* = 36). Two-way ANOVA followed by Šidák’s multiple-comparison test. (**B**) Representative anti-pimonidazole (aPimonidazole) staining and quantification of tumor hypoxia over time in control- (*n* = 32) and B20-4.1.1–treated tumors (*n* = 90). Scale bars: 200 μm. Two-way ANOVA followed by Šidák’s multiple-comparison test. (**C**) Distribution of hypoxia values in individual tumors at the indicated time points, showing heterogeneous hypoxic responses to anti-VEGF treatment. Control (*n* = 28) or B20-4.1.1 at 6 weeks of treatment (*n* = 90), or Tend (*n* = 24). (**D**) Tumor growth and OS according to whether B20-4.1.1–treated tumors developed HH (*n* = 20 mice) or LH (*n* = 11 mice). Two-way ANOVA followed by Tukey’s multiple-comparison test. (**E**) Representative consecutive sections stained for pimonidazole and PD-L1, showing increased PD-L1 in hypoxic rims surrounding necrotic areas in HH tumors. Scale bars: 200 μm. (**F**) PD-L1 positivity in tumor cells and myeloid cells from vehicle-treated tumors (*n* = 5) and from LH or HH tumors after B20-4.1.1 (*n* = 5 each group). One-way ANOVA with Tukey’s post hoc test for multiple comparisons. Data are presented as mean ± SEM. *P < 0.05; **P < 0.01; ***P < 0.001; ****P < 0.0001. n, necrotic areas; r, rims.

We had previously observed that the anti-murine VEGF antibody B20-4.1.1, the murine analog of bevacizumab, was the antiangiogenic agent that most consistently increased vascular abnormality and tumor hypoxia while retaining antitumor activity (6). We therefore treated the FVB MMTV-PyMT breast cancer model with vehicle (isotype IgG2a control; VT) or B20-4.1.1. B20-4.1.1 significantly inhibited tumor growth and doubled median OS (5 vs. 10 weeks, *P* < 0.0001; Figure 1A) but also increased tumor hypoxia compared with VT (Figure 1B). However, the hypoxic response was heterogeneous across individual tumors in both groups (Figure 1C). To distinguish tumors that increased hypoxia after treatment from those that corrected it, we classified B20-4.1.1–treated tumors according to the median hypoxic fraction at week 6 (10.4% of tumor area), defining them as high hypoxia (HH) or low hypoxia (LH) tumors. This divergence had only a modest impact on tumor growth and survival within the treated cohort (median OS 9.1 and 10.0 weeks for HH and LH tumors, respectively; Figure 1D), indicating that the main consequence of this branching response was unlikely to be explained by direct differences in tumor growth control.

**Figure 2:**
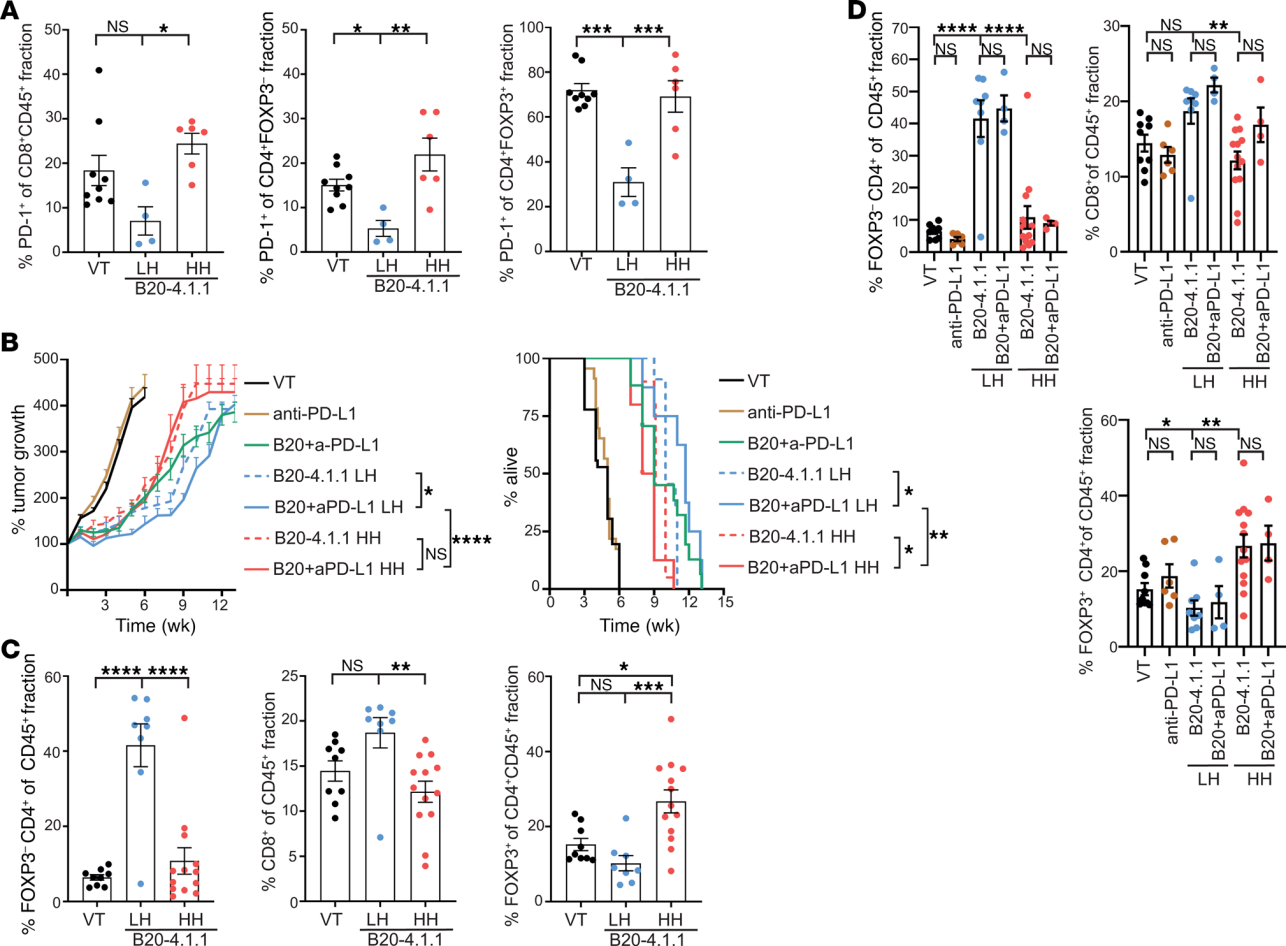
Hypoxic tumors remain refractory to PD-L1 blockade despite PD-1/PD-L1 upregulation. (**A**) PD-1 positivity in CD4^+^, CD8^+^, and Treg populations from vehicle-treated tumors and from LH or HH tumors after B20-4.1.1 (VT: *n* = 9; LH: *n* = 4; HH: *n* = 6). One-way ANOVA with Tukey’s post hoc test for multiple comparisons. (**B**) Tumor growth and OS after B20-4.1.1 plus anti–PD-L1, stratified according to LH (*n* = 7) and HH (*n* = 10) status. Log-rank (Mantel-Cox) test. (**C**) Percentage of CD4^+^, CD8^+^, and Treg lymphocytes among the CD45^+^ fraction of control (*n* = 9), HH (*n* = 13), or LH (*n* = 8) tumors. One-way ANOVA with Tukey’s post hoc test for multiple comparisons. (**D**) Lymphocyte composition in LH and HH tumors after addition of anti–PD-L1 (*n* = 4). One-way ANOVA with Tukey’s post hoc test for multiple comparisons. Data are presented as mean ± SEM. *P < 0.05; **P < 0.01; ***P < 0.001; ****P < 0.0001.

We next asked whether antiangiogenic-induced hypoxia altered the immune context of the tumors. Compared with VT and LH tumors, HH tumors showed higher PD-L1 expression, particularly along the gradient from normoxic regions to hypoxic rims and necrotic areas at the humane endpoint (Tend; Figure 1E). Flow cytometry confirmed increased PD-L1 protein levels in tumor cells (CD45^–^ fraction) and in the T cell–depleted CD45^+^ infiltrate (Figure 1F), together with increased PD-1 expression in CD8^+^, CD4^+^, and FOXP3^+^ Treg lymphocytes (Figure 2A).

Because hypoxia and inflammatory signals may both regulate PD-L1 (16), we explored these possibilities further. Although IFN-γ transcripts were increased in LH and not in HH tumors after B20-4.1.1 (Supplemental Figure 1A; supplemental material available online with this article; https://doi.org/10.1172/JCI174092DS1), direct in vitro hypoxia exposure of BRL1468 or RAW264.7 cells did not clearly upregulate PD-L1, whereas IFN-γ did so in both cell lines (Supplemental Figure 1, B and C). In addition, immunohistochemistry showed greater T cell accumulation in normoxic than in hypoxic tumor regions (Supplemental Figure 2), arguing against a simple model in which hypoxia alone is sufficient to produce effective immune reinvigoration.

To determine whether PD-L1 upregulation was functionally relevant for resistance, we combined B20-4.1.1 with anti–PD-L1. In the overall unstratified cohort, the combination did not significantly improve survival over B20-4.1.1 alone (Figure 2B; median OS for vehicle, anti–PD-L1, B20-4.1.1, and combination groups were 5, 5, 10, and 11 weeks, respectively). However, stratification according to hypoxic response revealed a marked divergence: in LH tumors, adding anti–PD-L1 increased median OS from 10.0 to 11.74 weeks, whereas in HH tumors, it reduced median OS from 9.14 to 8.5 weeks (Figure 2B).

We then asked whether this hypoxia-dependent modulation of antiangiogenic plus anti–PD-L1 efficacy could also be detected in patients. The JAVELIN 101 trial (17, 18) compared standard therapy with the antiangiogenic sunitinib against a combination of axitinib (antiangiogenic) and avelumab (anti–PD-L1) in kidney cancer. In the gene expression data of this study, using a hypoxia gene expression signature defined from baseline biopsies (Supplemental Figure 3A), patients classified as LH had longer median progression-free survival (PFS) than HH patients in the combination arm (13.1 vs. 11.1 months; Supplemental Figure 3B). In the IMbrave trial (antiangiogenic and sorafenib versus combination bevacizumab plus atezolizumab treatment in liver cancer) (19, 20), LH patients also showed numerically longer OS than HH patients, although without statistical significance (Supplemental Figure 3, C and D). Concordance between the hypoxia signature and pimonidazole-based classification of hypoxia is discussed in Supplemental Figure 3, E and F. In addition, in our own breast cancer trial combining bevacizumab with durvalumab, benefit from durvalumab was restricted to patients in whom bevacizumab had corrected hypoxia (10).

To further explore the role of antiangiogenic-induced hypoxia, we conducted a dose-response experiment with 0.5-fold, standard, and 2-fold doses of B20-4.1.1, seeking to increase tumor hypoxia by intensifying the pro/antiangiogenic factor imbalance (1). The highest dose produced the weakest antitumor effect, whereas the lowest dose produced the strongest one (Supplemental Figure 4A). Increasing dose also increased both the magnitude of hypoxia and interanimal variability (Supplemental Figure 4B), reduced CD4^+^ infiltration, increased Treg representation, and raised PD-1 levels in tumor-infiltrating lymphocytes (Supplemental Figure 4, C and D). Consistently, adding anti–PD-L1 improved OS only in the 0.5-fold group (Supplemental Figure 4E).

Together, these data indicate that the direction of the antiangiogenic response — toward hypoxia correction or toward hypoxia aggravation — shapes the immune infiltrate and determines the efficacy of subsequent anti–PD-L1 treatment. They also suggest that PD-L1 upregulation is not, by itself, sufficient to explain resistance in HH tumors, prompting us to analyze the immune infiltrate in greater depth.

### Tumors that develop hypoxia in response to antiangiogenics display myeloid inflammation features and are infiltrated by protumor macrophages.

Compared with LH tumors, HH tumors contained fewer CD4^+^ and CD8^+^ lymphocytes and more FOXP3^+^ Tregs (Figure 2C and Supplemental Figure 2A). Tumor hypoxia correlated inversely with CD4^+^/CD8^+^ infiltration and directly with Treg abundance (Supplemental Figure 5, A–C). Consistent with its lack of therapeutic efficacy in HH tumors, anti–PD-L1 did not significantly modify the lymphoid infiltrate (Figure 2D), nor did it reinvigorate CD4^+^, CD8^+^, or Treg populations according to PD-1 levels (Supplemental Figure 2B). Instead, in HH tumors, anti–PD-L1 was associated with an adverse cytokine profile, with a trend toward lower IFN-γ and higher TGF-β compared with B20-4.1.1 alone (Supplemental Figure 2C)

**Figure 3:**
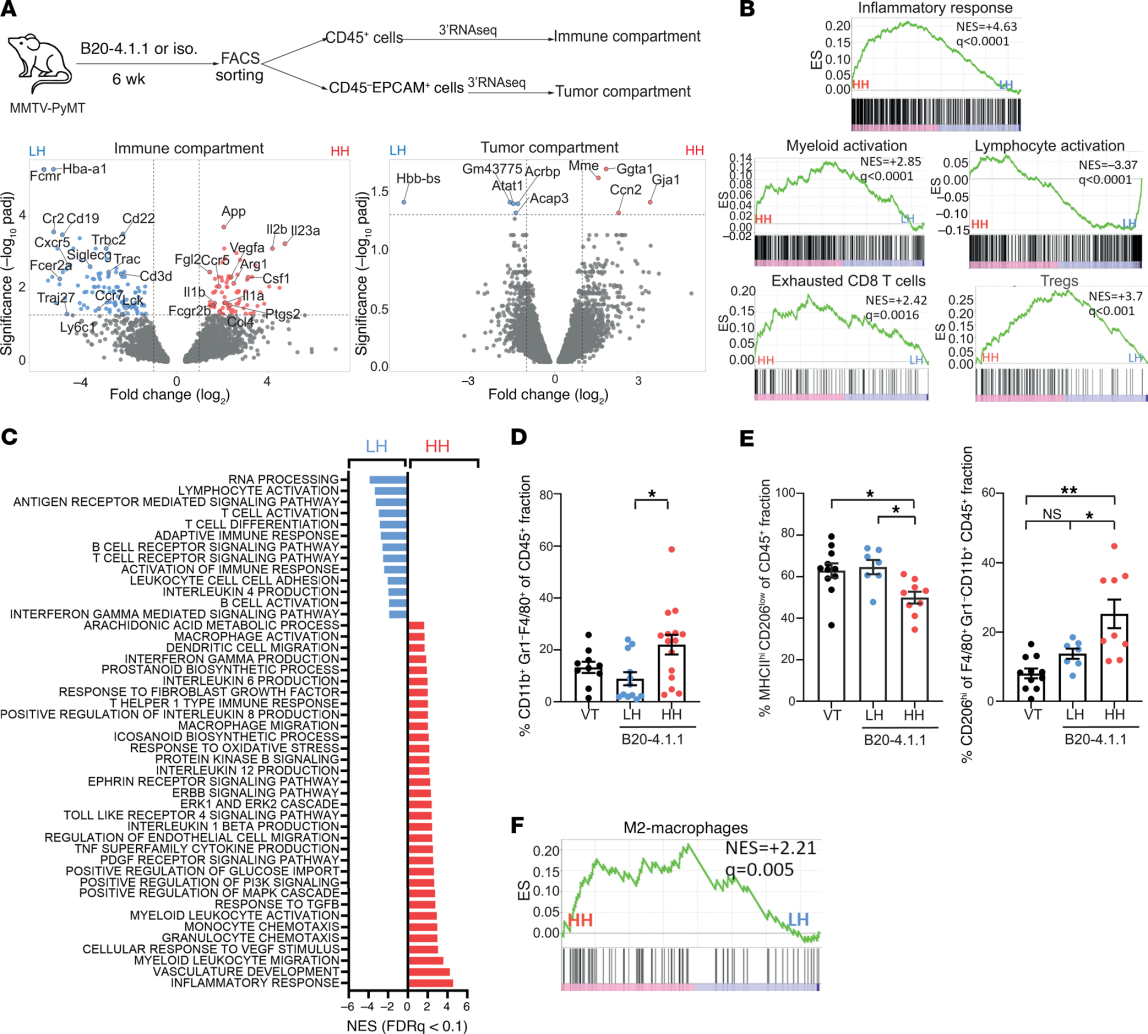
HH Tumors display a macrophage-rich, tumor-tolerant immune compartment. (**A**) Experimental scheme for bulk RNA-seq of sorted EPCAM^−^CD45^+^ immune cells and EPCAM^+^CD45^−^ tumor cells from LH and HH tumors and volcano plots of differentially expressed transcripts in each compartment. (**B**) Selected GSEA plots enriched in the CD45^+^ immune compartment of HH versus LH tumors. (**C**) Global pathway analysis of the CD45^+^ immune compartment comparing HH and LH tumors. (**D**) Percentage of macrophages within the CD45^+^ fraction in LH (*n* = 9) and HH (*n* = 10) tumors. One-way ANOVA with Tukey’s post hoc test for multiple comparisons. (**E**) Frequency of M1 and M2 macrophages in LH (*n* = 7) and HH (*n* = 9) tumors. One-way ANOVA with Tukey’s post hoc test for multiple comparisons. (**F**) M2-related GSEA in the immune compartment of HH versus LH tumors. Data are presented as mean ± SEM. *P < 0.05; **P < 0.01. ES, enrichment score; NES, normalized enrichment score.

To further characterize the functional differences between LH and HH tumors, we profiled gene expression in sorted epithelial and CD45^+^ tumor compartments (Figure 3A; Gene Expression Omnibus [GEO], GSE241539). Most transcriptional differences were found in the CD45^+^ fraction, whereas epithelial cells showed comparatively limited changes (Figure 3A and Supplemental Figure 6). In HH leukocytes, GSEA showed enrichment of inflammatory and myeloid activation programs together with reduced lymphocyte activation, increased CD8 exhaustion, and enhanced Treg activity (Figure 3, B and C). Flow cytometry confirmed a >2-fold increase in total macrophages in HH tumors (Figure 3D), which positively correlated with hypoxia (Supplemental Figure 7A). Using CD206 and MHCII (21) to define macrophage states, HH tumors were enriched in M2-like and depleted in M1-like macrophages relative to LH tumors (Figure 3E), and these populations correlated directly and inversely with hypoxia, respectively (Supplemental Figure 7B). Consistently, an M2 macrophage signature was enriched in the CD45^+^ compartment of HH tumors (Figure 3F).

### Macrophage depletion reverses HH-associated immune suppression, and HH-derived tumor-associated macrophages impair T cell effector function.

**Figure 4:**
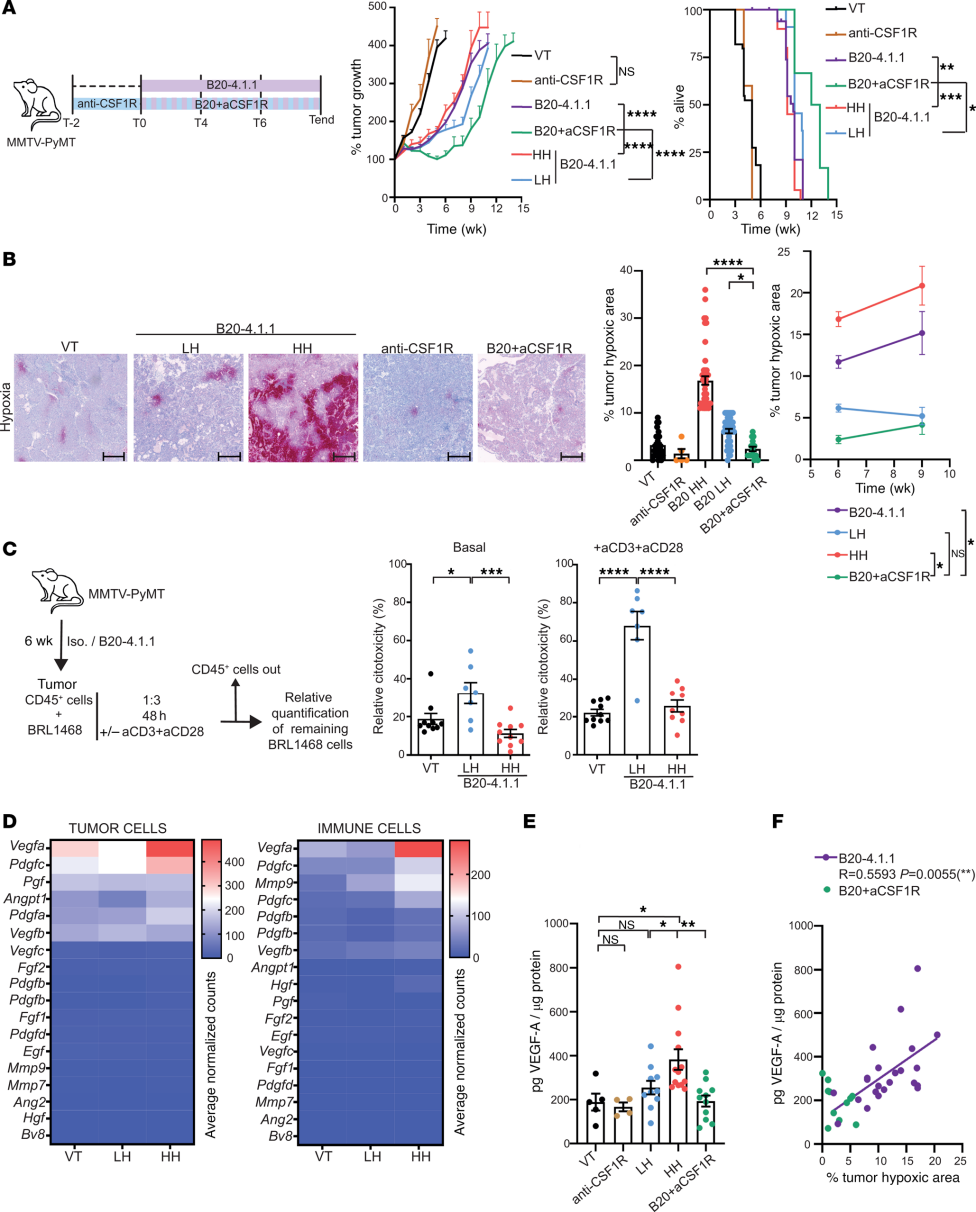
TAM depletion suppresses late hypoxia and angiogenic compensation and restores lymphocyte function. (**A**) Treatment scheme and effects of control (*n* = 36), anti-CSF1R (*n* = 5), B20-4.1.1 (*n* = 31), or the combination (*n* = 13) on tumor growth and OS. Two-way ANOVA followed by Tukey’s multiple-comparison test. (**B**) Representative hypoxia staining (scale bars: 200 μm) and quantification of hypoxia in tumors treated with vehicle (*n* = 28), LH (*n* = 40) and HH (*n* = 50) B20-4.1.1, anti-CSF1R (*n* = 5), and combination (*n* = 15), including late time points under continued therapy. One-way ANOVA with Tukey’s post hoc test for multiple comparisons. Right panel: average hypoxic levels of tumors treated with B20-4.1.1 anti-CSF1R or combination in final time points (B20: 6 wk *n* = 63, Tend *n* = 24; B20+aCSF1R: 6 wk *n* = 15, Tend *n* = 5). Two-way ANOVA followed by Šidák’s multiple-comparison test. (**C**) Cytotoxic activity of tumor-derived CD45^+^ cells from control (*n* = 10), LH (*n* = 7), and HH (*n* = 10) tumors against BRL1468 cells. One-way ANOVA with Tukey’s post hoc test for multiple comparisons. (**D**) Expression of selected angiogenesis-related transcripts in tumor and immune compartments across the indicated treatment groups. (**E**) VEGFA protein levels in tumors treated with vehicle (*n* = 5), LH (*n* = 10), HH (*n* = 13), anti-CSF1R (*n* = 4), and combination (*n* = 11). One-way ANOVA with Tukey’s post hoc test for multiple comparisons. (**F**) Correlation between VEGFA levels and tumor hypoxia in same tumors treated with B20-4.1.1 alone or in combination with anti-CSF1R. Data are presented as mean ± SEM. *P < 0.05; **P < 0.01; ***P < 0.001; ****P < 0.0001.

Given the known role of M2 macrophages in tumor progression and immunosuppression (22, 23), the enrichment of M2-like tumor-associated macrophages (TAMs) in HH tumors suggests that these cells might actively sustain the immunosuppressive phenotype in our model. We therefore depleted macrophages with an anti-CSF1R antibody before initiating antiangiogenic treatment (Figure 4A). Anti-CSF1R effectively reduced tumor macrophages (Supplemental Figure 8) and improved tumor control and OS (Figure 4A). Importantly, anti-CSF1R–treated tumors were no longer stratified into HH and LH because they failed to develop antiangiogenic-induced hypoxia under subsequent B20-4.1.1 treatment (Figure 4B). Compared with HH tumors, tumors treated with anti-CSF1R plus B20-4.1.1 showed increased CD4^+^ and CD8^+^ infiltration, reduced Tregs, lower PD-1 expression in lymphocyte subsets, and a more favorable cytokine profile, with lower immunosuppressive cytokines and increased IFN-γ (Supplemental Figure 9, A–C).

To determine whether the immune infiltrate of HH tumors was also functionally impaired, we performed an in vitro cytotoxicity assay using CD45^+^ cells isolated from tumors treated with B20-4.1.1 for 6 weeks (Figure 4C). Lymphocytes from HH tumors displayed reduced cytotoxic activity compared with those from LH tumors, both at baseline and after CD3/CD28 stimulation. We next asked whether TAMs from HH tumors were sufficient to impose this tolerant phenotype. TAMs isolated from vehicle-, LH-, or HH-derived tumors were cocultured with naive WT splenocytes according to the scheme shown in Supplemental Figure 9D. HH-derived TAMs promoted Treg differentiation and reduced IFN-γ production by both CD4^+^ and CD8^+^ cells (Supplemental Figure 9E). They also decreased the proliferative fraction of CD4^+^ and CD8^+^ lymphocytes, as measured by Ki67 (Supplemental Figure 9F). Consistently, supernatants from these cocultures showed increased CCL5 and TNF-α when macrophages were derived from HH tumors (Supplemental Figure 9G).

Together, these data indicate that TAMs are not merely associated with HH tumors, but functionally contribute to the tolerant immune microenvironment that emerges under hypoxia-inducing antiangiogenic treatment. The observation that macrophage depletion also prevents the subsequent development of hypoxia suggests that TAMs are part of a feed-forward loop rather than a terminal downstream consequence, prompting us to search for an earlier event upstream of macrophage recruitment.

### Osteopontin (SPP1) initiates macrophage recruitment and immunoregulatory polarization in response to antiangiogenic treatment.

A classical model of antiangiogenic resistance proposes that anti-VEGF treatment increases hypoxia, which in turn induces VEGFA and attracts monocytes/macrophages, thereby sustaining an angiogenic/inflammatory/necrotic cycle (24, 25). Our data are consistent with parts of this framework but also reveal an important paradox. On the one hand, HH tumors showed increased VEGFA transcription and protein levels in both epithelial and immune compartments at late treatment time points, and VEGF-related GSEAs were enriched, particularly in the immune compartment (Figure 4, D–F, and Supplemental Figure 10). On the other hand, although macrophage depletion decreased the amount of VEGF (Figure 4, E and F) — in accordance with the classic model — it also abolished the subsequent development of hypoxia despite continued antiangiogenic treatment (Figure 4, B and F), implying that macrophages are not merely a downstream consequence of hypoxia but part of a feed-forward loop required to sustain it. This raised 2 questions: what event precedes macrophage accumulation in HH tumors, and what promotes monocyte differentiation toward an M2-like state in this context?

**Figure 5:**
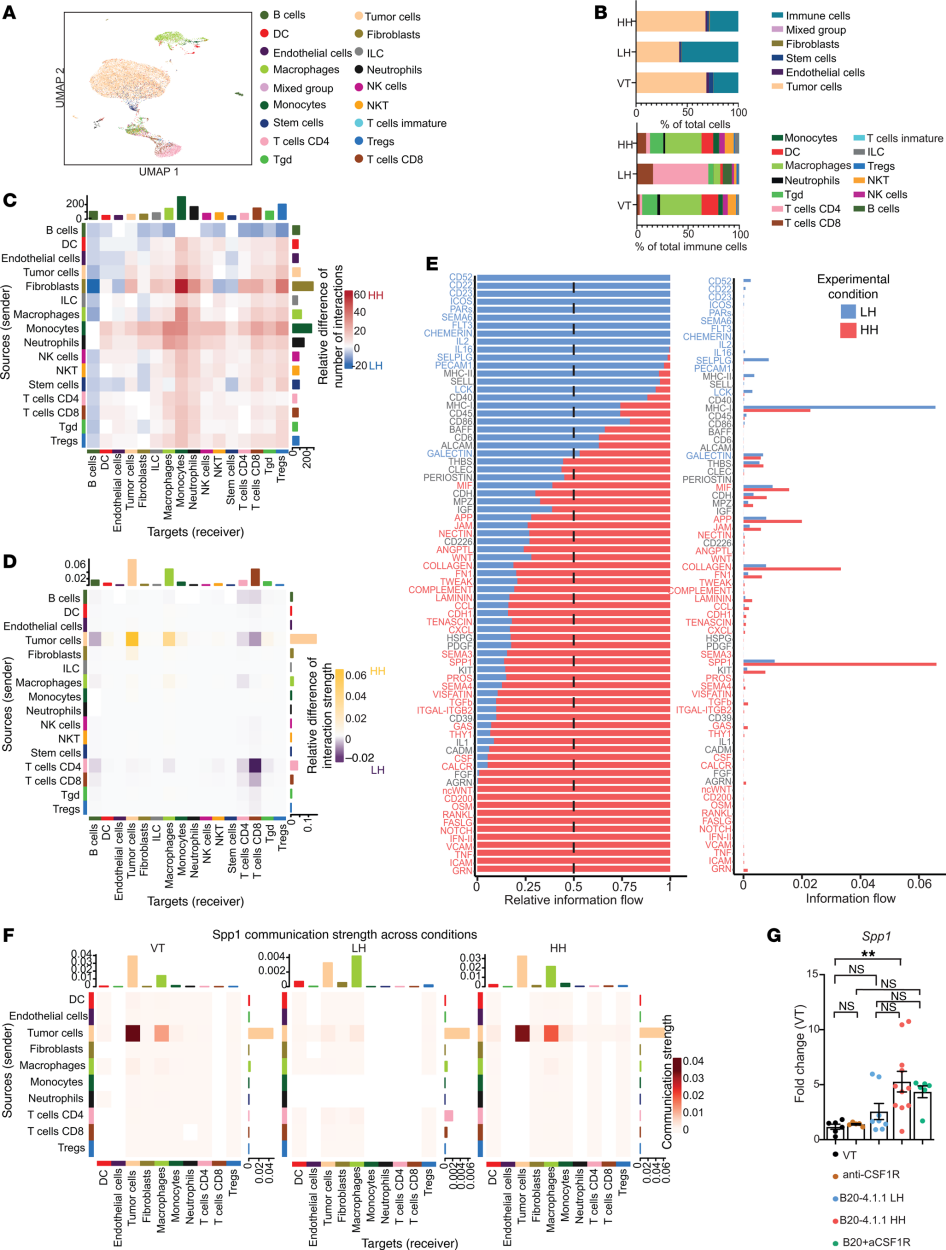
Single-cell analysis identifies SPP1-centered epithelial-to-myeloid communication in HH tumors. (**A**) UMAP representation of the major tumor and immune cell populations identified by scRNA-seq. (**B**) Relative distribution of nonimmune and immune cell populations across treatment groups. (**C**) Heatmap of the number of inferred cell-cell interactions in HH versus LH tumors. (**D**) Heatmap of interaction strength across cell populations in HH versus LH tumors. (**E**) Signaling flow analysis highlighting pathways enriched in HH or LH tumors. Blue or red coloring of pathways indicates a significant difference between HH and LH (Wilcoxon’s test, *P* < 0.05; gray, nonsignificant). (**F**) SPP1-mediated interaction strength across sender and receiver cell populations in vehicle-treated tumors and in LH or HH tumors after B20-4.1.1. (**G**) Transcriptional *Spp1* levels in tumors treated with VT (*n* = 6), anti-CSF1R (*n* = 5), LH (*n* = 10) or HH (*n* = 9) B20-4.1.1, and anti-CSF1R+B20-4.1.1 (*n* = 5). Experiment was performed in triplicate. One-way ANOVA with Tukey’s post hoc test for multiple comparisons. Data are presented as mean ± SEM. **P < 0.01.

To address these issues, we combined scRNA-seq with time-course analyses. We profiled 17,651 cells from 16 tumors (6 vehicle, 5 LH, and 5 HH). The pimonidazole-based hypoxia measurements correlated strongly with a hypoxia gene set score derived from the single-cell data, supporting the validity of our hypoxia classification method (Supplemental Figure 11A). UMAP visualization identified 18 major cell clusters (Figure 5A and Supplemental Figure 11B), and the overall lineage analysis revealed an inverse balance between tumor and immune cells in HH versus LH tumors, with higher macrophage abundance and lower CD4^+^/CD8^+^ representation in HH tumors (Figure 5B), in agreement with the flow cytometry results. The gene expression profiles presented in Supplemental Figure 11C highlight the up- and downregulated genes that distinguish each cluster.

We then subclustered the macrophage compartment and identified 6 TAM states (TAM0–TAM5; Supplemental Figure 12, A and B). Their gene expression profiles and pathway signatures were consistent with M1-like features in TAM0/TAM1, M2-like features in TAM3/TAM4, and more tissue-resident characteristics in TAM2/TAM5 (Supplemental Figure 12, A and C). HH tumors were enriched in TAM3 within a generally expanded macrophage compartment (Supplemental Figure 12D and Figure 5B). To specifically determine which TAM subsets expressed the highest *Spp1* levels, we profiled *Spp1* across TAM states and found TAM2 and TAM3 to be the *Spp1*-high subsets (Supplemental Figure 12E). Although these subsets are not proposed as the sole drivers of immune escape, they showed relative enrichment of immunoregulatory/checkpoint-associated genes (*Cd274*, *Pdcd1lg2*, *Vsir*, *Cd276*, *Lgals9*, and *Entpd1*) together with hypoxia/metabolic and microenvironment-shaping features (*Hif1a*, *Ldha*, *Vegfa*, *Tgfb1*, and *Lgals3*) (Supplemental Figure 12, F and G), supporting their role as myeloid amplifiers of the HH-associated immunosuppressive program. Together, these data point to a more immunosuppressed environment in HH tumors, dominated by different TAM variants with protumor and proangiogenic traits.

To determine how these cell populations interact, we performed CellChat analysis (26). HH tumors showed stronger epithelial→epithelial and epithelial→macrophage communication, whereas LH tumors retained stronger epithelial→CD8, CD4→CD8, and CD8→CD8 interactions (Figure 5, C and D). When we analyzed signaling flow across pathways, 3 major pathways stood out: collagen, MHCI, and SPP1, with *Spp1* showing the strongest differential increase in HH tumors (Figure 5E). Mapping the SPP1 pathway across sender and receiver populations confirmed that the dominant SPP1 communication in HH tumors occurred from epithelial cells to epithelial cells and, most prominently, from epithelial cells to macrophages (Figure 5F). These findings pointed to epithelial SPP1 secretion as a candidate initiating signal upstream of macrophage accumulation.

Because the earliest measurable consequence of anti-VEGF treatment is vascular pruning and perfusion loss (24, 25), we hypothesized that hypoxia itself might trigger epithelial SPP1 upregulation. An alternative hypothesis was considering compensatory VEGF secretion, which occurs in response to VEGF-clearing therapeutics (27), the initiating event. In multiple human breast cancer cell lines and in murine BRL1468 cells, hypoxia consistently induced *Spp1* mRNA, whereas VEGF alone did not (Supplemental Figure 13A). Conserved HIF1α-binding motifs were identified in both murine and human *Spp1* promoters (Supplemental Figure 13B), supporting a direct link between hypoxic stress and SPP1 induction.

**Figure 6:**
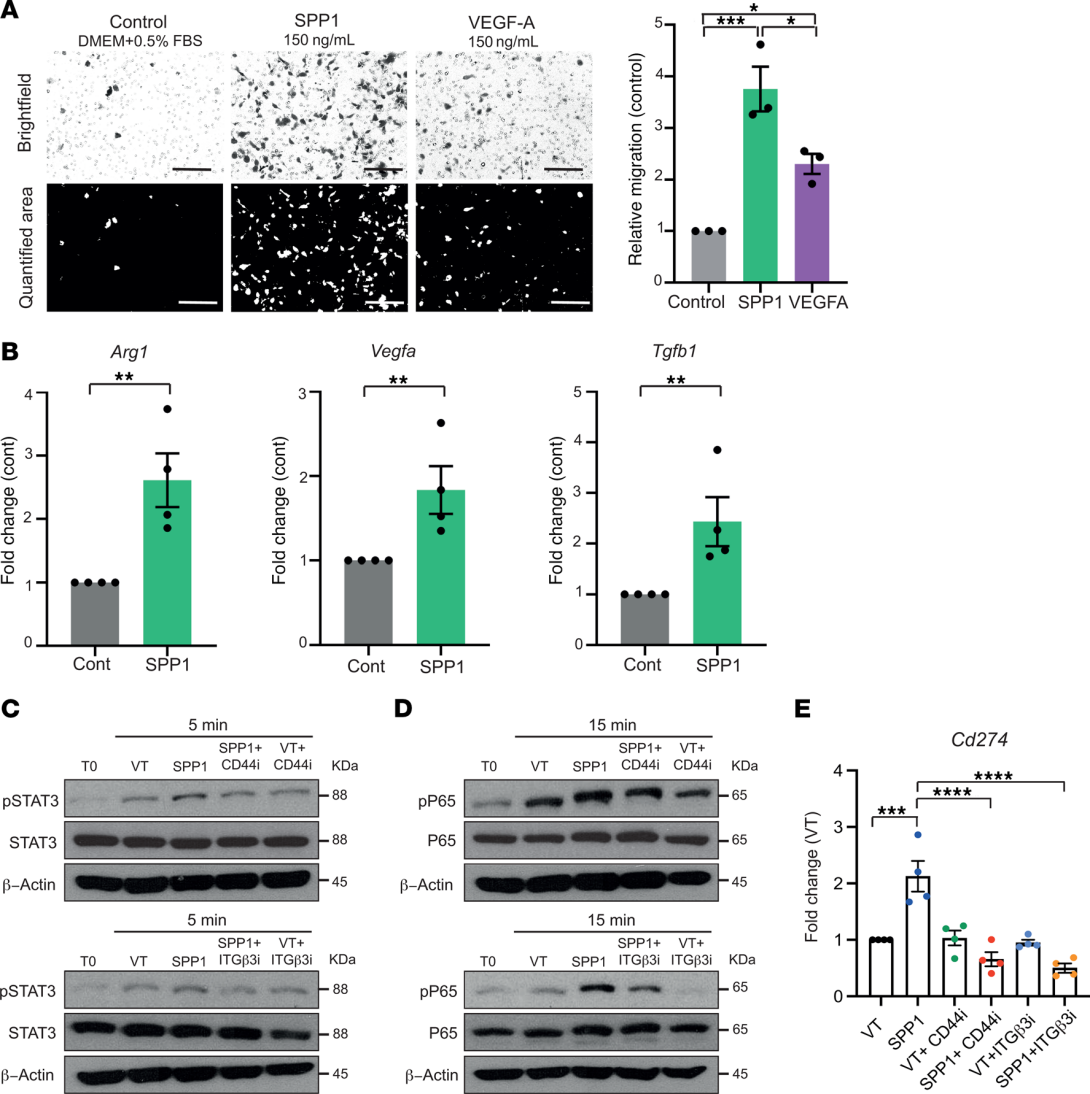
Extracellular osteopontin reprograms macrophages through CD44/integrin B3-linked signaling. (**A**) Transwell migration of macrophages in response to vehicle, recombinant SPP1, or VEGF-A. Scale bars: 200 μm. Right: quantitation chart. Experiment involved analyzing 10 images for each condition across 3 independent experiments (total 30 images per condition). One-way ANOVA with Tukey’s post hoc test for multiple comparisons. (**B**) Expression of M2-related transcripts in macrophages after exposure to recombinant SPP1 (*n* = 4). One-way ANOVA with Tukey’s post hoc test for multiple comparisons. (**C**) Representative images of immunoblots showing phosphorylation of STAT3 in response to stimulation (5 minutes) with SPP1 (5 μg/mL) or vehicle (VT) in RAW264.7 cells, in presence or absence of a CD44 inhibitor (anti-mouse/human CD44 IM7 antibody, 10 μg/mL; upper panel) or an ITGβ3 inhibitor (ITGB3-IN-1, 5 μM; lower panel) (*n* = 3). (**D**) Same as in **C** showing the effect of p65 phosphorylation in response to SPP1 (15 minutes) (*n* = 3). (**E**) PD-L1 regulation in RAW264.7 cells in the same conditions as **C** and **D** (*n* = 4). One-way ANOVA with Tukey’s post hoc test for multiple comparisons. Data are presented as mean ± SEM. *P < 0.05; **P < 0.01; ***P < 0.001; ****P < 0.0001.

We next explored the in vivo relevance of these findings. There are 5 known *Spp1* isoforms that have been involved in several tumor progression features (28–32); transcript-level analysis showed upregulation of multiple annotated *Spp1* splice variants in HH tumors (Supplemental Figure 14A). Importantly, these transcript-level data do not distinguish between extracellular/secreted and intracellular SPP1 protein pools. Secreted forms are pleiotropic (33) (OPNa and c are proangiogenic, and OPNa, b, and c attract monocytes), whereas intracellular isoforms can influence cell-intrinsic programs (34–36). Because the functional experiments below interrogate ligand-receptor communication, recombinant SPP1 activity, and antibody-mediated neutralization, the mechanistic conclusions of the present work primarily concern the extracellular SPP1 pool. HH tumors also displayed increased expression of integrin isoforms compatible with SPP1 signaling, including αVβ3 (proangiogenic) (37) and α9β1, which is a ligand of SPP1 and is implicated in wound-healing, angiogenesis, and cellular migration (38) (Supplemental Figure 14B). If extracellular SPP1 was hierarchically upstream of macrophage recruitment, one would expect that blocking macrophages would not eliminate SPP1 itself. Consistent with this, *Spp1* transcript levels were higher in HH than in LH tumors, and macrophage depletion — alone or combined with B20-4.1.1 — did not reduce *Spp1* levels (Figure 5G), supporting the interpretation that SPP1 elevation precedes macrophage accumulation. Recombinant SPP1 acted as a potent monocyte chemoattractant, stronger than VEGF in our assay (Figure 6A), and induced M2-associated transcriptional features in macrophages (Figure 6B). In addition, *Spp1* levels increased in parallel with antiangiogenic dose and hypoxia severity (Supplemental Figure 4B). To understand how extracellular SPP1 reprograms macrophages, we stimulated RAW264.7 cells with recombinant osteopontin under low-serum/BSA conditions and examined early signaling events downstream of its canonical receptors. SPP1 induced STAT3 phosphorylation after 5 minutes and NF-κB/p65 phosphorylation after 15 minutes, and both responses were attenuated by inhibition of CD44 or integrin β3 (Figure 6, C and D). We next asked whether this receptor-linked signaling was associated with an immunoregulatory output relevant to checkpoint refractoriness. Under the same low-serum conditions, recombinant SPP1 increased Cd274/PD-L1 expression in macrophages after 24 hours, and this induction was reduced by CD44 or integrin β3 inhibition (Figure 6E). Together with the migration data and the M2-related transcriptional changes induced by extracellular SPP1, these data delineate a minimal CD44/integrin β3–dependent signaling axis involving STAT3 and NF-κB/p65 activation, accompanied by PD-L1 induction, consistent with SPP1-driven immunoregulatory macrophage reprogramming. Thus, the former data support a revised model in which antiangiogenic-induced hypoxia first triggers epithelial SPP1 secretion; extracellular SPP1 then recruits monocytes, promotes immunoregulatory macrophage polarization, and amplifies a feed-forward loop involving VEGF, hypoxia, and immune suppression.

### Depleting extracellular SPP1 reverses HH-associated immunosuppression and restores sensitivity to PD-L1 blockade.

**Figure 7:**
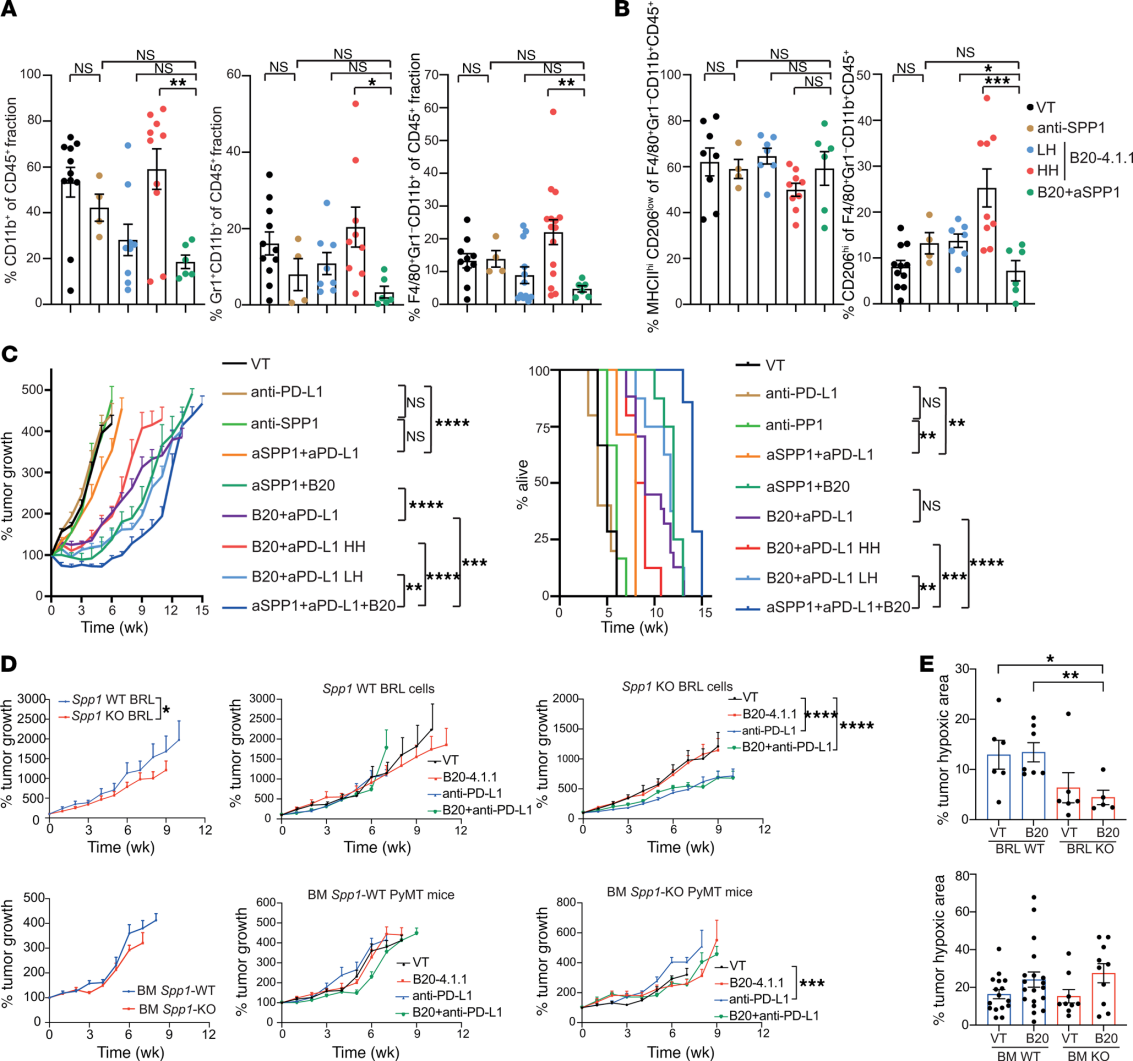
Systemic osteopontin blockade restores immune control, and tumor cells are the predominant source of SPP1. (**A**) Myeloid cells, macrophages, and myeloid-derived suppressor cells in tumors from the indicated treatment groups (VT: *n* = 11; anti-SPP1: *n* = 4; B20 LH: *n* = 9; B20 HH: *n* = 10). One-way ANOVA with Tukey’s post hoc test for multiple comparisons. (**B**) M1 and M2 macrophage fractions across the same treatment groups. (VT: *n* = 11; anti-SPP1: *n* = 4; B20 LH: *n* = 9; B20 HH: *n* = 10). One-way ANOVA with Tukey’s post hoc test for multiple comparisons. (**C**) Tumor growth and OS after anti–PD-L1, anti-SPP1, B20-4.1.1, or their combinations. Controls (*n* = 36); anti–PD-L1 (*n* = 23); anti-SPP1 (*n* = 5); anti-SPP1 + anti–PD-L1 (*n* = 6); HH (*n* = 20); LH (*n* = 10); B20-4.1.1+anti-SPP1 (*n* = 8); B20-4.1.1 + anti-SPP1 + anti–PD-L1 (*n* = 7). Two-way ANOVA followed by Tukey’s multiple-comparison test. (**D**) Phenotypic consequences of tumor cell versus myeloid SPP1 depletion, showing a predominant contribution of the tumor-epithelial compartment (*n* = 7–21 mice per group). Two-way ANOVA followed by Tukey’s multiple-comparison test. (**E**) Hypoxia development (in VT- or B20-4.1.1–treated tumors) is partially corrected in the epithelial KO model (*n* = 5–7) but not in the BM model (*n* = 9–19). One-way ANOVA with Tukey’s post hoc test for multiple comparisons. Data are presented as mean ± SEM. *P < 0.05; **P < 0.01; ***P < 0.001; ****P < 0.0001.

We next examined whether neutralizing extracellular SPP1 could reverse the immunosuppressive phenotype induced by hypoxia-promoting antiangiogenic treatment. Anti-SPP1 therapy prevented the development of treatment-induced hypoxia (Supplemental Figure 15A) and reverted the immune changes induced by B20-4.1.1, restoring CD8^+^ cells and reducing Tregs (Supplemental Figure 15B), while decreasing myeloid cells, macrophages, and myeloid-derived suppressor cells (Figure 7A). Importantly, M1 macrophages were preserved whereas M2 macrophages were reduced (Figure 7B), and PD-1 levels in lymphocyte subsets also decreased (Supplemental Figure 15C). Anti-SPP1 monotherapy had no antitumor effect, but its combination with B20-4.1.1 significantly prolonged median OS (Supplemental Figure 15D). Together, these data again support the interpretation that SPP1 lies upstream of macrophage recruitment and polarization in this setting.

We then asked how SPP1 contributes to resistance to anti–PD-L1 therapy. Transcriptomic analyses of the CD45^+^ compartment suggested impaired lymphocyte costimulation in HH tumors (Supplemental Figure 16A), and CellChat analysis of the CD86/CD28 and CD86/CTLA4 pathways supported weaker costimulatory interactions during antigen presentation in HH versus LH tumors (Supplemental Figure 16A). Expression of CD80, CD86, CD40, and CTLA4 was consistent with this pattern (Supplemental Figure 16B), providing a plausible explanation for the lack of benefit from PD-L1 blockade despite PD-1/PD-L1 upregulation.

To complement these in vivo findings, we exposed tumor-epithelial cells (BRL1468) and macrophages (RAW264.7) to recombinant SPP1 ex vivo. SPP1 was a stronger inducer of PD-L1 than hypoxia in both cell types (Supplemental Figure 17). In BRL1468 cells, SPP1 induced modest but coherent shifts in antigen presentation/costimulatory markers together with increased IL6 and TGF-β, consistent with a tolerogenic tilt (Supplemental Figure 18A). In RAW264.7 cells, SPP1 promoted an M2-skewed program (Figure 6B and Supplemental Figure 18B). Moreover, macrophages primed with SPP1 induced a broad immunosuppressive transcriptional program in cocultured naive splenocytes, despite only minor phenotypic changes by flow cytometry (Supplemental Figure 18, C–G). These findings support a mechanism whereby tumor-derived SPP1 conditions antigen presentation and costimulation, attracts and reprograms macrophages toward immunoregulatory states, and dampens T cell effector function.

Consistent with this model, SPP1 depletion restored costimulation-associated transcripts (Supplemental Figure 16B), recovered tumor lymphocyte cytotoxicity (Supplemental Figure 19), and, when added to B20-4.1.1 plus anti–PD-L1, induced tumor regression during the first 6 weeks and increased median survival by more than 50% compared with HH tumors (Figure 7C). These therapeutic results were reproduced in the 4T1 model (Supplemental Figure 20), and analysis of the JAVELIN 101 and IMbrave 150 cohorts showed higher *SPP1* levels in hypoxic tumors with poorer outcome to antiangiogenic plus anti–PD-L1 combinations (Supplemental Figure 21).

### Predominantly tumor-epithelial origin of SPP1.

Previous studies have highlighted immunosuppressive functions of myeloid-derived SPP1 (39–41). In contrast, in our antiangiogenic resistance model, the data indicate a predominantly tumor-epithelial source. To address this, we used 2 complementary genetic strategies: epithelial SPP1 deletion in BRL1468 tumor cells (BRL-KO) implanted into WT FVB recipients and bone marrow chimeras generated by reconstituting lethally irradiated FVB-MMTV-PyMT mice with WT or germline *Spp1*-KO marrow (Supplemental Figure 22A). BRL-KO tumors displayed markedly lower SPP1 levels than spontaneous PyMT tumors and tumors growing in BM-KO mice, supporting a predominant epithelial origin (Supplemental Figure 22B). B20-4.1.1 increased SPP1 in BRL-WT tumors but only modestly in BRL-KO grafts, consistent with residual epithelial production, whereas BM-KO tumors showed no reduction in SPP1 induction, arguing against a major myeloid source (Supplemental Figure 22B).

Functionally, epithelial SPP1 deletion modestly delayed tumor growth and sensitized tumors to anti–PD-L1, whereas myeloid deletion had minimal effects (Figure 7D). Although B20-4.1.1 showed limited activity in these graft and chimera settings, epithelial SPP1 loss reduced hypoxia under vehicle and antiangiogenic treatment, while myeloid deletion did not (Figure 7E). In parallel, BRL-KO tumors showed increased CD8 T cells, reduced Tregs, and lower macrophage, myeloid-derived suppressor cell, and M2 macrophage infiltration, whereas these changes were not evident in BM-KO tumors (Supplemental Figure 22, C and D). Together, these findings indicate that, in this context, SPP1 is predominantly epithelial in origin. Therapeutically, however, the strongest benefit is achieved by systemic anti-SPP1 antibody treatment, which neutralizes SPP1 regardless of its cellular source, whereas selective epithelial or myeloid deletions provide only modest benefit.

### Sequence of events: time course of SPP1, hypoxia, macrophages, and clinical adaptation to antiangiogenic monotherapy.

**Figure 8:**
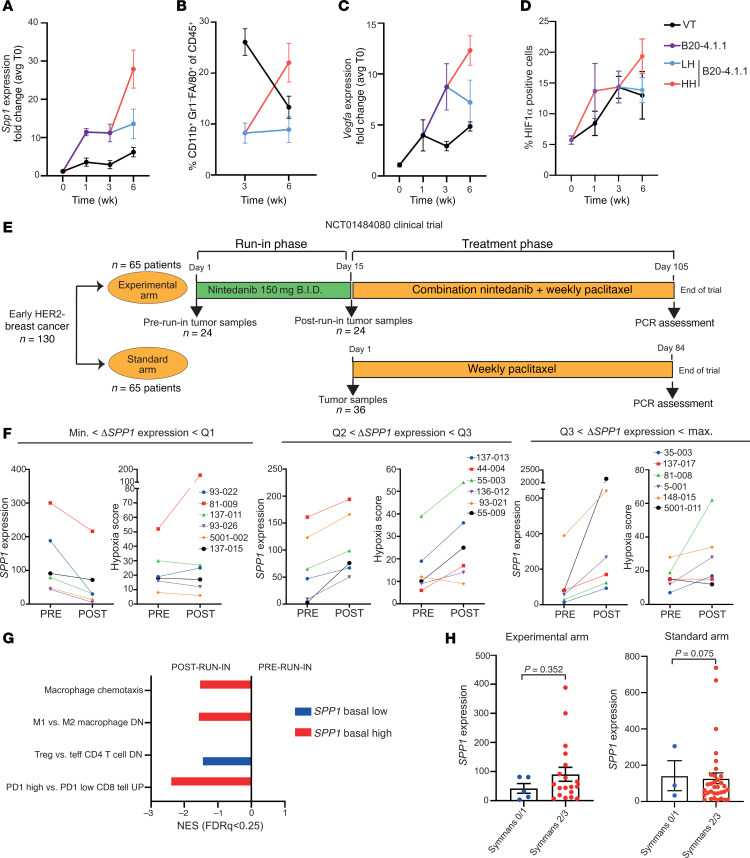
Time-course and clinical run-in analyses to support an osteopontin-centered adaptive program under antiangiogenic pressure. (**A**) Tumor SPP1 transcript levels during the first 6 weeks of anti-VEGF treatment (T0: *n* = 5; T1: VT *n* = 3, B20 *n* = 3; T3: VT *n* = 10, B20 *n* = 9; T6: VT *n* = 6, B20 LH *n* = 8, and B20 HH *n* = 11). (**B**) Macrophage abundance over the same time window (T3: VT *n* = 5, B20 *n* = 7; T6: VT *n* = 10, B20 LH *n* = 11 and B20 HH *n* = 15). (**C**) Transcriptional levels of VEGFA along the first 6 weeks of isotype or anti-VEGF treatment (T0: *n* = 5; T1: VT *n* = 3, B20 *n* = 3; T3: VT *n* = 10, B20 *n* = 9; T6: VT *n* = 3, B20 LH *n* = 4, and B20 HH *n* = 6). (**D**) Evolution of HIF1α staining during treatment (T0: *n* = 5; T1: VT *n* = 3, B20 *n* = 3; T3: VT *n* = 6, B20 *n* = 6; T6: VT *n* = 5, B20 LH *n* = 6, and B20 HH *n* = 6). (**E**) Design of the NCT01484080 clinical trial, including baseline and post–run-in sampling in the nintedanib-containing arm. (**F**) Individual changes in SPP1 expression and hypoxia score during the nintedanib run-in phase. (**G**) GSEA plots comparing pre– and post–run-in samples according to baseline *SPP1* levels. (**H**) Baseline *SPP1* levels according to pathological complete response category (Symmans 0/1 versus 2/3) in the nintedanib-containing arm (experimental arm) and in the paclitaxel-alone arm of clinical trial NCT01484080. Experimental arm: Symmans 0/1: *N* = 5; average *SPP1* = 42.3; Symmans 2/3: *N* = 19; average *SPP1*: 119.9; *P* = 0.35. Standard arm: Symmans 0/1: *N* = 3; average *SPP1* = 142.2; Symmans 2/3: *N* = 33; average *SPP1* = 127.9; *P* = 0.74).

To define the sequence of events linking antiangiogenic treatment to hypoxia and immunosuppression, we combined murine time-course studies with a clinical run-in analysis. In mice, hypoxia remained low during isotype treatment and increased only gradually under B20-4.1.1, reaching substantial levels at later time points (Figure 1B). By contrast, SPP1 was induced rapidly, already after 1 week of treatment (Figure 8A), whereas macrophage accumulation occurred later, mainly between weeks 4 and 6 (Figure 8B). VEGFA upregulation also appeared at later time points (Figure 8C). In parallel, HIF1α progressively accumulated after B20-4.1.1 treatment (Figure 8D), supporting a model in which early hypoxic stress triggers Spp1, followed by macrophage recruitment and later reinforcement of the hypoxic/VEGF state.

We next examined whether this sequence was recapitulated clinically in a randomized trial of early HER2^–^ breast cancer in which patients received a 2-week run-in of single-agent nintedanib before paclitaxel, compared with paclitaxel alone (ClinicalTrials.gov NCT01484080; Figure 8E). In the experimental arm, changes in SPP1 during the run-in phase were associated with concordant changes in hypoxia: patients in the lowest quartile of SPP1 change showed decreased hypoxia, whereas those in the highest quartiles showed increased hypoxia (Figure 8F). Baseline-high SPP1 tumors also developed a more immunosuppressive program during antiangiogenic exposure, with enrichment of M2-like macrophage, Treg, and PD-1^+^ CD8 signatures (Figure 8G). Finally, baseline SPP1 tended to be higher in tumors with less favorable pathological response in the nintedanib-containing arm, whereas this pattern was not observed in the paclitaxel-alone arm, although the study was not powered for formal treatment-interaction testing (Figure 8H).

**Figure 9:**
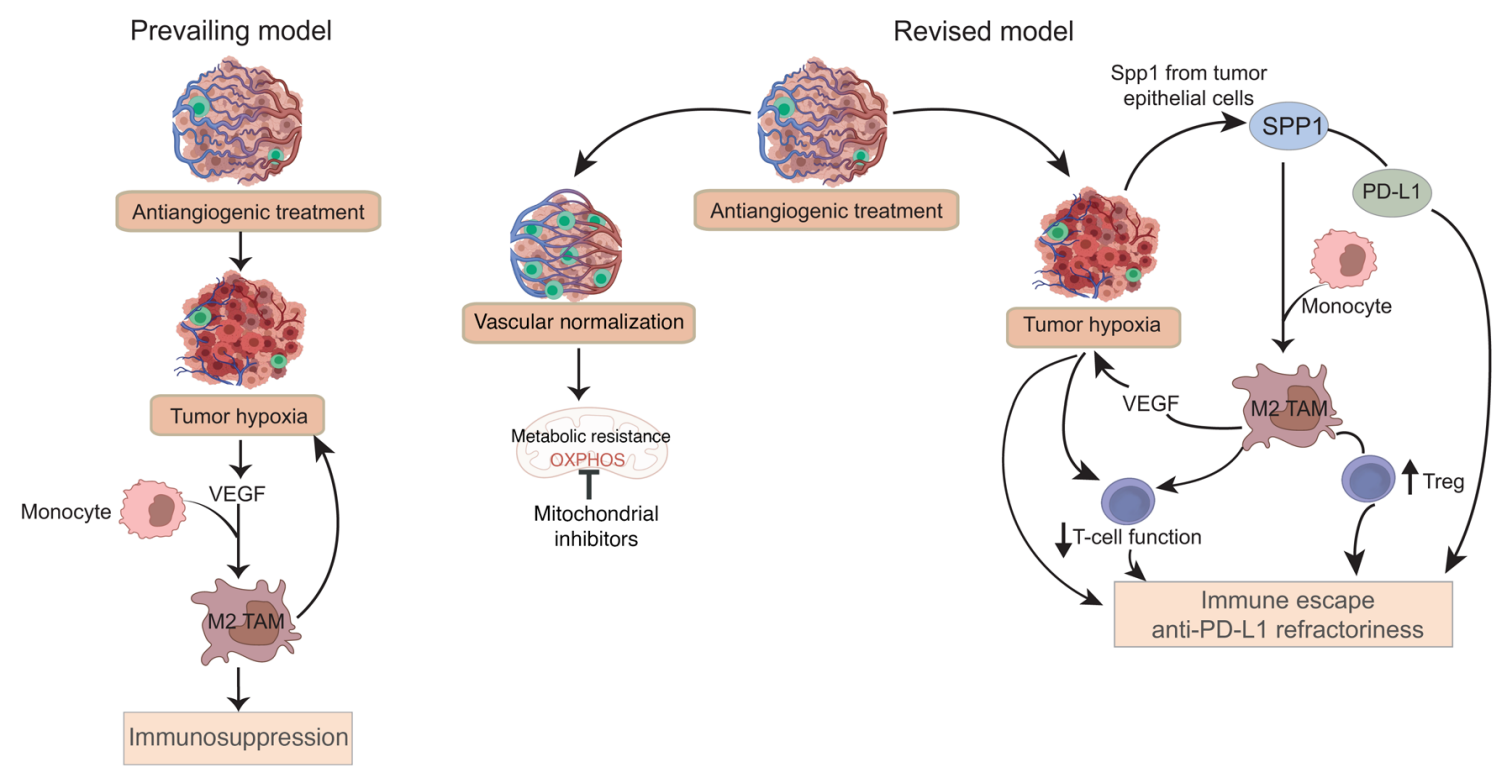
Proposed model of resistance to hypoxia-inducing antiangiogenics. Schematic representation of the proposed mechanism whereby antiangiogenic treatment that exacerbates hypoxia induces epithelial SPP1, promotes monocyte recruitment and macrophage immunoregulatory polarization, reinforces VEGF-driven hypoxic adaptation, and culminates in refractoriness to anti–PD-L1 therapy. The model also highlights the therapeutic points of intervention identified in this study, including CSF1R and Spp1 blockade.

Together, the murine and clinical data support a model in which antiangiogenic therapy that exacerbates hypoxia induces epithelial SPP1, which precedes macrophage accumulation and is followed by VEGF upregulation, deepening hypoxia, immune suppression, and refractoriness to PD-L1 blockade. This self-reinforcing hypoxia/SPP1/myeloid circuit is summarized in Figure 9.

## Discussion

Antiangiogenic therapy and PD-L1 blockade have shown only limited benefit in breast cancer, largely because biomarkers that identify responsive biological contexts are still lacking. Our data support a simple but important distinction: antiangiogenic exposure does not produce a uniform adaptive state. Instead, tumors may follow divergent trajectories, including a branch in which treatment aggravates hypoxia rather than correcting it. In our model, that branch was not associated with major differences in direct tumor growth control, but it was associated with marked changes in the immune microenvironment and with refractoriness to subsequent PD-L1 blockade (Figures 1–4). This observation may help explain why clinical activity of antiangiogenics and antiangiogenic/checkpoint combinations has often been modest when tested in unselected populations (10, 17, 19). The FVB/N background used here is relatively poorly immunogenic compared with strains such as C57BL/6, with a Th2-skewed immune tone and lower baseline immune surveillance (42–46). We therefore view it as a stringent context in which to test whether immune reprogramming can be restored once hypoxia-driven suppression is established. The fact that SPP1 blockade recovered cytotoxic T cell activity and responsiveness to PD-L1 inhibition even in this setting supports the biological relevance of the pathway. At the same time, our data do not suggest that this phenomenon is restricted to a single breast cancer subtype, because key findings were reproduced in both luminal B–like and triple-negative preclinical models, and translational analyses supported relevance in HER2^–^ disease. However, the present study was not designed to define subtype-specific effects in a definitive way, and larger dedicated cohorts are needed, particularly in HER2^+^ disease.

A central conclusion of our study is that SPP1 (osteopontin) is a major mediator of the hypoxia-associated adaptive state that emerges under antiangiogenic pressure. Elevated SPP1 has been linked to poor outcome in several tumor types (47), and myeloid-derived SPP1 has been implicated in CD8 suppression and macrophage recruitment in other settings (31, 41, 48). Our data suggest that, in the context of hypoxia-inducing antiangiogenic treatment, extracellular SPP1 contributes to immune escape at multiple levels. In tumor epithelium, it skews costimulatory signaling (CD86↑/CD80↔) and promotes a TGF-β/IL-6–rich milieu, limiting efficient T cell priming (Supplemental Figures 16 and 18); in myeloid cells, it promotes hypoxia-adapted, M2-like, and immunoregulatory TAM states that further suppress lymphocyte effector programs (Figure 5D and Supplemental Figure 12). This dual action explains why anti–PD-L1 alone is insufficient in HH tumors and why SPP1 blockade restores sensitivity by dismantling both epithelial and myeloid arms of the loop. Single-cell and CellChat analyses point to a dominant epithelium-to-myeloid communication axis centered on SPP1 (Figure 5), and functional assays show that recombinant SPP1 directly attracts macrophages and promotes M2-like transcriptional changes (Figure 6, A and B). In addition, extracellular SPP1 activates a CD44/integrin β3–linked STAT3 and NF-κB/p65 signaling axis, accompanied by PD-L1 induction in macrophages (Figure 6, C–E), supporting a mechanistic bridge between epithelial SPP1 secretion and macrophage immunoregulatory polarization. In vivo, SPP1 depletion reduced hypoxia, decreased M2 macrophages and other suppressive myeloid populations, restored T cell cytotoxicity, and resensitized tumors to anti–PD-L1 therapy (Figure 7, A–C, and Supplemental Figure 15). We therefore interpret SPP1 not merely as a biomarker of the HH state, but as an active component of the circuit that links hypoxia to macrophage-mediated immune dysfunction and checkpoint refractoriness.

These findings also refine prior VEGF-centered models of antiangiogenic escape. A classical framework proposes that antiangiogenic treatment increases hypoxia, which induces VEGF and attracts macrophages, thereby perpetuating hypoxia and immunosuppression (24, 25). Our results are compatible with parts of that model but suggest that an earlier event lies upstream of macrophage accumulation. First, macrophage depletion corrected hypoxia while leaving SPP1 levels largely unchanged, whereas SPP1 depletion prevented macrophage accumulation and blocked the subsequent hypoxic/immunosuppressive phenotype (Figures 4 and 7 and Supplemental Figures 15 and 16). Second, hypoxia, but not VEGF, robustly induced SPP1 in multiple epithelial cell lines, and conserved HIF1α-binding motifs were identified in the SPP1 promoter (Supplemental Figure 13). Third, time-course analyses showed that SPP1 rises early, before the late increase in macrophages and VEGF (Figure 8, A–D). Together, these data support a revised sequence in which an initial hypoxic insult caused by vascular pruning triggers epithelial SPP1, which then recruits monocytes, promotes immunoregulatory macrophage polarization, and amplifies a feed-forward loop involving VEGF, increasing hypoxia and immune suppression (Figure 9). Within the myeloid compartment, TAM2/TAM3 represent SPP1-high states enriched for hypoxia-adapted, proangiogenic, and immunoregulatory programs (Supplemental Figure 12), which likely act as amplifiers rather than initiators of the loop. This interpretation is also consistent with the distinction between extracellular and intracellular SPP1 pools: because our mechanistic experiments rely on ligand–receptor communication, recombinant SPP1 stimulation, and antibody-mediated neutralization, the conclusions of the present study primarily concern the extracellular/secreted pool. Secreted osteopontin retains the N-terminal signal peptide and acts extracellularly through receptors such as integrins and CD44, whereas intracellular osteopontin lacks the signal peptide and can influence cell-intrinsic programs (36). In cancer, secreted tumor cell–derived osteopontin can have both paracrine and autocrine effects, while intracellular/nuclear osteopontin has been linked to migration and epithelial–mesenchymal plasticity (34, 35, 49). Here, the core mechanistic experiments are based on ligand-receptor communication, recombinant SPP1 stimulation, and antibody-mediated neutralization (i.e., extracellular SPP1). We cannot exclude additional tumor-intrinsic contributions from intracellular osteopontin in our models, but these were not specifically dissected here.

The compartment-of-origin data are also informative. Previous literature has often emphasized myeloid SPP1 as an immunosuppressive mediator (31, 41, 48). In contrast, our genetic experiments indicate that, in this antiangiogenic resistance setting, SPP1 is predominantly epithelial in origin (Figure 7, D and E, and Supplemental Figure 22). Epithelial SPP1 deletion reduced SPP1 levels, hypoxia, and suppressive myeloid infiltration more clearly than bone marrow deletion, whereas myeloid SPP1 loss had relatively modest effects. At the same time, neither epithelial-only nor myeloid-only deletion reproduced the therapeutic effect of systemic anti-SPP1 antibody treatment, which suggests that both compartments can contribute functionally even if the dominant source is epithelial (Figure 7D). This distinction has mechanistic value, but from a therapeutic standpoint the more relevant point is that systemic neutralization of extracellular SPP1 appears more effective than compartment-restricted approaches. The same logic probably applies to the distinction between baseline hypoxia and antiangiogenic-induced hypoxia. Our data do not imply that both states are identical in all upstream determinants; rather, they suggest that they can converge on a common downstream SPP1-centered immunosuppressive program. In the clinical run-in cohort, baseline-high SPP1 tumors entered a more suppressive state under nintedanib exposure and showed a numerically adverse association with pathological response in the antiangiogenic arm but not in the paclitaxel-alone arm (Figure 8, E–H). Although this pattern is consistent with a context-dependent predictive effect, the current cohort was not powered for a formal treatment-by-biomarker interaction analysis, and larger controlled studies are needed to distinguish predictive from broader prognostic biology. Consistent with this interpretation, hypoxic tumors with poorer outcome in external antiangiogenic/anti–PD-L1 trials also showed higher SPP1 levels (Supplemental Figure 21).

From a translational perspective, these data suggest that hypoxia should be treated as a biologically meaningful stratification variable, not just as a descriptive correlative feature. Our previous work implicated mitochondrial inhibition as a rational partner for antiangiogenics that normalize the vasculature. Here, the branch that exacerbates hypoxia appears instead to be more sensitive to SPP1 and, potentially, CSF1R blockade. We therefore see these results not as a rejection of antiangiogenic therapy in breast cancer, but as support for a more selective use of the drug class according to the adaptive state induced in each tumor. A practical clinical strategy may involve early hypoxia assessment — potentially through noninvasive imaging (7, 8) or short antiangiogenic run-in designs — followed by mechanism-guided combination therapy. In that framework, SPP1 emerges as a druggable extracellular mediator for the hypoxia-driven branch. Antibody-mediated neutralization is currently the most direct validated strategy, whereas additional approaches such as ligand–receptor blockade or aptamer-based modalities remain preclinical (50–52). Overall, our findings support a model in which a subset of tumors exposed to antiangiogenic therapy enter a self-reinforcing hypoxia/SPP1/myeloid circuit that culminates in immune escape and refractoriness to PD-L1 inhibition (Figure 9). Interfering with that circuit restores immune competence and may widen the therapeutic window of antiangiogenic therapy in breast cancer.

## Methods

### Sex as a biological variable.

This study was conducted using female mice. Females were selected due to the relevance of breast cancer to female biology. While the findings provide insights into disease mechanisms, further studies are needed to determine their applicability to males.

### Mouse models.

MMTV-PyMT mice [FVB/N-Tg (MMTV-PyVT)^634Mul/J^]and BALB/c were bred at the Centro Nacional de Investigaciones Oncológicas (CNIO) Animal Facility. Mouse strains were maintained under specific pathogen–free conditions at the CNIO Animal Facility. Animals were kept in a climate-controlled environment (22 ± 2°C) with 12-hour light/12-hour dark cycles and with ad libitum access to food and water.

For the orthotopic 4T1 mouse model, 2 × 10^5^ 4T1 cells were resuspended in 50% Matrigel (Corning) and injected (50 μL volume) in the mammary fat pad of WT BALB/c mice. Methods for tumor-epithelial SPP1 KOs and bone marrow *Spp1*-KO chimera generation are described in Supplemental Methods.

### Animal treatments and tumor measurements.

Treatment allocations were randomly assigned using a computer-generated random number (www.randomizer.org). Treatments with the different drugs were started at 7 weeks of age of MMTV-PyMT mouse models and at tumor size of 200 mm^3^ in the 4T1 orthotopic mouse model. Anti-VEGFA (clone B20-4.1.1, acquired with a Material Transfer Agreement with Genentech) or isotype mouse IgG2a (clone C1.18.4, Bio X Cell, BE0085) and anti–PD-L1 (clone 10F.9G2, Bio X Cell, BE101) or isotype rat IgG2b (clone LTF-2, Bio X Cell, BE0090) were prepared in 1× PBS and administered at 5 mg/kg intraperitoneally twice per week. Anti-SPP1 (clone 103D6, Bio X Cell, BE0373) or isotype mouse IgG2c (clone DV5-1, Bio X Cell, BE0366) was prepared in 1× PBS and administered at 10 mg/kg intraperitoneally twice per week. Anti-CSF1R (clone AFS98, Bio X Cell, BE0213) or isotype rat IgG2a (Bio X Cell, BE0089) was prepared in 1× PBS and administered at 20 mg/kg intraperitoneally 3 times per week. Treatment combinations were administered as indicated for monotherapy regimes. Pimonidazole-HCl (Hypoxyprobe Kit, HP1-1000Kit) for hypoxia detection was prepared in saline and administered at 60 mg/kg intraperitoneally 1 hour before euthanasia. Tumor dimensions were measured once per week using digital calipers. Tumor volumes were calculated using the formula V = (D × d^2^)/2 mm^3^, where D is the largest diameter and d the shortest diameter; all measurements were in millimeters. All tumors arising in mammary glands were measured in each animal. Mice were euthanized in a CO_2_ chamber at the time point required or, at maximum, when reaching humane endpoint (tumor volume ≥ 1,200 mm^3^). In MMTV-PYMT mice, only upper mammary gland tumors were dissected and used for experimental procedures. Depending on the procedure, tumors were fixed in 10% solution (Sigma-Aldrich, HT501128) and embedded in paraffin, snap-frozen in isopentane, or OCT embedded for cryopreservation (TissueTek, Sakura Finetek, 4583).

### Tumor cell suspension preparation.

Tumors were harvested, minced into approximately 2 mm^3^ pieces, placed in DMEM medium with Mouse Tumor Dissociation Kit enzymes (Miltenyi Biotec, 130-096-730), and incubated in gentleMACS C tubes (Miltenyi Biotec, 130-093-237) on a gentleMACS Octo Dissociator (Miltenyi Biotec, 130-095-937) using the 37C_m_TDK_2 program. Then, 10 mL of 10% FBS DMEM medium was added to stop enzyme reaction, and cell suspension was passed through a 40 μm strainer. Next, centrifugation was performed at 300*g* for 5 minutes at 4°C, and cell pellet was resuspended in RBC 1× lysis buffer (eBioscience, 00-4333-57). After washing with 1× PBS, cell suspension was resuspended in corresponding buffer and used for the following experiments.

### Migration assays.

For in vitro macrophage migration assays, 50,000 RAW264.7 cells purchased from American Type Culture Collection were cultured in the upper chamber of a 96-well permeable support plate (pore size: 8 μm) (Corning Life Science, 3464) in 0.5% FBS DMEM medium (Sigma-Aldrich, D5796). The lower chamber contained medium supplemented with or without the tested chemoattractants, VEGFA (150 ng/mL; Preprotech, 450-32), or SPP1 (150 ng/mL; Biotechne, 441-OP-050/CF), and the plate was incubated for 18 hours. After incubation, the nonmigrated cells were removed from the upper chamber. The migrated cells that adhered to the underside of the inserts were fixed with 4% PFA for 30 minutes and stained with 0.2% Crystal violet for microscopy visualization. For cell migration quantification, 10 random fields of each condition were digitalized at ×200. The migration capacity was measured by quantifying the percentage of the area occupied by stained cells using ImageJ (NIH). Relative migration induced by each treatment was measured by relativizing the percent cell area in treated transwells to the control ones.

### In vitro cytotoxic assays.

For ex vivo cytotoxic assays, BRL1468 cells (primary cell line established from the tumor tissue of a PyMT animal) were used as target (T) cells and CD45^+^ cells as effector (E) cells. On day 1, 1 × 10^4^ BRL1468 cells were seeded in black p96-well plates (Greiner Bio-One, 655087). The next day, tumor tissue was processed as described previously in Methods, and CD45^+^ cells were isolated by positive selection using mouse CD45 MicroBeads (Miltenyi Biotec, 130-052-301) in an AutoMacs Pro Separator (Miltenyi Biotec). Assessment of the purity of the isolated cell population was done by flow cytometry (over 90%). Cells were seeded at a 3E:1T ratio in triplicates in complete RPMI-1640 medium (Gibco, 11875085) (10% FBS, 1% penicillin/streptomycin, 1% l-glutamine, and 50 μM β-mercaptoethanol). Coculture was maintained unstimulated or stimulated with anti-mouse CD3 (1 μg/mL, BioLegend, 100202) and CD28 (0.5 μg/mL, BioLegend, 102102). After 72 hours, nonattached effector cells were removed from wells by multiple washings. Viability of remaining tumor cells was determined using CellTiter-Glo (Promega, G7570). Percentage of relative cytotoxicity induced by effector cells was calculated as 1 – [(average E:T luminescence – blank)/(average luminescence target cells alone – blank)]·100. Potency of activation was calculated as a ratio comparing the cytotoxic effect under an activated state to the baseline state (basal conditions).

### TAM and splenocyte ex vivo cocultures.

For ex vivo cocultures of TAMs and splenocytes, tissues were processed as described in previous sections. Next, TAMs were sorted as described previously in Methods. For splenocyte isolation, spleen was harvested, mechanically smashed, and passed through a 40 μm strainer to obtain single-cell suspensions. Cells were cocultured at a 5:1 ratio (splenocytes/TAMs) in complete DMEM medium under the following conditions: splenocytes alone (control); splenocytes + control-derived TAMs; splenocytes + anti-VEGFA LH-derived TAMs; splenocytes + anti-VEGFA HH-derived TAMs. After 48 hours, 1× Brefeldin A (BioLegend, 420601) was added, and the supernatants were collected for cytokine measurement. Cells were stained as described in previous sections to determine the percentage of IFN-γ^+^ and Ki67^+^ T cells, which indicates the presence of activated T cells.

### scRNA-seq.

Tumor samples were processed as previously described. Then, debris and dead cells were excluded by sorting DAPI^–^ cells in a BD FACSAria (BD Biosciences). Subsequently, cell multiplexing oligo labeling was conducted using the 3′CellPlex Kit Set A (10x Genomics, 1000261) following the manufacturer’s instructions. Next, a secondary sort of DAPI^–^ cells was executed with the BD FACSAria to further enrich for viable cells. Multiplexed single-cell suspensions sourced from 3 distinct tumors were processed together into 1 sample, which was drop encapsulated with a Chromium device (10x Genomics). The generated libraries were later sequenced using Illumina Nextseq 550 (with v2.5 reagent kits) and post-processed with Cell Ranger software (10x Genomics) to obtain a scRNA-seq expression matrix for each sample.

### Retrospective analysis of clinical trials.

RNA gene expression data from the phase III JAVELIN 101 trial was obtained from Shojaei et al. (24) and data from the phase III IMbrave 150 clinical trial from Fan et al. (27). In these data sets, a hypoxia score was obtained for each patient by a signature defined by the average expression of canonical hypoxia-regulated genes *Vegfa*, *Ca9*, and *Slc2a1*. Then, patients were classified as LH and HH, divided by the median of the hypoxia score distribution in each cohort. Finally, gene expression of *SPP1* and survival analysis were tested in the subgroups by log-rank test.

Collected tumor samples from the NCT01484080 clinical trial (ClinicalTrials.gov) were used for retrospective gene expression analysis (14). Total RNA from tumors in paraffin-embedded blocks was isolated using an RNeasy FFPE kit (Qiagen, 73504) according to the manufacturer’s protocol. RNA quality was determined by Agilent’s 2100 Bioanalyzer Lab-Chip technology. The reads were then aligned to the GRCh38 genome assembly using STAR 2.7.10b (https://github.com/alexdobin/STAR/releases/tag/2.7.10b; commit ID: 9ec8b7e). The results were quantified using the featureCounts tool from the Rsubread 2.0.3 package (https://www.bioconductor.org/packages/release/bioc/html/Rsubread.html). The gene counts obtained from different sequencing rounds from the same sample were added together to merge the results. GSEA versus Molecular Signatures Database v7.5.1 collection was performed on a ranked list of DESeq2 data, where log_2_FC of genes showing greater than 1.2 absolute fold change was divided by their corresponding *P* value. For analysis, patients were classified in *Spp1* gene expression basal high and low according to the distribution median of *Spp1* gene expression. Then, differential gene expression analysis was performed with DESeq2 pipeline between samples before and after treatment in each subgroup. For the GSEA, we consulted the Molecular Signatures Database v7.5.1 collection and applied it to a ranked differentially expressed gene list of DESeq2 data, wherein log_2_FC of genes exhibiting an absolute fold change greater than 1.2 was divided by their corresponding *P* value.

### Statistics.

Statistical analyses were performed with GraphPad Prism 10.2.3 software. In vitro and in vivo data are presented as mean ± SEM. Significant statistical differences between 2 groups were determined using paired or unpaired 2-tailed Student’s *t* test as detailed in the figure legends. Correlation analyses were performed using Spearman’s test. Differences in tumor growth curves between groups were analyzed by 2-way ANOVA followed by Tukey’s or Šidák multiple-comparison test. One-way ANOVA with Tukey’s post hoc test was performed for multiple comparisons between groups. Differences in *SPP1* expression in the clinical trial were analyzed by 2-way ANOVA. OS changes were calculated by log-rank test. Data with *P* value < 0.05 were considered statistically significant.

### Study approval.

All animal experiments were performed at CNIO (Spanish National Cancer Research Center) in accordance with protocols approved by the Research Ethics and Animal Welfare Committee on Animal Experimentation of the Instituto de Salud Carlos III and Dirección General de Agricultura, Ganaderia y Alimentación de la Comunidad de Madrid (PROEX 387/15 and PROEX 206.7/21). Experiments were performed in accordance with the guidelines stated in the International Guiding Principles for Biomedical Research Involving Animals developed by the Council for International Organizations of Medical Sciences.

### Data availability.

The data supporting the findings of this study are available within this article, in the supplemental material, and in the Supporting Data Values file. Mouse RNA-seq data were deposited in the NCBI GEO under accession number GSE241539. scRNA-seq data were deposited in the NCBI GEO under accession number GSE243273. Scripts to reproduce the analyses and figures are publicly accessible at https://github.com/cnio-ccg/breast_cancer_singlecell_collab (commit ID: 0334142). An interactive ShinyCell portal providing cell annotations, hypoxia percentage, and UMAP visualization is available at https://sunshine.bioinformatics.cnio.es/groups/compgenomics/Mouse_BreastCancer_Hypoxia_Study_final/ Human RNA-seq data from the NCT01484080 clinical trial have been previously deposited in the NCBI GEO database with the accession number GSE255359. RNA-seq data from the in vitro coculture assay were deposited in the NCBI GEO under accession number GSE328461.All additional processed data supporting the conclusions of this work are available from the corresponding author upon reasonable request.

## Author contributions

JLRS, RJ, and MQF conceptualized and design the study. JLRS, VJR, MJB, SM, and M Muñoz performed experiments and/or analyzed data. JLRS, M Moradiellos, LDG, LGJ, SP, AWW, GM, and MQF performed and supervised the in silico analysis. MJB, SM, and VJR supervised and/or performed animal experiments. JLRS, RJ, and MQF analyzed the data. All authors wrote, reviewed, and approved the manuscript.

## Conflict of interest

The authors have declared that no conflict of interest exists.

## Funding support

- Instituto de Salud Carlos III, cofunded by the European Regional Development Fund (ERDF): AES PI19/00454 and PI22/00317 (to MQF).
- Instituto de Salud Carlos III and the European Union (NextGeneration EU/PRTR): Proyectos de Investigación de Medicina Personalizada PMP22/00032 (to MQF).
- Madrid Regional Government, ERDF; Call for Coordinated Research Groups from Madrid Region: B2017/BMD3733 (Immunothercan-CM) (to MQF).
- Eva Plaza/CNIO Friends Post-Doctorate Fellowship and Marie Sklodowska-Curie Individual Fellowship 893597 (to RJ).
- Ministerio de Ciencia, Innovación y Universidades/Agencia Estatal de Investigación: FPI Grant–Severo Ochoa (SEV-2015-0510-19-4): PRE2019-087354 (to JLRS).
- CRIS Contra El Cancer Foundation (donation).

## Supplementary Material

Supplemental data

Unedited blot and gel images

Supporting data values

## Version 1 07/15/2026

Electronic publication
