# Supplementary material for: Osteopontin mediates acquired resistance to hypoxia-inducing antiangiogenics and promotes anti–PD-L1 refractoriness in breast cancer models
Source: J Clin Invest. 2026 Jul 15;136(14):e174092. doi: 10.1172/JCI174092 (PMC13367972; doi:10.1172/JCI174092)
Supplement: Supplemental data [file jci-136-174092-s062.pdf]

Supplemental Material

Osteopontin mediates acquired resistance to hypoxia-inducing antiangiogenics and promotes anti-PD-L1 refractoriness in breast cancer models

Supplemental Figures

Supplemental Figure 1

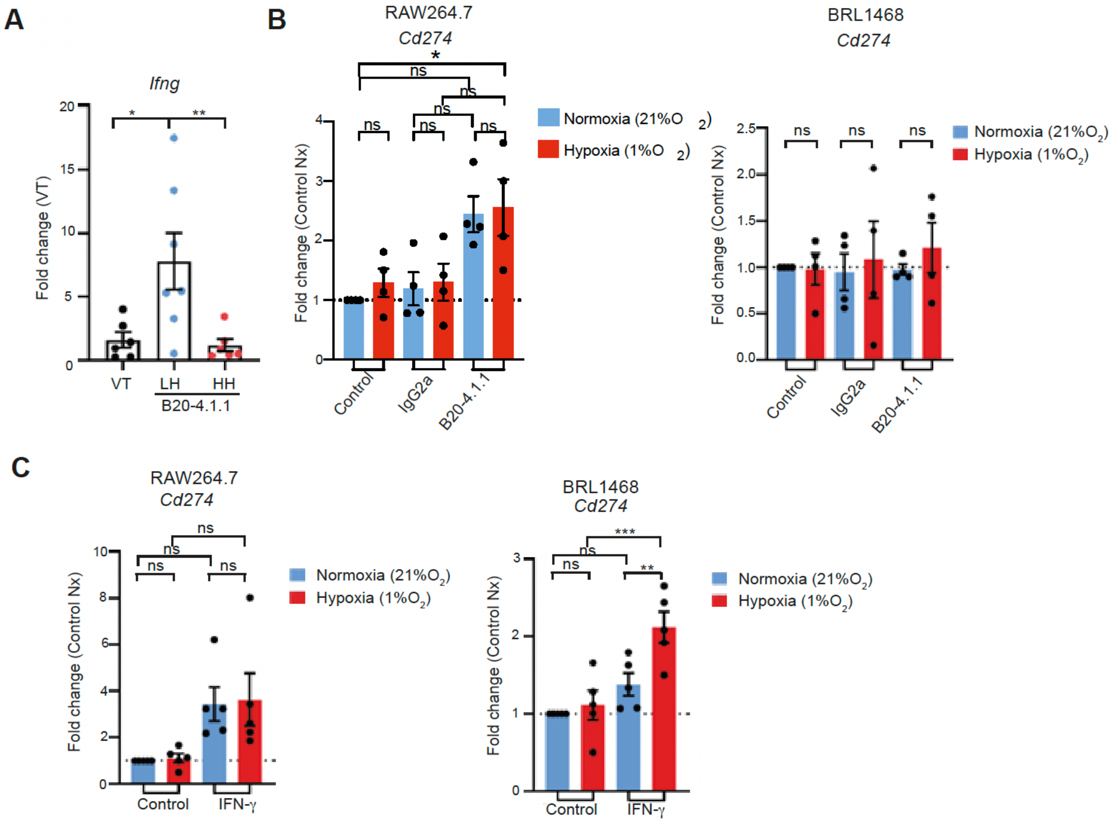

Supplemental Figure 1. in vitro PD-L1 regulation by IFN-γ and/or hypoxia. (A) IFN-γ transcriptional levels were elevated in B20-4.1.1- versus isotype control-treated tumors, although only in those developing LH (n=6) One-Way ANOVA with Tukey´s post hoc test for multiple comparisons. **(B)** Fold-regulation (referenced to isotype-control treated cells) of PD-L1 expression levels in response to exposure to 1% O2, in presence or absence of B20-4.1.1, in a monocyte cell line (RAW264.7) (n= 4-5) or in a PyMT tumor-derived primary cell line (BRL1468) (n= 4-5). Two-Way ANOVA with Tukey´s post hoc test for multiple comparisons. **(C)** Fold-regulation of PD-L1 in the same cell lines (RAW264.7: n=5; BRL1468: n=5) in response to IFN- γ. Data represent mean ± SEM. Two-Way ANOVA with Tukey´s post hoc test for multiple comparisons. *: *P*<0.05; **: *P*<0.001; ns: non-significant**.**

Supplemental Figure 2

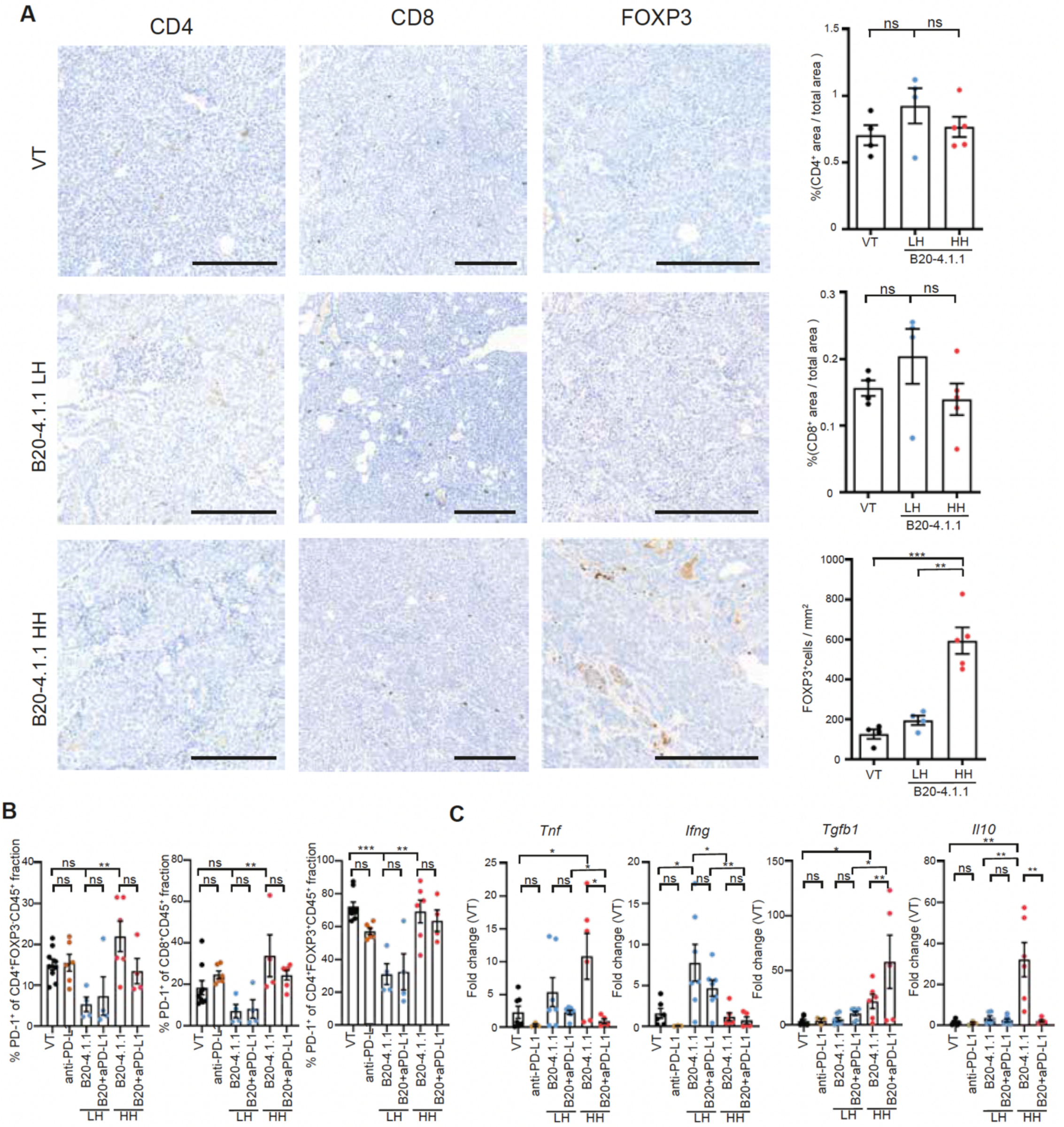

Supplemental Figure 2. Extended lymphoid characterization of LH and HH tumors. (A) Immunohistochemical staining of CD4, CD8 and FOXP3 in HH and LH tumors. The images and the quantitation charts suggest increased CD4 and CD8 lymphocyte infiltration combined with decreased Treg infiltration in normoxic areas, compared to hypoxic areas (n=4-5). Representative images from each condition are shown. Scale bars: 200μm. **(B)** PD-1-positivity among the same lymphocyte subsets as in main Figure 2D. **(C)** Cytokine profile in the same tumors, showing persistence of an adverse immune milieu in HH tumors despite PD-L1 blockade. Data represent mean ± SEM. One-Way ANOVA with Tukey´s post hoc test for multiple comparisons. *: *P*<0.05; **: *P*<0.01; ***: *P*<0.001; ns: non-significant**.**

Supplemental Figure 3

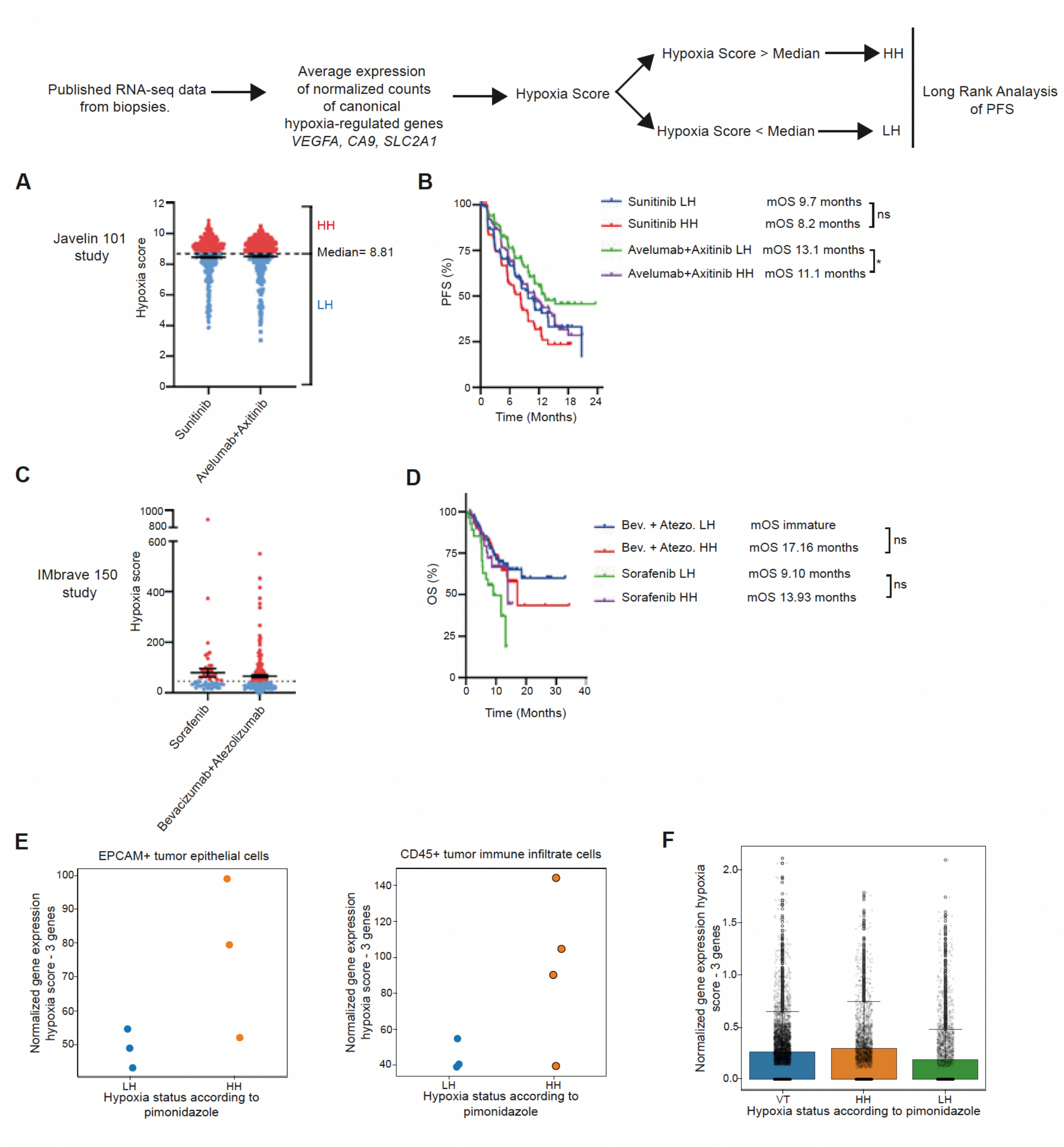

Supplemental Figure 3. Role of hypoxia in the PFS and OS benefit in the Javelin 101 and IMbrave 150 Phase 3 randomized clinical trials in kidney and liver cancer. A “hypoxic score” based in the normalized counts of hypoxia-regulated genes (Vegfa, Ca9 and Slc2a1) was calculated for each patient with gene-expression data available enrolled in the Javelin 101 and in the IMbrave 150 trials. The first one compared the efficacy of standard treatment with the antiangiogenic agent sunitinib versus the combination of the antiangiogenic agent axitinib plus the PD-L1 inhibitor avelumab. The second trial compared standard treatment with the antiangiogenic agent sorafenib against the experimental treatment with the antiangiogenic agent bevacizumab plus the anti-PD-L1 agent atezolizumab. Patients were split in “high hypoxia” or “low hypoxia” (HH and LH) according to whether their score was above or below the average in each trial. **(A)** Chart depicting the hypoxia score distribution among both treatment arms of the Javelin 101 trial (n=690 each group). **(B)** PFS of each treatment arm split by HH or LH status in the Javelin 101 trial. **(C)** Chart depicting the hypoxia score distribution among both treatment arms of the IMbrave trial (n=270 each group). **(D)** Overall survival of

patients in each treatment arm split by HH or LH status. **(E)** In the animal experiments, hypoxia could be detected by staining pimonidazole, which is infused 60 minutes before sacrifice. Since this is not possible in humans, the 3-gene hypoxia signature was computed. In order to check inter-species concordance, the same 3-gene signature was determined from two experiments also described in the main text: bulk gene-expression of the CD45-positive (tumor infiltrating leukocytes) and the EpCAM-positive (tumor epithelial compartment) tumor fractions, and single-cell RNA-seq from tumors, classified as HH or LH according to pimonidazole in response to antiangiogenic treatment. The plots in this panel show normalized scores for this signature in bulk RNA-seq of LH and HH tumors in the $EpCAM^+$ (left) and $CD45^+$ (right) compartments, evidencing higher scores in HH tumors. Each dot represents one tumor; boxes indicate median and IQR. **(F)** Concordance in scRNA-seq: normalized scores of the same 3-gene signature (*Ca9*, *Slc2a1*, *Vegfa*) across tumors obtained from isotype-treated tumors or classified as LH or HH by pimonidazole following B20-4.1.1 treatment; horizontal bars represent the median values (0.140, 0.174 and 0.122 for vehicle, HH and LH, respectively; Mann-Whitney 0.012).

Supplemental Figure 4

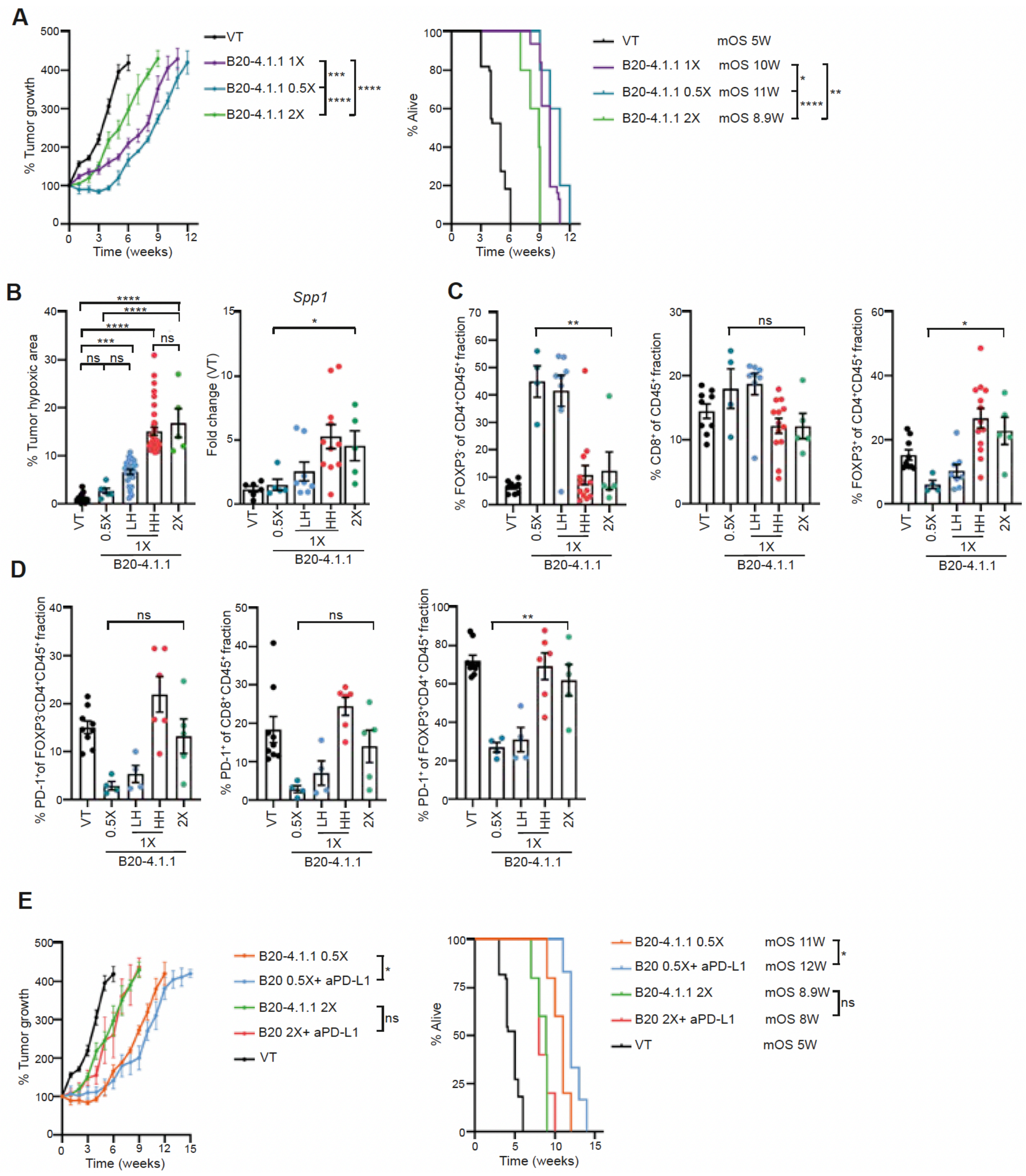

Supplemental Figure 4. Dose-response effects of B20-4.1.1 in antitumor effect, hypoxia, immune infiltrate and efficacy of combination with anti-PD-L1 treatment. **(A)** Left-hand side chart: tumor growth charts of VT (n=36), B20-4.1.1 at 0.5X (n=5), 1X (n=30) and 2X (n=5) of the standard dose; right-hand side chart: effect of the same treatments in mice OS. Two-way ANOVA followed by Sidak's multiple-comparison test. **(B)** Left panel: Percentage of the tumor areas positive for pimonidazole (hypoxia) staining in the former treatment groups. The range and interquartile spread (IQR) were as follows for each treatment group: 0.5X (n=6): range: 1-5%; IQR: 2-3%; 1X (LH n=24; HH n=36): range 0-36%; IQR: 7.25-16%; 2X (n=5): range 11-27%; IQR 11.5%-23.5%. Right panel: Tumor *Spp1* transcript levels across the indicated treatment conditions. VT: n=6; B20 0.5X: n=5; B20 LH 1X: n= 10; B20 HH 1X: n= 9 and B20 2X: n=5.
One-Way ANOVA with Tukey´s post hoc test for multiple comparisons. **(C)** Percentage of CD4-, CD8- and FOXP3-positive T-cells isolated from tumors of the three treatment

groups (plus isotype control) (n=4-13). One-Way ANOVA with Tukey´s post hoc test for multiple comparisons. **(D)** Same comparison as in **(C)** regarding PD1 expression in the same cell types (n=4-9). One-Way ANOVA with Tukey´s post hoc test for multiple comparisons. **(E)** Efficacy of adding anti-PD-L1 treatment in animals treated with 0.5X or 2X B20-4.1.1 dosage (n=5-36). Two-way ANOVA followed by Turkey's multiple-comparison test.*: $P<0.05$; **: $P<0.01$; ***: $P<0.001$; ****: $P<0.0001$; ns: non-significant. Data represent mean ± SEM.

Supplemental Figure 5

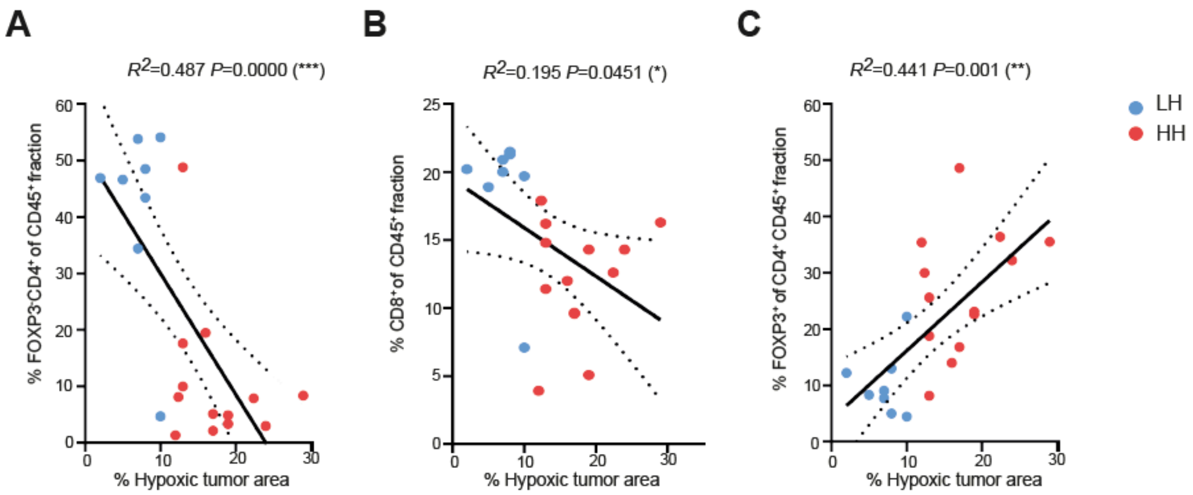

Supplemental Figure 5. Correlation between hypoxia levels and lymphocytes in response to treatment. (A) CD4, **(B)** CD8 and **(C)** Treg lymphocytes proportion of the CD45 infiltrate according to the percentage of hypoxic tumor area. Red dots represent HH tumors (n=13), and blue dots represent LH tumors (n=8). Pearson´s R correlation coefficient and P-values are shown for each correlation.

Supplemental Figure 6

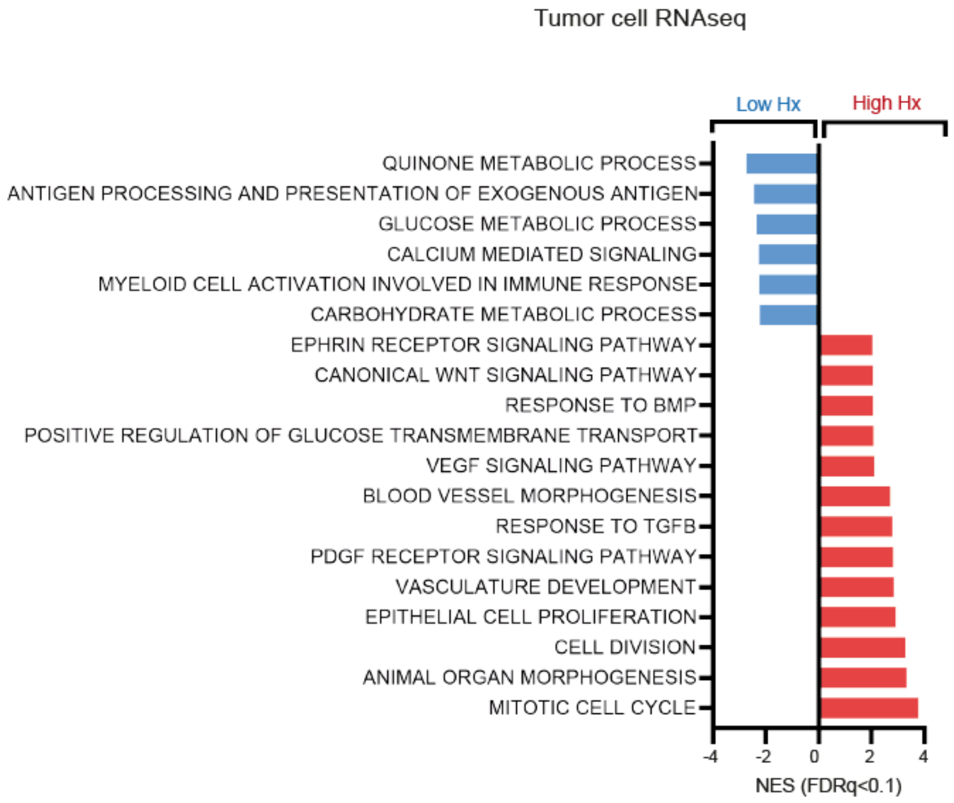

Supplemental Figure 6. GSEA of tumor cells ($CD45^-$/$EPCAM^+$ compartment) obtained from HH and LH tumors. Pathways upregulated in LH (blue bars) and HH (red bars) tumor cells, respectively. This bar plot depicts all the pathways that had a false discovery ratio below 0.1 (FDRq). NES: normalized enrichment score.

Supplemental Figure 7

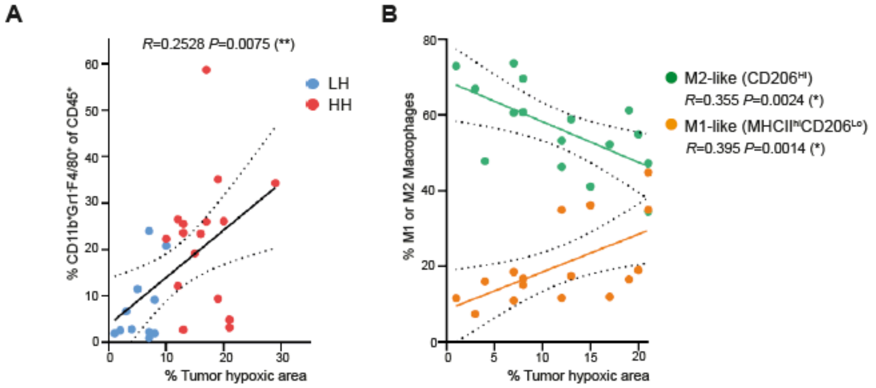

Supplemental Figure 7. Distribution of M1 and M2 macrophages in LH and HH tumors. (A) Correlation between macrophages percentage and tumor hypoxia (LH n=12, HH n=15). **(B)** Correlation between the tumor hypoxic fraction and M1 or M2 percentages (n=16 each group). Pearson´s R correlation coefficient and P-values are shown for each correlation. **: *P*<0.01.

Supplemental Figure 8

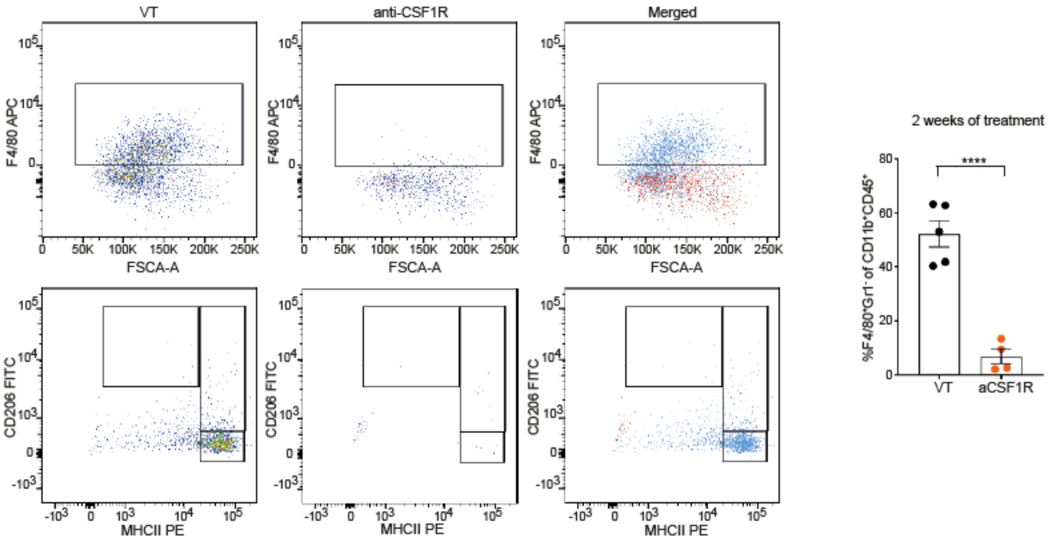

Supplemental Figure 8. Tumor macrophage depletion by anti-CSF1R antibodies. F4/80-positive (upper rows, representing the total amount of macrophages) obtained from whole tumors, and CD206/MHCII levels in those cells (lower rows), from control- or anti-CSF1R-treated animals (this plot shows that anti-CSF1R depleted all macrophage subpopulations). The dot-plots are merged in the right-hand side charts in order to compare the abundance. The chart in the far right shows the proportion of macrophages among myeloid cells isolated from tumors treated with control (n=5) or anti-CSF1R antibody (n=4). Data represent mean ± SEM. Unpaired t test. ****: $P<0.0001$.

Supplemental Figure 9

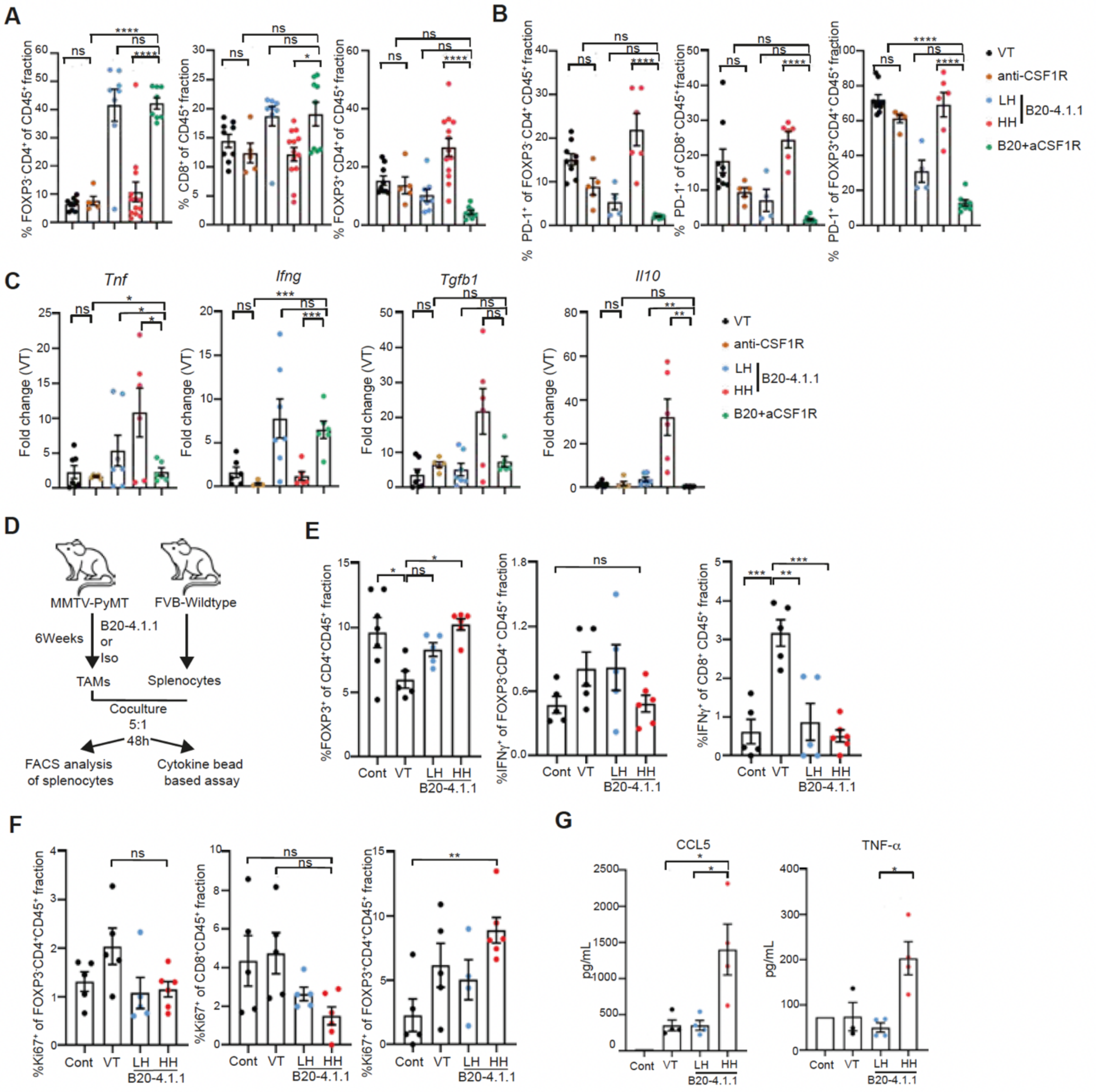

Supplemental Figure 9. Changes in the tumor immune infiltration fraction induced by macrophage depletion, and effects of TAMs on immature lymphocytes ex vivo. **(A)** Relative changes in CD4, CD8 and Treg fractions among the CD45-positive tumor fraction, comparing VT (n=9)-, anti-CSF1R (n=5)-, B20-4.1.1 (LH n=8; HH n=13)- and combo (n=8)-treated animals. **(B)** Comparison of the PD-1 levels in the same lymphocytes and treatments as in **(A)**. **(C)** Comparison of the transcriptional levels of *Il10*, *Tgfb*, *Cd274*, *Tnf* and *Ifng* in the same treatment groups as **(A)** (n=5-6). **(D)** TAMs extracted from PyMT tumors from wild-type FVB animals treated with control or B20-4.1.1 were co-cultured (5:1) for 48 hours with fresh splenocytes obtained from wild-type FVB animals. **(E)** Changes in the lymphocyte profile (CD4, CD8 or Tregs) recovered after the co-culture of splenocytes with TAMs extracted from control (n=7), VT (n=5) or -treated or HH (n=6)/LH(n=5) tumors. **(F)** Ki67 replicative fraction measured in the same lymphocytes from the former experiment. **(G)** CCL5 and TNF-α isolated from the co-cultures of TAMS from B20-4.1.1-treated or vehicle-treated tumors and wild-type splenocytes supernatants (n=3-4). Data represent mean ± SEM. One-Way ANOVA with Tukey´s post hoc test for multiple comparisons. *: P<0.05; **: P<0.01; ***: P<0.001; ****: P<0.0001; ns: non-significant.

Supplemental Figure 10

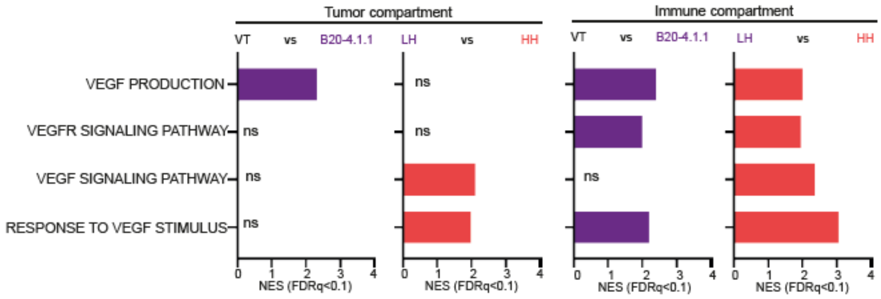

Supplemental Figure 10. Significant angiogenesis GSEA in B20-4.1.1-treated tumors. RNAseq data of the EPCAM+ and the CD45+ tumor compartments are shown in the left- and right-hand side panels. In each panel, GSEA score of significantly regulated gene expression clusters are shown for B20-4.1.1-treated tumors versus isotype-treated tumors and HH vs LH (B20-4.1.1-treated) tumors. NES: normalized enrichment score. Only GSEAs with false discovery ratio (FDR) < 0.1 are shown.

Supplemental Figure 11

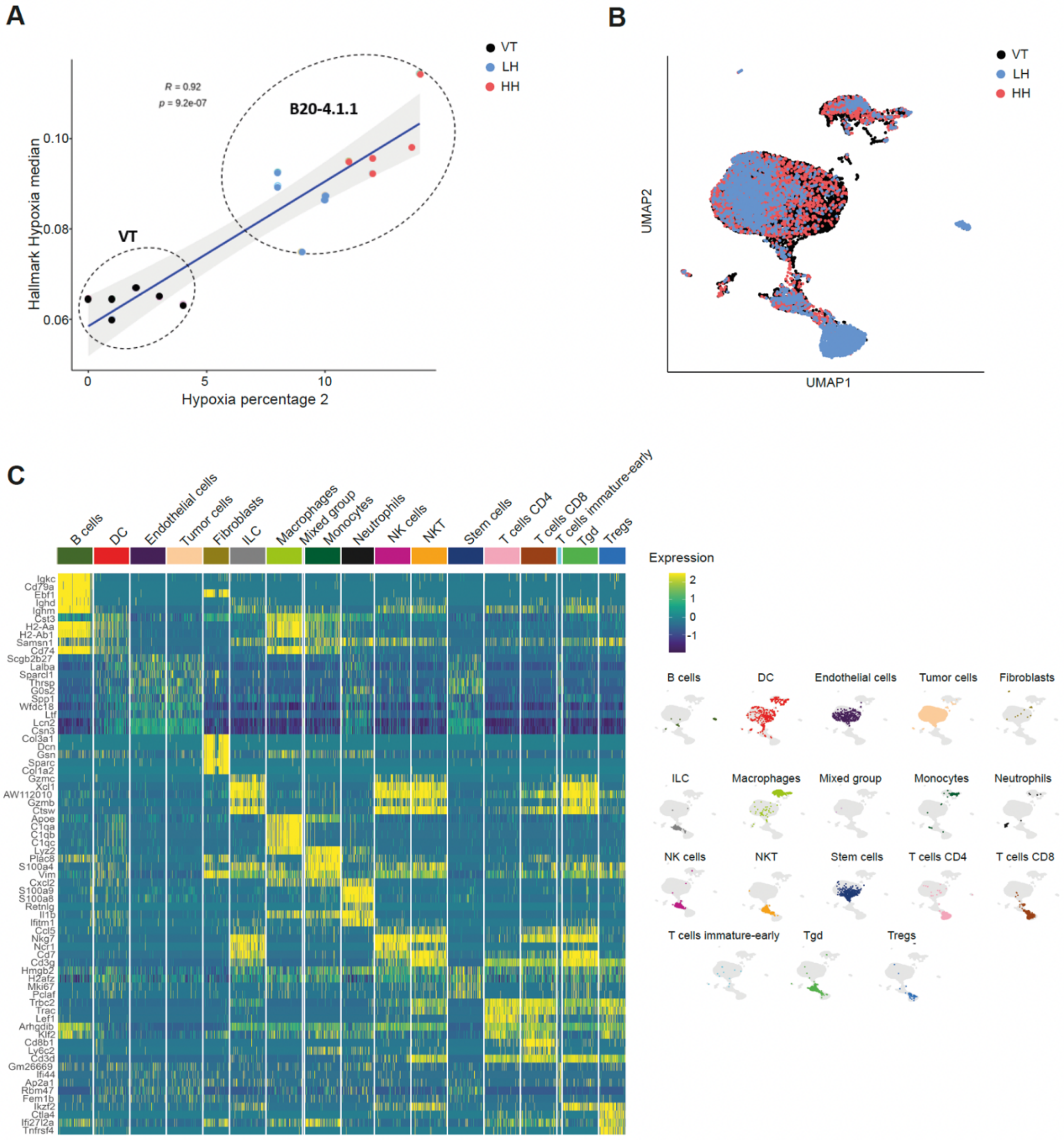

Supplemental Figure 11. Single-cell RNAseq data extended data. (A) Correlation between hypoxia gene cluster and pimonidazole, gene expression clusters by cell type and among macrophage subtypes. Expression score of the transcripts included in the Hypoxia GSEA cluster calculated in single-cell RNAseq data, according to whether the cells belonged to a VT or HH/LH B20-4.1.1-treated tumor. Pearson´s R correlation coefficient and P-value are shown. **(B)** Differential gene expression among the 18 identified cell types in the experiment. The heatmap indicates the top-5 highest expressed Suerat-calculated markers per each cell type. **(C)** Cell distribution according to the tumor-of-origin (tumors treated with isotype, or HH/LH antiangiogenic-treated tumors).

Supplemental Figure 12

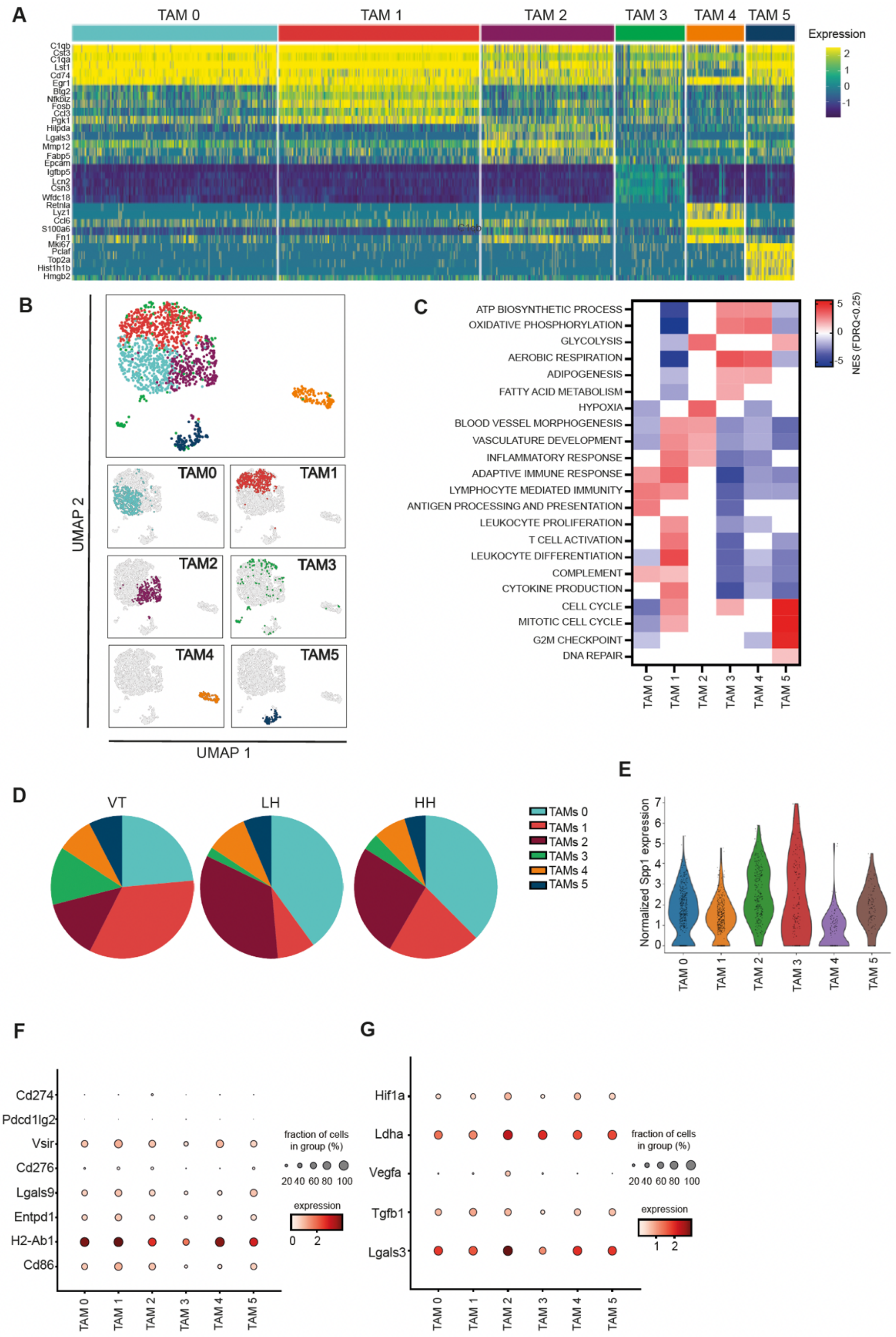

Supplemental Figure 12: Single-cell RNAseq data reveal different TAM subtypes with diverse functional programs according to tumor hypoxia levels. (A) Differential gene expression across the 6 identified TAM subtypes. The heat map shows the top five Seurat-identified marker genes for each cell type. **(B)** Sub-clustering analysis of macrophages: an UMAP plot of the macrophage subpopulations in the experiment, together or split by TAM subtype. **(C)** Plot indicating the main GSEAs upregulated in each of the 6 isolated TAM subtypes. **(D)** Distribution of the different TAM subtypes

across the different treatment subgroups (from left to right, in VT tumors, or in antiangiogenic-treated LH and HH tumors. **(E)** Violin plot of *Spp1* expression across TAMs, showing highest expression in TAM2 and TAM3 (all comparisons of *Spp1* levels across TAM pairs are statistically significant – two-sided, Bonferroni-corrected Wilcoxon test – except from TAM1 vs. TAM6, TAM6 vs. TAM4 and TAM3 vs. TAM4). **(F)** Dot plot of selected immunoregulatory genes across TAMs. Dot size represents fraction of cells expressing the gene and color indicates scaled mean expression. **(G)** Dot plot of selected hypoxia/metabolic and microenvironment-shaping genes across TAMs. Dot size represents fraction of cells expressing the gene and color indicates scaled mean expression.

Supplemental Figure 13

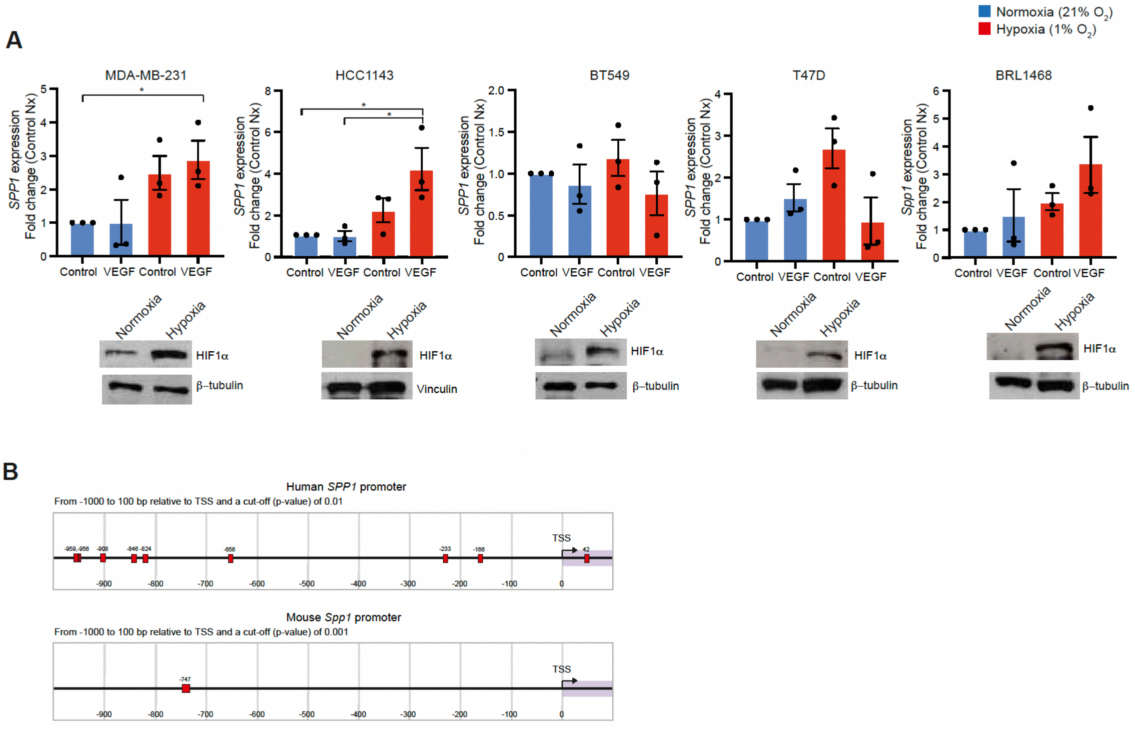

Supplemental Figure 13: Hypoxia triggers SPP1 up-regulation in breast cancer cell lines. (A) *Spp1* qRT-PCR in response to VEGF or vehicle, in normoxia or hypoxia, in several breast cancer cell lines. Experiments were performed in triplicate. Control of hypoxia exposure (HIF1-α stabilization demonstration by western blot) is shown below. Data represent mean ± SEM. Two-Way ANOVA with Tukey´s post hoc test for multiple comparisons. *: $P<0.05$; ns: non-significant. **(B)** HIF1-α binding sites in the human and murine *Spp1* promoter region.

Supplemental Figure 14

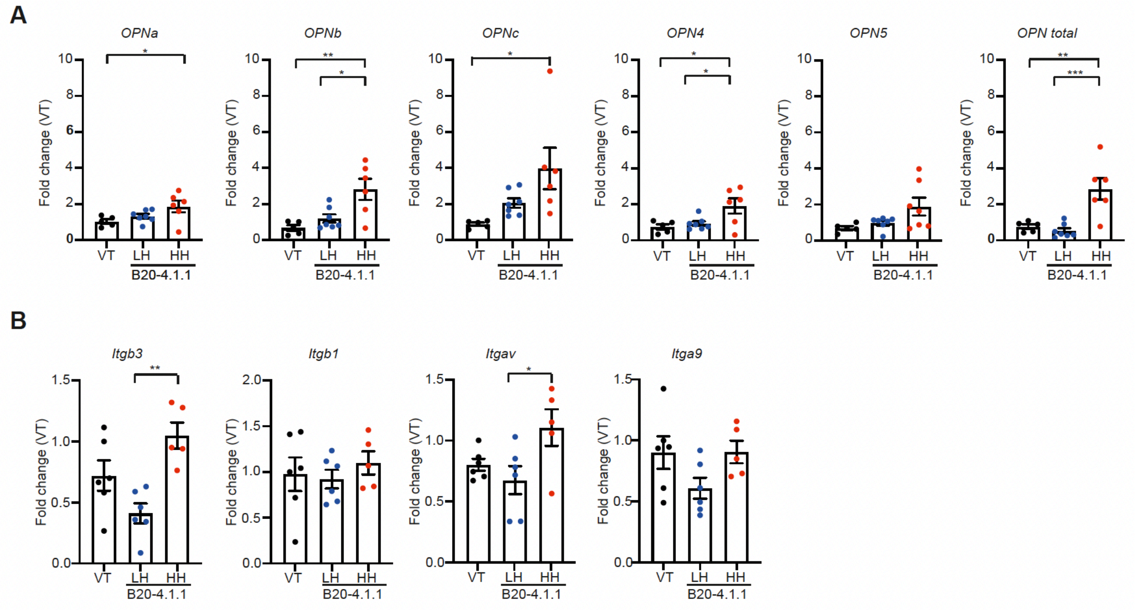

Supplemental Figure 14: Osteopontin and integrin isoforms in response to antiangiogenic treatment. There are 5 annotated *Spp1* isoforms: (OPNa, OPNb, OPNc, OPN4 and OPN5; a is the full-length transcript; b lacks exon 5; c lacks exon 4; 4 lacks exons 4 and 5; and 5 is the full-length protein plus a retained fragment from intron 3). **(A)** Total and isoform analysis in tumors treated with vehicle or antiangiogenic, developing HH or LH (n=5-7). **(B)** Same analysis as in (A) for integrins B3, B1, apha V and alpha 9, which form the alpha V beta 3 and alpha 9 beta 1 heterodimers (n=5-6). Data represent mean ± SEM. One-Way ANOVA with Tukey´s post hoc test for multiple comparisons. *: P<0.05; **: P<0.01; ***: P<0.001; ns: non-significant.

Supplemental Figure 15

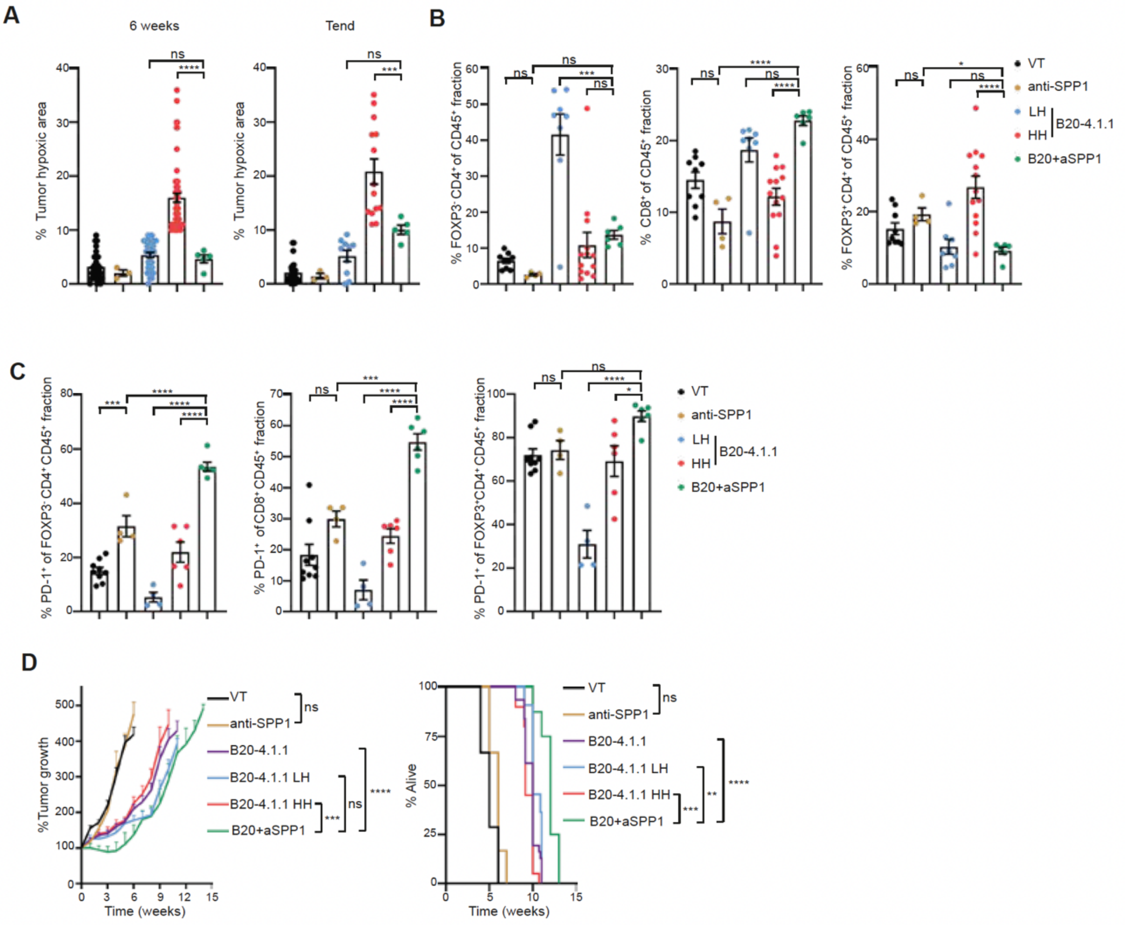

Supplemental Figure 15: Depleting SPP1 reverts the immunosuppressive microenvironment. (A) Tumor hypoxia levels at an intermediate and humane endpoint in the different treatment groups (n=3-57). One-Way ANOVA with Tukey´s post hoc test for multiple comparisons. **(B)** $CD4^{+}$, $CD8^{+}$ and $FOXP3^{+}$ T cells in the tumor immune infiltrate among the different treatment subgroups (N=4-13). One-Way ANOVA with Tukey´s post hoc test for multiple comparisons. **(C)** PD-1 levels in CD4, CD8 and Treg cells in each treatment group (N=4-13). One-Way ANOVA with Tukey´s post hoc test for multiple comparisons. **(D)** Tumor growth and Kaplan-Meier curves for animals treated with VT (n=36), anti-SPP1 (n=5), B20-4.1.1 (LH (n=10) and HH (n=20)), and anti-SPP1+B20-4.1.1 (n=8). Two-way ANOVA followed by Turkey's multiple-comparisons test. Data represent mean ± SEM. *: $P<0.05$; **: $P<0.01$; ***: $P<0.001$; ****: $P<0.0001$; ns: non-significant.

Supplemental Figure 16

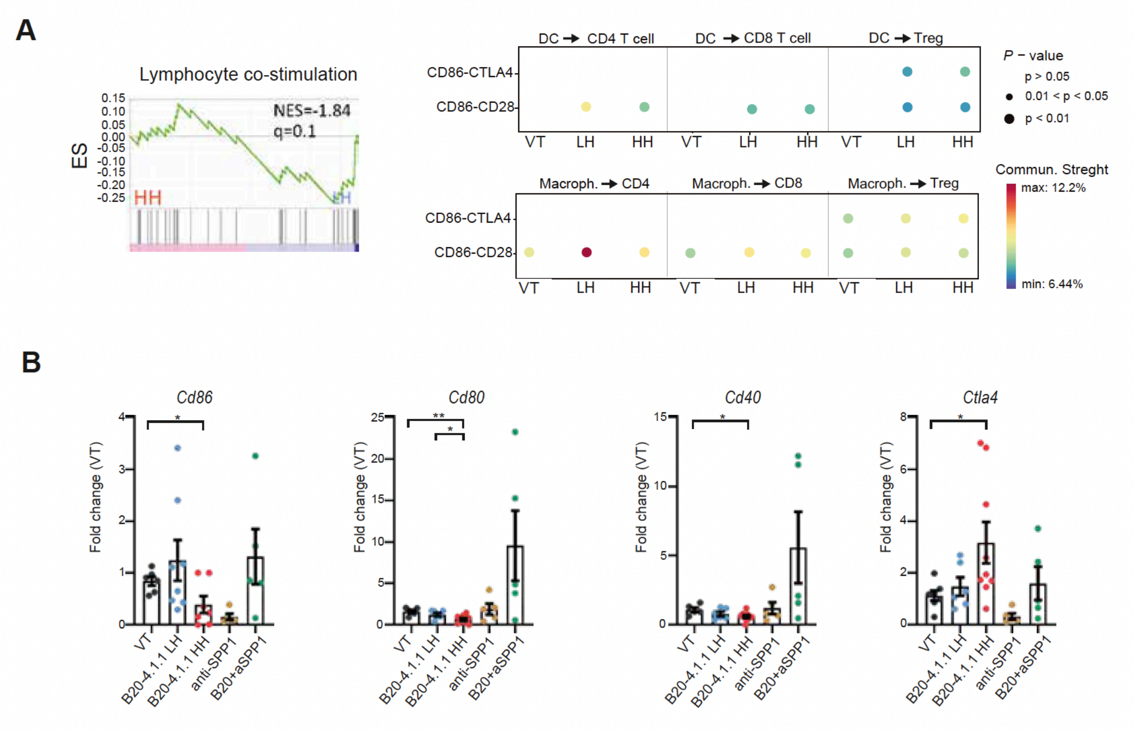

Supplemental Figure 16. Single-cell RNAseq analysis of lymphocyte co-stimulation in HH and LH tumors: **(A)** Lymphocyte co-stimulation GSEA in HH versus LH. Right: ligand-receptor communications in the CD86 network: bubble plot showing the differential communication strength and *P* value for the CD86-CTLA4 and CD86-CD28 axes between the 6 depicted sender-receiver cell type pairs between isotype-treated and HH or LH B20-4.1.1-treated tumors. The P-value of the interaction is represented by the dot size, whereas its strength is represented in a colored gradient. **(B)** Increase of CD80, CD86 and CD40 and decrease in CTLA4 co-stimulation molecules in SPP1-depleted tumors compared to HH tumors; SPP1 depletion alone and isotype control-treated animals are shown for control purposes (n=4-9). Data represent mean ± SEM. One-Way ANOVA with Tukey´s post hoc test for multiple comparisons. *: *P*<0.05; **: *P*<0.01.

Supplemental Figure 17

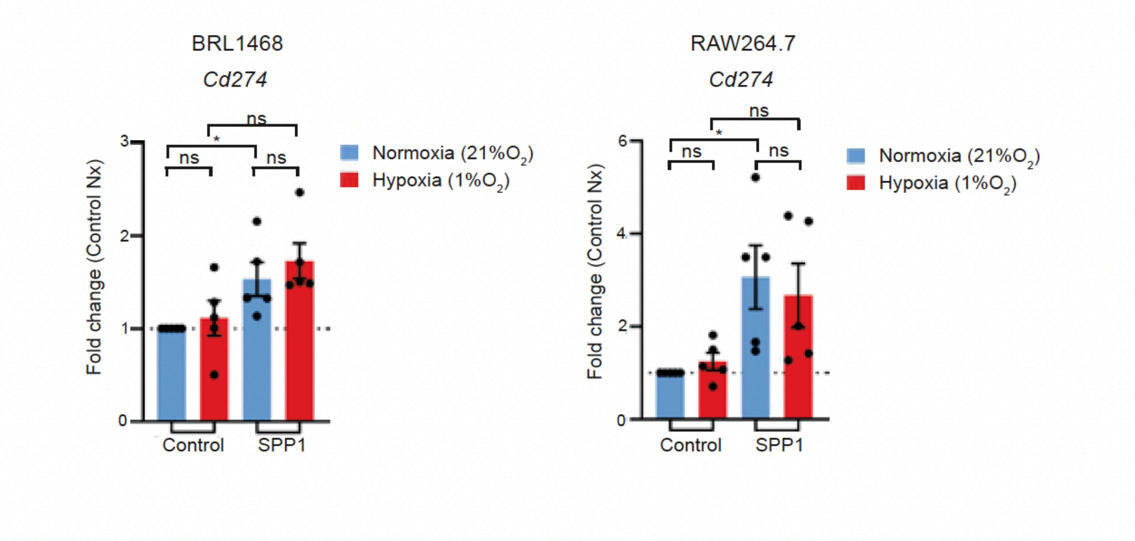

Supplemental Figure 17. PD-L1 regulation in response to SPP1. In vitro exposure of the breast cancer cell line BRL1468 and the macrophage cell line RAW264.7 showed that SPP1 was a stronger inducer of PD-L1 expression than hypoxia (n=5 each group). Data represent mean ± SEM. One-Way ANOVA with Tukey´s post hoc test for multiple comparisons. *: *P*<0.05; **: *P*<0.01; ns: non-significant.

Supplemental Figure 18

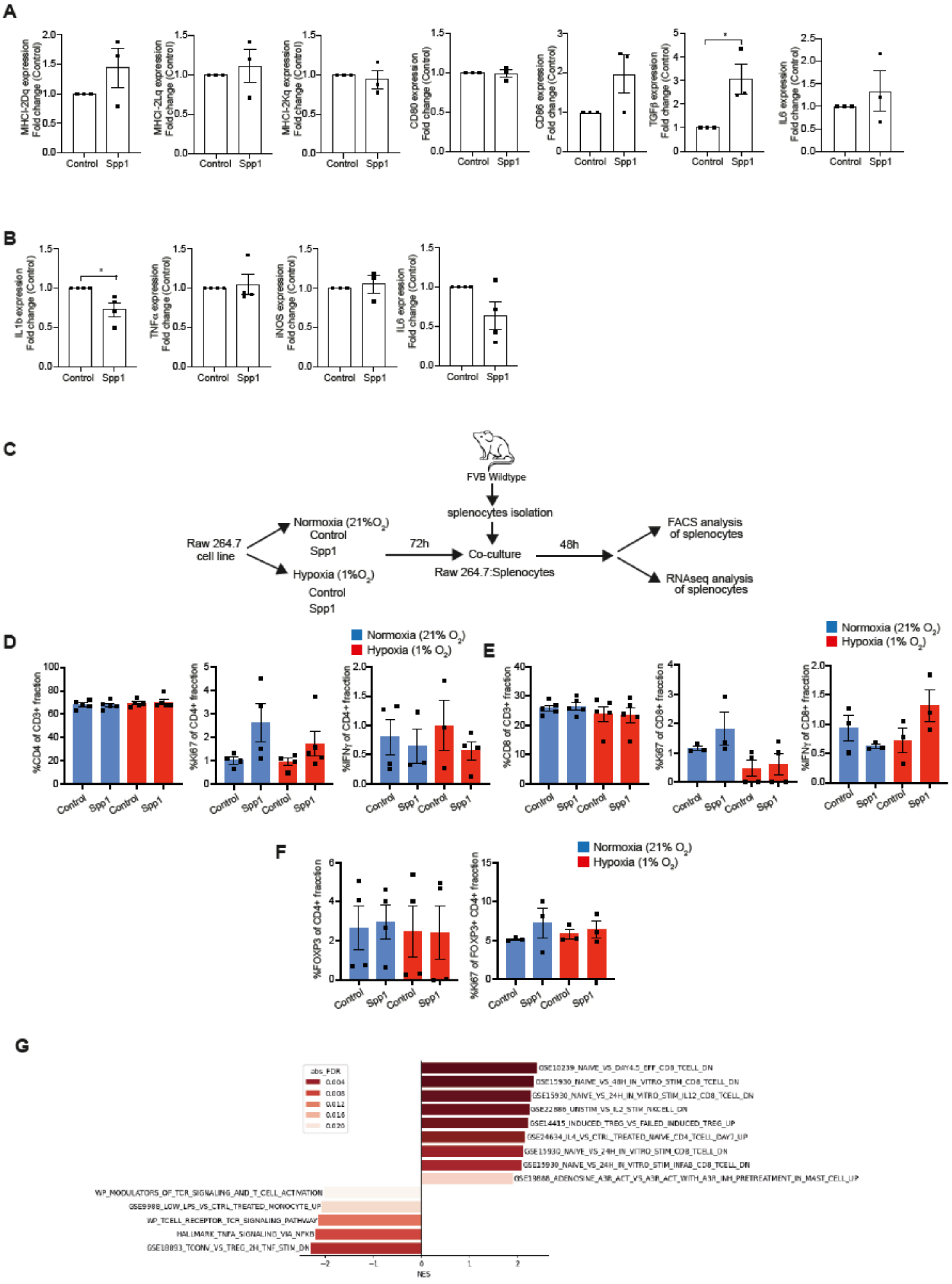

Supplemental Figure 18: Immunosuppressive effects of SPP1 in tumor epithelium and immune infiltrate (A) Changes in antigen-presentation molecules and cytokine secretion in tumor BRL1468 cells in response to SPP1. Experiment was performed in triplicate (n=3). Unpaired t test. *: $P<0.05$. **(B)** Induction of M2-like profile in RAW246.7 cells by SPP1. Experiment was performed, at least, in triplicate (n=3-4). Unpaired t test. **(C)** Co-culture schedule: naïve splenocytes from FVB animals were co-culture with

RAW246.7 that were pre-exposed to SPP1 or vehicle, in hypoxia or normoxia, for the indicated times. Minor changes in the proportion, Ki67-positivity or IFN-γ-positivity were observed in CD4 **(D)**, CD8 **(E)**, or Treg **(F)** lymphocytes isolated from the co-cultures. Experiment was performed, at least, in triplicate (n=3-4). Data represent mean ± SEM. One-Way ANOVA with Tukey´s post hoc test for multiple comparisons. **(G)** However, considerable immunosuppressive re-wiring was observed at the gene-expression pathway-levels. Positive NES scores represent the pathways enriched in lymphocytes isolated from the co-cultures with SPP1-primed RAW246.7 cells; negative NES scores represent the pathways enriched in lymphocytes isolated from the co-cultures with vehicle-primed RAWs.

Supplemental Figure 19

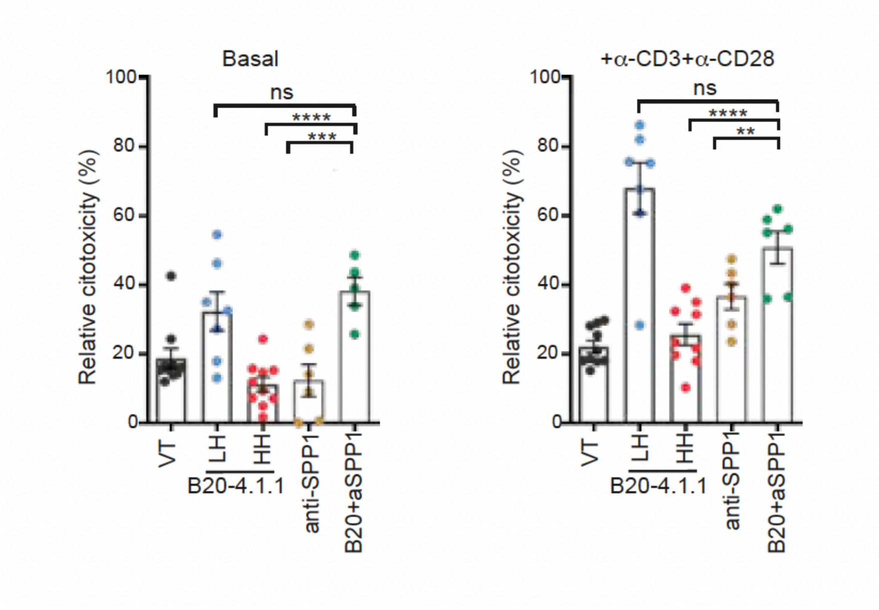

Supplementary Figure 19: recovery of the cytotoxicity assay by SPP1 depletion. In vitro cytotoxic assay (basal and CD3/CD28-stimulated) with leukocytes isolated from tumors treated with B20-4.1.1 alone (LH n=7/HH n=9) or with SPP1 depletion (n=6). One-Way ANOVA with Tukey´s post hoc test for multiple comparisons. Data are represented as means ± SEM. **: P<0.01; ***: P<0.001; ****: P<0.0001**;** ns: non-significant.

Supplemental Figure 20

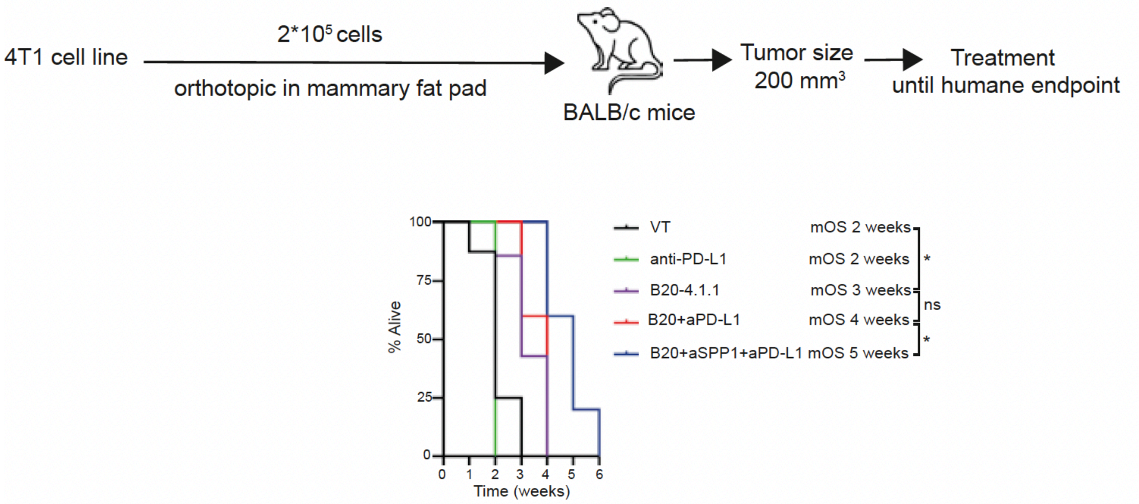

Supplemental Figure 20. Response of the triple-negative breast cancer model 4T1 to the combination of SPP1 depletion with antiangiogenic and anti-PD-L1 treatment. 4T1 cells were syngenically grafted in BALB/c mice and treatment was started when tumors reached an average size of 200 mm3. The triple combination reached a statistically significant effect in overall survival in this highly aggressive tumor model (n=3-8). *: *P*<0.05. ns: non-significant.

Supplemental Figure 21

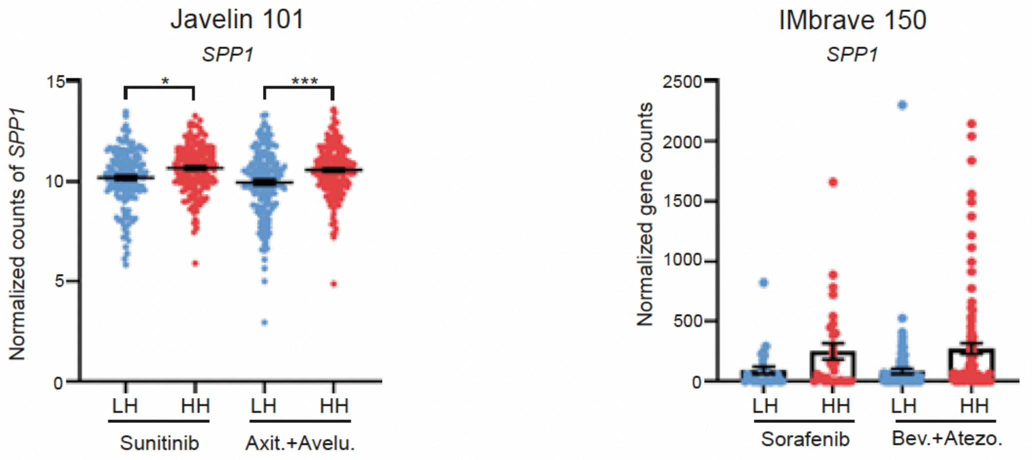

Supplemental Figure 21. Elevated levels of SPP1 in hypoxic tumors from the Javelin 101 and IMbrave 150 trial. Left: Total levels of *SPP1* measured by RNAseq in the baseline tumor samples from the sunitinib or axitinib+avelumab arms, in patients with low or high hypoxia according to their hypoxia signature (Javelin 101 trial) (n=157-187). Right: Same comparison, run in patients from the IMbrave 150 trial (n=26-107). Data represent mean ± SEM. Unpaired t test. *: *P*<0.05; ***: *P*<0.001.

Supplemental Figure 22

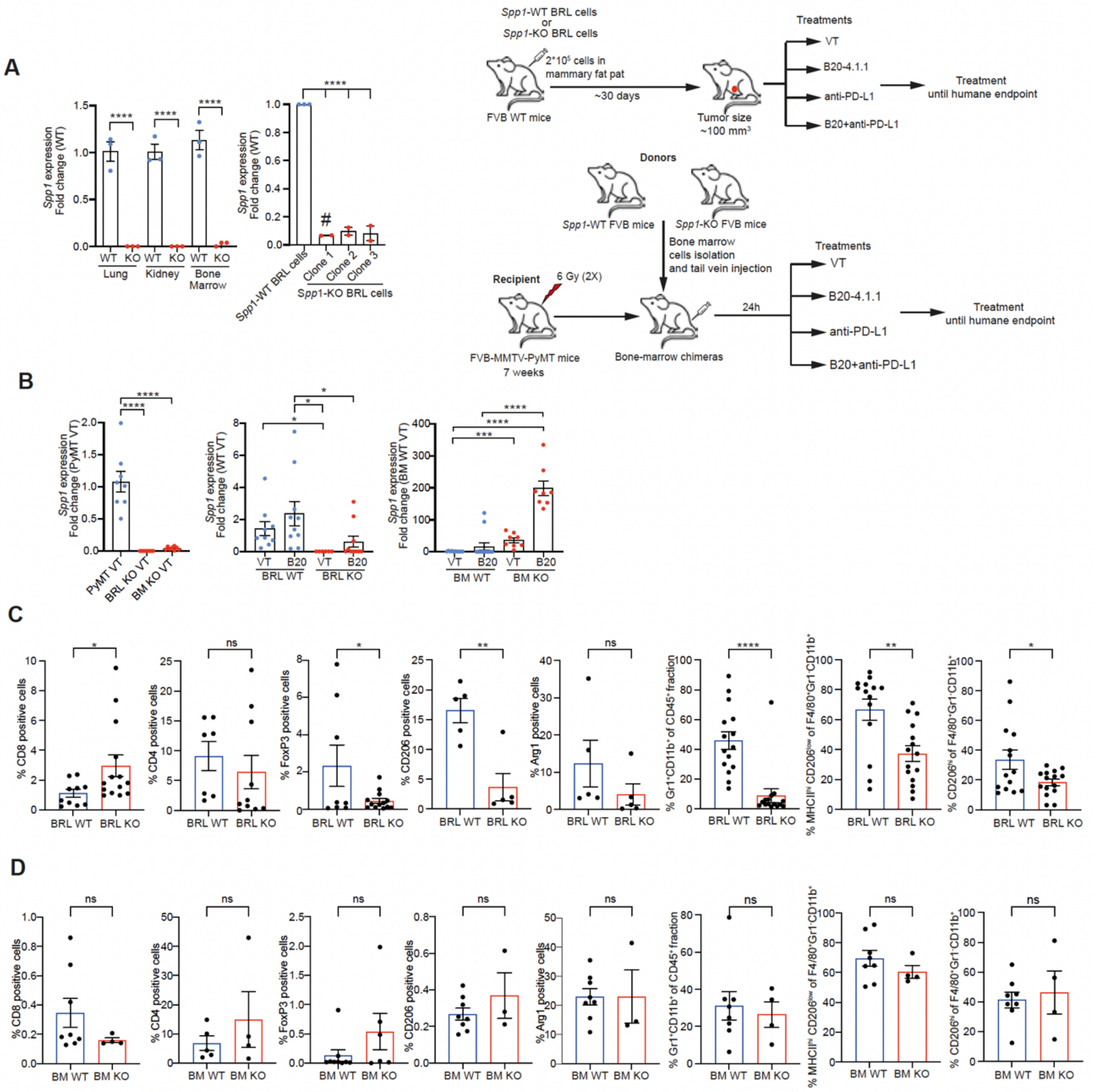

Supplemental Figure 22: Predominantly tumor epithelial origin of SPP1. (A) Cartoon depicting the generation of the bone marrow (BM) chimeras, in order to ascertain the unique contribution of myeloid SPP1, and tumor epithelial selective *Spp1* knockout (KO), to understand the contribution of the epithelial SPP1 pool. *Spp1* mRNA levels in different tissues of the *Spp1* KO animal (n=3) and *Spp1* mRNA levels in several BRL1468 clones are shown as well after CRISPR genetic deletion, compared to BRL1468 primary cell line (# symbol indicates clone selected for tumor engraftment). Unpaired t test. **(B)** Validation of compartment-restricted SPP1 depletion models and quantification of *Spp1* expression in tumors from the indicated genetic settings. **(C) and (D)** Quantification of immunohistochemistry showing the amount of tumor-infiltrating CD8, CD4 and FOXP3-positive lymphocytes and CD206- and Arg1-positive macrophages comparing VT from wild-type and tumor-epithelial *Spp1*-deleted grafts (n=5-15) **(C)** and tumors from wild-type or *Spp1* BM KO animals (n=4-10) **(D)**. The percentage of myeloid-derived suppressor cells (GR1+/CD11b+) and M1/M2 macrophages determined by flow cytometry from the CD45-positive cell fraction in the same models are also shown. Data represent mean ± SEM. Unpaired t test. *: *P*<0.05; **: *P*<0.01; ***: *P*<0.001; ****: *P*<0.0001; ns: non-significant.

Supplemental Methods

Immunohistochemistry analysis

For routine immunohistochemistry analysis, tissues were fixed in 10% formalin solution (Sigma-Aldrich; #HT501128) and embedded in paraffin. Immunohistochemical staining was performed on 3- to 4-µm paraffin sections mounted in TOMO® slides and dried overnight. Immunohistochemistry was performed using an automated immunostaining platform (Discovery XT-ULTRA, Ven-tana-Roche, Bond Max, Leica). All steps were performed on this staining platform using validated reagents, including deparaffination, antigen retrieval, and antibody incubation and detection. First, antigen retrieval was performed with the appropriate pH buffer (CC1 Ventana, Roche), and endogenous peroxidase was blocked (3% peroxide hydrogen). Then, slides were incubated with the appropriate primary antibody as detailed bellow. After the primary antibody incubation, slides were incubated with the visualization systems (OmniMap anti-Rabbit, Ventana, Roche) conjugated with horseradish peroxidase. Immunohistochemical reaction was developed using 3, 30-diaminobenzidine tetrahydrochloride (DAB) (ChromoMap DAB, Ventana, Roche) and nuclei were counter-stained with Carazzi's hematoxylin. Finally, the slides were dehydrated, cleared and mounted with a permanent mounting medium for microscopic evaluation. The following antibodies were used for immunohistochemistry: Pimonidazole staining were detected with FITC conjugated antibody (clone 4.3.11.3) and the chromogenic anti-FITC-HRP secondary reagent following the manufacturer's instructions(Hypoxyprobe^{TM} kit, hpi), anti-CD4 (clone D7D2Z, Cell Signaling Technologies, #25229), anti-CD8a (clone OTO94A, from CNIO monoclonal antibodies unit, #AM-OTO94A), anti-FOXP3 (clone 221D, from CNIO monoclonal antibodies unit, #AM-221D), anti-Arginase-1 (clone D4E3M, Cell Signalling Technologies, #93668) and anti-Mannose Receptor (CD206) (Abcam, #ab64693).

First, slides were acquired and digitalized with a AxioScan Z1 system (Zeiss). Next, the Zen 3.1 Blue software (Zeiss) or QuPath(1) v0.5.1 software were used to automate image analysis by creating a custom script for each antibody, based on training images.

For the final quantification, hypoxia was measured as the area of positive staining normalized to the total tumor area. Rest of stains were quantified as percentage of positive cells / total number of cells within the total tumor area.

Cell lines and in vitro cell response to hypoxic atmosphere experiments

BRL1468 cell line was established previously by our group as a primary PyMT breast tumor cell line (11). Briefly, pooled single cell suspension coming from PyMT tumor was seeded in growth medium and passaged 20 times before testing tumorigenic properties and preservation of the main features of the original tumor. BRL1468, 4T1 (ATCC, #CRL-2539), RAW 264.7 (ATCC, #TIB-71) and a panel of human triple negative breast cancer cell lines (MDA-MB-231, HCC1143, BT549 and T47D (ATCC, #HTB-26, #CRL-2321, #HTB-122, #HTB-133 respectively)) were cultured in 10%FBS DMEM medium (Sigma-Aldrich, #D5796) and tested routinely for mycoplasma using the MycoalertTM Mycoplasma Detection Kit (Lonza, #LT-118-07).

For in vitro drug treatments: mouse IFN-γ (Prepotech, #315-05) at 100ng/mL, mouse Spp1 (Biotechne, #441-OP-050/CF) at 1 μg/mL, anti-VEGFA (clone B20-4.1.1; acquired with Material Agreement Transfer with Genentech) and control isotype IgG2a (clone C1.18.4, BioXcell, #BE0085) at 10μg/mL were used.

For in vitro hypoxia experiments, cells were seeded in 6-well plates. After overnight incubation, cells were treated with drugs and controls. After treatment, one set of duplicate plates was exposed to standard incubators with 21%O_2 and 5%CO_2 (normoxia), while the other was place in a Hypoxia Workstation (Baker Ruskinn) with 1%O_2, 5%CO_2 and 94%N_2 (hypoxia) for 24h. After 24h under normoxic/hypoxic conditions, cells were washed with PBS and used for subsequent experiments.

Flow cytometry staining, cell sorting and FACS analysis

For cell sorting and FACS analysis, cells were resuspended in FACS buffer (PBS, 1% Bovine Serum Albumin, 1% FBS, 0.01% Sodium Azide) and were stained as follows. First, Fc Receptors were blocked using Purified Rat Anti-Mouse CD16/CD32 (Mouse BD Fc Block, #553142; 1:100). Then, staining was performed using the following anti-mouse antibodies: CD45 (Clone 30-F1, BioLegend, #103129, 1:400); CD45 (Clone S18009F, BioLegend, #157215, 1:200); CD8 (Clone 53-6.7, TONBO biosciences #35-0081, 1:100); CD8 (Clone 53-6.7, Invitrogen #56-0081-82, 1:100); CD4 (Clone GK1.5, eBioscience #11-0041, 1:400); CD4 (Clone RM4-4, BioLegend #116015, 1:400); PD-1 (Clone RMP1-30, BioLegend, #109103, 1:200); CD11b (Clone M1/70, BioLegend #101206, 1:800); F4/80 (Clone BM8, Biolegend, #123143, 1:200); Gr1 (Clone RB6-8C5, eBioscience, #363-5931-82, 1:400); MHCII (Clone M5/114.15.2, BioLegend, #107643, 1:1600); PD-L1 (Clone 10F.9G2, BioLegend, #124311, 1:200); CD31 (Biolegend, #390, 1:200); EPCAM (Clone G8.8, Biolegend, #118201, 1:400); CD3 (Clone 145-2C11, BD Biosciences #124311, 1:200); CD3 (Clone 17A2, BD Biosciences #757248, 1:200). For intracellular staining, cells were fixed and permeabilized with Foxp3/Transcription Factor Staining Buffer Set (eBioscience, #00-8333-56) in permeabilization buffer with the following antibodies: FOXP3 (Clone MF-14, BioLegend, #126409, 1:400); FOXP3 (Clone FJK-16s, Invitrogen, #404-5773-82, 1:600); CD206 (Clone C068C2, BioLegend, #141703, 1:400); CD206 (Clone MR6F3, Invitrogen, # 12-2061-82, 1:1200); IFNγ (Clone XMG1.2, Biolegend, # 505807,1:200), Ki67 (Clone B56, BD Biosciences, # 564071, 1:200). Dead cells were detected with DAPI for sorting experiments. For Flow cytometry analysis Zombie Fixable Viability staining (BioLegend; #423117; 1:500) or LIVE/DEAD Fixable Aqua (Invitrogen, #L34957, 1:1000). Cell sorting was conducted in BD Influx or BD FACS Aria IIu sorters (BD Biosciences) by specialized technicians. Flow-cytometry data were collected on a LSR Fortessa or BD-FACS Canto (BC Bioscience) flow cytometers and finally analyzed with FlowJo™ Software (software application version 10.7.1, Becton, Dickinson and Company). Cells were gated based on their size and granularity (FSC-A versus SSC-A), followed by doublet and dead-cell exclusion. The

alive cells (Life/Dead Aqua-) were identified as: Tumor cells: $CD45^{-}EPCAM^{+}$, Immune cells: $CD45^{+}$, CD4 T cell: $CD45^{+}CD4^{+}FOXP3^{-}$, Treg: $CD45^{+}CD4^{+}FOXP4^{+}$, CD8: $CD45^{+}CD8^{+}$, $GR1^{+}$ myeloid cells: $CD45^{+}CD11b^{+}Gr1^{+}$, Macrophages: $CD45^{+}CD11b^{+}$Gr1-F4/80^{+}, M1 macrophages: $MHCII^{high}$ $CD206^{low}$ Macrophages, M2 macrophages: $CD206^{high}$ Macrophages.

RNA extraction and quantitative RT-PCR (qRT-PCR)

For bulk tumor RNA extraction, a -80ºC frozen tumor piece of about 50mm^3 was homogenized in RLT buffer from RNA extraction kit (Qiagen, #74104) using Precellys 24 Tissue Homogeneizer (Bertin technologies). After homogenization, tumor lysates were centrifuged at 12,000g for 10min at 4ºC and supernatants were used for RNA extraction using RNeasy extraction kit (Qiagen, #74104) following manufacture's indications.

For cell lines RNA extraction, cells were first washed with PBS, then RLT buffer was added directly to the cells. The resulting cell lysate was used for RNA extraction following manufacture's indications.

Total RNA from tumors or cells was quantified using Nanodrop (Thermo Scientific). Total RNA (1μg) was used for cDNA synthesis using iScript cDNA synthesis Kit (BIO-RAD, #1708891) following manufacture's indications. cDNA was then used for quantitative real-time qPCR with Fast SYBR™ Green Master Mix (Applied Biosystems, #1129726). PCR amplification was performed using the QuantStudio™ 6 Flex Real_time PCR System (Applied Biosystems, #4485691) under the following thermal cycler conditions: 20s at 95°C; 40 cycles: 3s at 95°C and 30 sec at 60°C; 15s at 95°C and 1 min at 60°C. Relative gene expression was calculated using the $2^{-\Delta CT}$ method, using *β-actin* or 18S as reference genes. Primer list:

Target	Forward primer (5'-3')	Reverse Primer (5'-3')
β-actin	GGCTCCTAGCACCATGAAGA	CCACCGATCCACACAGAGTA
18S	AGAGTCCCTGCCCTTTGTACACA	CGATCCGAGGGCCTCACTA

Cd80	CCCCAGAAGACCCTCCTGATAG	CCGAAGGTAAGGCTGTTGTTTG
Cd86	TCAGTCAGGATGGGAGTGGTA	AGGTAGGAATGGCTCTTGGAT
Ctla4	GCTTCCTAGATTACCCCTTCTGC	CGGGCATGGTTCTGGATCA
Ifng	TCAAGTGGCATAGATGTGAAGA	TGGCTCTGCAGGATTTTCATG
Cd274	TGCGGACTACAAGCGAATCA	GCTGGATCCACGGAAATTC
Tgfb1	AGACATTCGGGAAGCAGTGC	TCCCGTTGATTTCCACGTG
Tnfa	CTGTAGCCCACGTCGTAGC	TTGAGATCCATGCCCGCTG
IL10	AGGCGCTGTCATCGATTTC	TGGCCTTGTAGACACCTTG
CD40	CCCTGCGATGGTGTCTTTGC	TGGCTTGTCAGTCGGCTTCC
MHCI 2Dq	GATCACGCAGATCGCCAAGGACAAT	CGTGCAACCCCACGTCACAG CCGTACATCC
MHCI 2Lq	GTCCCGCAGGCACTCACACGATCCAG	CCGTCGTATGCGTACTGCTCGTACCC
MHCI 2Kq	ACGACACTGAGTTGGTGCGCTTCGA	ACTCTGCTCATTGTCCTTGGCGATCT
IL1b	TGGTGTGTGACGTTCCCATT	CAGCACGAGGCTTTTTTGT
iNOS	CCCCGCTACTACTCCATCAG	CCACTGACACTTCGCACAAA
IL6	GCTACCAAACTGGATATAA	CCAGGTAGCTATGGTACTC
OPNa	CATGAGGCTGCAGTTCTCCT	TAAAGCTTCTTCTCCCTTGA
OPNb	CATGAGGCTGCAGTTCTCCT	CTGTAAAGCTTCTCCTCTGA
OPNc	AACCAGCCAAGGACTAACTA	TAAAGCTTCTTCTCCCTTGA
OPN4	AACCAGCCAAGGACTAACTA	CTGTAAAGCTTCTCCTCTGA
OPN5	TGGTGGTGATCTAGTGGTG	CATGGTCGTAGTTAGTCCTG
OPN total	GGATGAATCTGACGAATCTCAC	GGATGAATCTGACGAATCTCAC
ITGB3	GTAATCGAGATGCCCCAGAG	GTAATCGAGATGCCCCAGAG

ITGB1	GGTCAGCAACGCATATCTGG	CATTCCTCCAGCCAATCAGC
ITGAV	CTTCTCGGTGGTCCTGGTAG	CAGTCCGTGTTGCTAATTGGT
ITGA9	CAGTCCGTGTTGCTAATTGGT	CATCATCAATGTCGCCCAGG
Spp1	AATCGTCCCTACAGTCGATG	TAGGGTCTAGGACTAGCTTG

Supplemental Table 1. Primer sequences used in qPCR

RNA-seq analysis of intratumoral CD45+ and CD45-CD31-EPCAM+ cells.

For RNA-seq analysis, tumor samples were processed as previously described. CD45+ cells (immune cells) and $CD45^{-}CD31^{-}PDGFRa^{-}EPCAM^{+}$ cells (tumor cells) were sorted using BD Influx directly into RLT Lysis buffer. Then, RNA was extracted using RNAeasy Mini Plus Kit (Qiagen, #74134) and quantified in LabChip GX Touch Nucleic Acid Analyzer (PerkinElmer). RNA quality was determined by Agilent's 2100 Bioanalyzer Lab-Chip technology. Only samples with RNA Integrity Number (RIN)>7 was used for sequencing. Sequencing libraries were prepared with the "QuantSeq 3′ mRNA-Seq Library Prep Kit" (Lexogen, #015). Directional cDNA libraries are initiated by reverse transcription with oligodT priming and eventually sequenced in single-read format in a HiSeq 2500 instrument (Illumina). Sequencing read alignment and quantification and differential gene expression analysis was performed in the Bluebee Genomics Platform, a cloud-based service provider (www.bluebee.com). Briefly, reads are first trimmed with bbduk from BBTools (Bushnell B., BBMap, https://sourceforge.net/projects/bbmap/) to remove adapter sequences and polyA tails. Trimmed reads are aligned to the GRCm38/mm10 genome assembly with STAR v 2.5. Read counting is performed with HTSeq and differential gene expression analysis, between groups, is done with DESeq2. Gene set enrichment analysis (GSEA) versus Molecular Signatures Database v7.5.1 collection was performed on a ranked list of DESeq2 data, where log2FC of genes showing greater than 1.2 absolute fold change was divided by their corresponding p

value. Volcano plots were done with VolcaNoseR (https://github.com/JoachimGoedhart/VolcaNoseR).

For the calculation of the 3-gene hypoxic signature score, raw counts were normalized to transcripts per million (TPM) using the rnanorm python library and the Mus musculus GRCm38 genome assembly annotation. The score was computed as the average normalized expression of the 3 genes: *Vega*, *Slc2a1* and *Ca9*.

Single-cell Analysis

The Bollito pipeline was used to perform the initial steps of the single-cell RNAseq data analysis (2). An initial cell-level quality control was conducted, with outlier cells being removed based on read counts, number of expressed genes, and the percentage of mitochondrial counts distributions. After this preliminary filtering, the samples were merged into a single object that would be analyzed with Seurat v4.3.0 (3). This was employed to exclude cells with low quality (those with fewer than 2,500 UMIs) or cells identified as potential multiplets (those with more than 27500 UMIs). Only cells expressing a minimum of 50 house-keeping genes from a curated list specific to quality control were retained for analysis (4). This resulted in a total of 17,651 cells. Continuing with Seurat's functions, we normalized the expression data to log transcripts per 10,000 cells (TP10K) using NormalizeData. Variable genes were identified with FindVariableFeatures, selecting 2,500 genes using the 'vst' method. The data was then scaled with ScaleData, followed by a PCA conducted with RunPCA. We utilized RunUMAP for the UMAP embedding, opting for 16 dimensions based on an elbow plot, while keeping the remaining parameters at their default settings. Cell annotation was mapped using SingleR v2.0.0 (5).

We conducted cell-cell communication analysis with CellChat v1.6.1 (6), excluding cell types with fewer than 10 cells and using otherwise default parameters. For the analysis of macrophage sub-clusters, we first isolated the macrophages from the experiment and applied the previously described Seurat's pipeline again, with some modifications. We

identified variable genes using FindVariableFeatures, selecting 2,000 genes. UMAP embedding was performed with FindNeighbors using 12 dimensions, and the clustering was achieved with FindClusters set to a resolution of 0.65. Following this unsupervised clustering, we validated the roles of the obtained clusters by analyzing their top 10 most-upregulated genes using FindAllMarkers with a min.pct and a log fold-change threshold of 0.25. For GSEA, we utilized the Molecular Signatures Database v7.5.1 collection(7). We applied it to a list of differentially expressed genes from DESeq2 data, ranking genes with an absolute fold change (FC) over 1.2 by the division of their log2FC by their corresponding *P*-value.

Raw counts for the calculation of the 3-gene hypoxia signature score was also performed with the Bollito pipeline. A hypoxia score was defined as the average normalized expression of three genes (*Vegfa*, *Slc2a1*, and *Ca9*), after normalization to 10,000 counts per cell and log-transformation using Seurat.

Scripts to reproduce the analysis and the plots are available on https://github.com/cnio-ccg/breast_cancer_singlecell_collab.

An online portal to host cell information such as cell annotation, hypoxia percentage and UMAP visualization was created using ShinyCell v2.1.0 (8) and is publicly available at https://sunshine.bioinformatics.cnio.es/groups/compgenomics/Mouse_BreastCancer_Hypoxia_Study_final/ .

Measurement of cytokines

Cytokines CCL5 and TNFα were quantified from supernatants of TAMs and splenocytes co-cultures by mouse anti-Virus response panel with V-Bottom plate Legendplex kit (Biolegend, #740622) following the manufacturer's instructions.

VEGFA ELISA measurement

VEGFA levels were determined by Mouse VEGFA ELISA Kit (Invitrogen, #EMVEGFACL) from tumor lysates following manufacturer's instructions. Briefly, small

tumor pieces were placed in Tissue Extraction Reagent I (Invitrogen, # FNN0071) with 1x Halt Protease & Inhibitors Cocktail (ThermoScientific, # 78441) and homogenized using the mechanical disruptor Precellys 24 Tissue Homogeneizer (Bertin technologies). Protein concentration was determined using the BCA Protein Assay kit (Thermo Scientific; #23227). Protein lysates were diluted to a concentration of 1μg/μL for ELISA assay.

Activation of CD44/integrin β3-linked STAT3 and NF-κB/p65 signaling axis

To evaluate the role of SPP1 on activation of CD44/integrin β3-linked STAT3 signaling axis, RAW 264.7 cell line was cultured in FBS-free DMEM medium supplemented with 0.2%BSA. Cells were stimulated with with recombinant SPP1 (5μg/ml; Bio-Techne, #441-OP-050/CF) and treated with inhibitors of CD44 (10μg/ml; anti-mouse/human CD44 IM7 antibody, MedChem, #HY-P99126) and integrin β3 (5mM; ITGB3-IN-1 inhibitor, MedChem, #HY-175636) for 5 minutes.

To assess the role of SPP1 in the activation of NF-κB/p65 signaling axis, RAW 264.7 cells were cultured under the same conditions and treated with recombinant SPP1 and the indicated inhibitors for 15 minutes. Following incubation, cell lysates or RNA were collected for Western blot and RT-PCR analyses.

Western blot

Cells were washed with PBS and harvested in cold RIPA Buffer (#R0278; Sigma) containing 1% protease and phosphatase inhibitor cocktail (Thermo Scientific; #78441). Cell lysates were incubated at 4°C for 15 min, sonicated for 5 min and clarified by centrifugation at 14 000xg at 4°C for 15 min. Protein concentration was estimated using the BCA Protein Assay kit (Thermo Scientific; #23227) following the manufacture's instruction. 20 μg of proteins per sample were loaded on 10% SDS-PAGE gel and transferred to nitrocellulose membranes for further processing. 5% BSA was used to

block the membrane for 45 min at room temperature, followed by overnight incubation at 4ºC with the primary antibodies. The following primary antibodies were used: STAT3 (clone D3Z2G, Cell Signaling; #12640) (1:1000), phospho-STAT3 (Tyr705) (clone D3A7, Cell Signaling; #9145) (1:1000), NF-kappaB p65 (clone D14E12, Cell Signaling; #8242) (1:1000), phosphor-NF-kappaB p65 (Ser536) (clone 93H1, Cell Signaling; #3033) (1:1000), HIF1α (Bethyl laboratories Inc; #A300-286A) (1:1000), β-Actin (clone AC-15, Sigma-Aldrich; #A1978;) (1:5000) and vinculin (Abcam; #ab91459) (1:5000). Membranes were incubated with appropriate peroxidase-conjugate secondary antibodies for 1h at RT. Bands were visualized by the enhanced chemiluminescence (ECL) method (Clarity Western ECL substrate, #170-5060; BIO-RAD).

Hif1α binding sites determination in human and mouse *Spp1* promoter

Putative binding sites for HIF1α in the promoter region of human and mouse *SPP1* were predicted by bioinformatics analysis using the Eukaryotic Promoter Database (EPD)(9, 10).

In vitro co-culture assay response to hypoxic atmosphere conditions

Splenocyte-derived cells from wild-type FVB mice were co-cultured with RAW 264.7 cells that were exposed to different experimental conditions: normoxia ±SPP1 and hypoxia ±SPP1 for 72h. First, Raw 264.7 cells were seeded in a 24-well plate (40,000 cells/well) and placed in a humidified 5% CO_2 incubator at 37 °C. After 24h, fresh media with/without SPP1 (Biotechne, #441-OP-050/CF) at 1 μg/mL were added to the corresponding wells, and cultures were maintained under normoxic (21%O_2 and 5%CO_2) or hypoxic (1%O_2, 5%CO_2 and 94%N_2) incubator conditions. After 72h, primary splenocytes were obtained from wild-type FVB mice. Briefly, WT mice were sacrificed in a CO_2 chamber, and their spleens were harvested in cold PBS immediately after sacrifice. Splenocytes were mechanically dissociated using a syringe plunger. The cell suspension was filtered thought a 40μM strainer and centrifuged at 300g for 5 min at

room temperature (RT). Red blood cells were lysed using RBC lysis buffer (Invitrogen, #00-4333-57). After centrifugation, 300× g for 5 min, the cell pellet was resuspended in DMEM supplemented with 10% fetal bovine serum for counting. 1.5x10^6 splenocytes per well were co-cultured with RAW 264.7 cells exposed to different conditions. Cultures were maintained at 37ºC in a 5% CO_2 atmosphere for 48 h.

After 48h, 1x Brefeldin A (BioLegend, #420601) was added. Splenocytes were collected for FACS analysis and RNAseq analysis. Experiments were independently replicated at least three times.

Generation of *Spp1* knockout BRL1468 cell line

Spp1 sgRNA Clustered Regularly Interspaced Short Palindromic Repeats (CRISPR)/Cas9 All-in One Lentivector set system was purchased from Applied Biological Materials (ABM# LA445369). Recombinant lentiviruses were generated in HEK 293T cells by co-transfection of sgRNA-encoding plasmids targeting mouse *Spp1* with pVSV-G and psPAX2 packaging plasmids (Addgene). BRL1468 cells were infected with the sgRNA-encoding lentivirus and stable clones were established by puromycin selection (1.5 µg/ml) (Sigma). Seven days after lentiviral infection, mRNA levels of *Spp1* were measured by qPCR.

For the generation of mice graft model, 2^10^5 *Spp1*-WT BRL or *Spp1*-KO BRL cells were injected in each mammary fat pad. Tumor growth was checked periodically until reached ~100 mm^3 of size, when mice were randomized for treatments until humane end point.

Generation of *Spp1* knockout mice

All CRISPR reagents, Alt-R™ CRISPR-Cas9 sgRNAs and Alt-R™ S.p. Cas9 Nuclease V3, were purchased from Integrated DNA Technologies Inc. (IDT). CRISPR gRNAs were designed using the CRISPOR web tool (crispor.tefor.net) to target sequences flanking exons 4-7 of mouse *Spp1*-201, chromosome 5, forward strand (www.ensembl.org). The sequence of the sgRNAs were: 5´sgRNA 5´-CTCAACTACAATAACAAGAT-3´ targeting

intron 3 and 3´sgRNA 5´-ATGTTATGGGTGCCTATCTG-3´ downstream of *Spp1* exon 7, respectively.

gRNAS (10 mM each) were incubated with Cas9 Nuclease (8 mM) to form the ribonucleoprotein (RNP) complex 15 min at room temperature in 10 mM TrisHCl pH8 buffer containing 0,1 mM EDTA. The RNP complex was diluted 1 to 4 in Opti-MEM medium before electroporation.

Zygotes were obtained from crosses between FVB/N males and females. Females (5-8 weeks of age) were previously superovulated by consecutive administration of 5 IU of PMSG (at 3 pm of day -3) and 5 IU of hCG (at 1 pm of day -1) and matings were set up immediately after hCG administration. At 8 pm of day 0, the formation of vaginal plugs was monitored and cumuli were collected from oviducts. Cumuli were disaggregated with a hyaluronidase (Sigma H4272) solution (10 mg/ml in M2 medium) and diluted 1:2 in M2 medium at the moment of treatment. Free zygotes were cultured in KSOM medium until electroporation. Electroporation was performed in a NEPA21 electroporator using the CUY501P1-1.5 electrode. 20-40 embryos were electroporated in 5 ml of Opti-MEM-CRISPR mix. Pulse conditions were: 40V, 2,5 ms length, 50 ms interval, 4 repetitions, decay rate 10%, polarity + (pouring pulse) and 5V, 50 ms length, 50ms interval, 5 repetitions, decay rate 40% and polarity +/- (transfer pulse). Next day, zygotes that had developed to 2-cell stage were transferred to pseudopregnant CD1 females following standard protocols (Advanced Protocols for Animal Transgenesis. Springer Protocols. 2011. Pease S and Saunders T.L. editors).

F0 pups were genotyped by PCR from ear clip DNA using primers flanking the deletion: forward 5' GACCAGCAGCATTGGCATAC 3' and reverse 5' CAGCCCCTCCCTAATCACTG 3' to amplify a band of 4,85 Kb pb in the WT and 0,37 Kb pb in the deleted allele.

Animals positive for exon 3-7 deletion were crossed with FVB/N WT mice. The F1 animals were genotyped using the same protocol and the PCR product sequenced to

confirm the borders of the deletion. F1 positive animals with the correct deletion were considered founders of the line.

Bone marrow transplantation

Recipient 7-week old female FVB-MMTV-PyMT mice were lethally irradiated with two 6Gy doses, with an interval of 4 hours using an x-ray irradiator (Gammacell 1000 elite (S/N 443)/ MDS Nordion Inc.). After the second irradiation, recipient mice were transplanted through tail vein injection with a suspension of *Spp1*$^{+/+}$ or *Spp1*$^{-/-}$ total BM donor cells ($1.5.10^6$ cells). BM cells were obtained from limb bones of donor mice after euthanasia. Briefly, tibias and femurs were excised from donor *Spp1* WT-FVB and germline *Spp1*-knockout-FVB mice. The bone marrow was collected by flushing the long bones with sterile saline using a 25-gauge needle and 10-ml syringe. Recipient mice with non-injected bone marrow cells were kept as irradiation control. The day after bone marrow transplantation, recipient mice were randomized for drug treatments until humane endpoint.

Supplementary References

1. Bankhead P, Loughrey MB, Fernandez JA, Dombrowski Y, McArt DG, Dunne PD, et al. QuPath: Open source software for digital pathology image analysis. *Sci Rep.* 2017;7(1):16878.
2. Garcia-Jimeno L, Fustero-Torre C, Jimenez-Santos MJ, Gomez-Lopez G, Di Domenico T, and Al-Shahrour F. bollito: a flexible pipeline for comprehensive single-cell RNA-seq analyses. *Bioinformatics.* 2022;38(4):1155-6.
3. Hao Y, Hao S, Andersen-Nissen E, Mauck WM, 3rd, Zheng S, Butler A, et al. Integrated analysis of multimodal single-cell data. *Cell.* 2021;184(13):3573-87 e29.

4. Tirosh I, Izar B, Prakadan SM, Wadsworth MH, 2nd, Treacy D, Trombetta JJ, et al. Dissecting the multicellular ecosystem of metastatic melanoma by single-cell RNA-seq. *Science.* 2016;352(6282):189-96.
5. Aran D, Looney AP, Liu L, Wu E, Fong V, Hsu A, et al. Reference-based analysis of lung single-cell sequencing reveals a transitional profibrotic macrophage. *Nat Immunol.* 2019;20(2):163-72.
6. Jin S, Guerrero-Juarez CF, Zhang L, Chang I, Ramos R, Kuan CH, et al. Inference and analysis of cell-cell communication using CellChat. *Nat Commun.* 2021;12(1):1088.
7. Subramanian A, Tamayo P, Mootha VK, Mukherjee S, Ebert BL, Gillette MA, et al. Gene set enrichment analysis: a knowledge-based approach for interpreting genome-wide expression profiles. *Proc Natl Acad Sci U S A.* 2005;102(43):15545-50.
8. Ouyang JF, Kamaraj US, Cao EY, and Rackham OJL. ShinyCell: simple and sharable visualization of single-cell gene expression data. *Bioinformatics.* 2021;37(19):3374-6.
9. Meylan P, Dreos R, Ambrosini G, Groux R, and Bucher P. EPD in 2020: enhanced data visualization and extension to ncRNA promoters. *Nucleic Acids Res.* 2020;48(D1):D65-D9.
10. Dreos R, Ambrosini G, Perier RC, and Bucher P. The Eukaryotic Promoter Database: expansion of EPDnew and new promoter analysis tools. *Nucleic Acids Res.* 2015;43(Database issue):D92-6.
