# Supplementary material for: Osteopontin mediates acquired resistance to hypoxia-inducing antiangiogenics and promotes anti–PD-L1 refractoriness in breast cancer models
Source: J Clin Invest. 2026 Jul 15;136(14):e174092. doi: 10.1172/JCI174092 (PMC13367972; doi:10.1172/JCI174092)
Supplement: Unedited blot and gel images [file jci-136-174092-s063.pdf]

Figure 6C

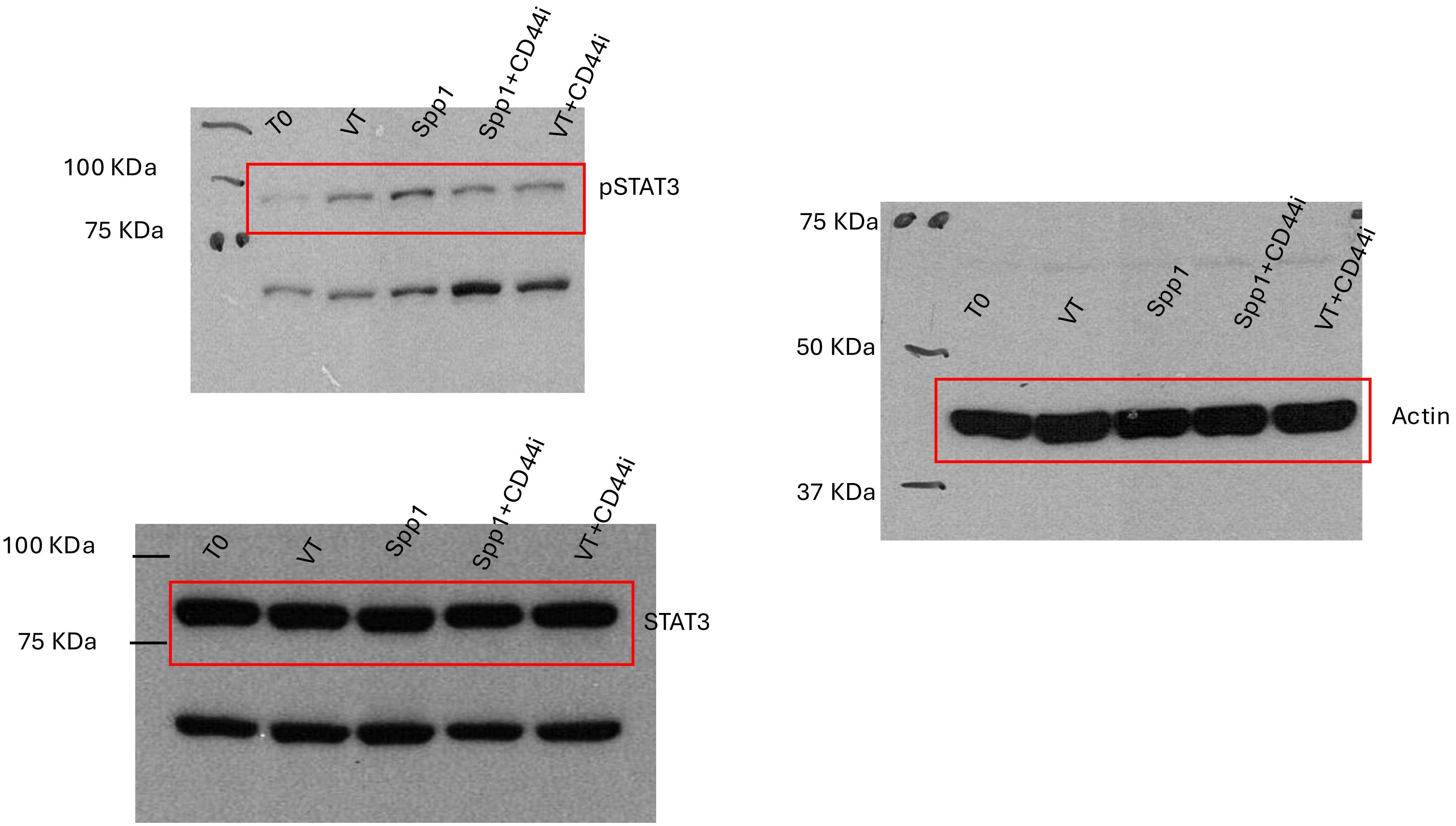
T0
VT
Spp1
Spp1+CD44i
VT+CD44i
100 KDa
75 KDa
pSTAT3
100 KDa
75 KDa
STAT3
75 KDa
50 KDa
37 KDa
Actin

Figure 6C

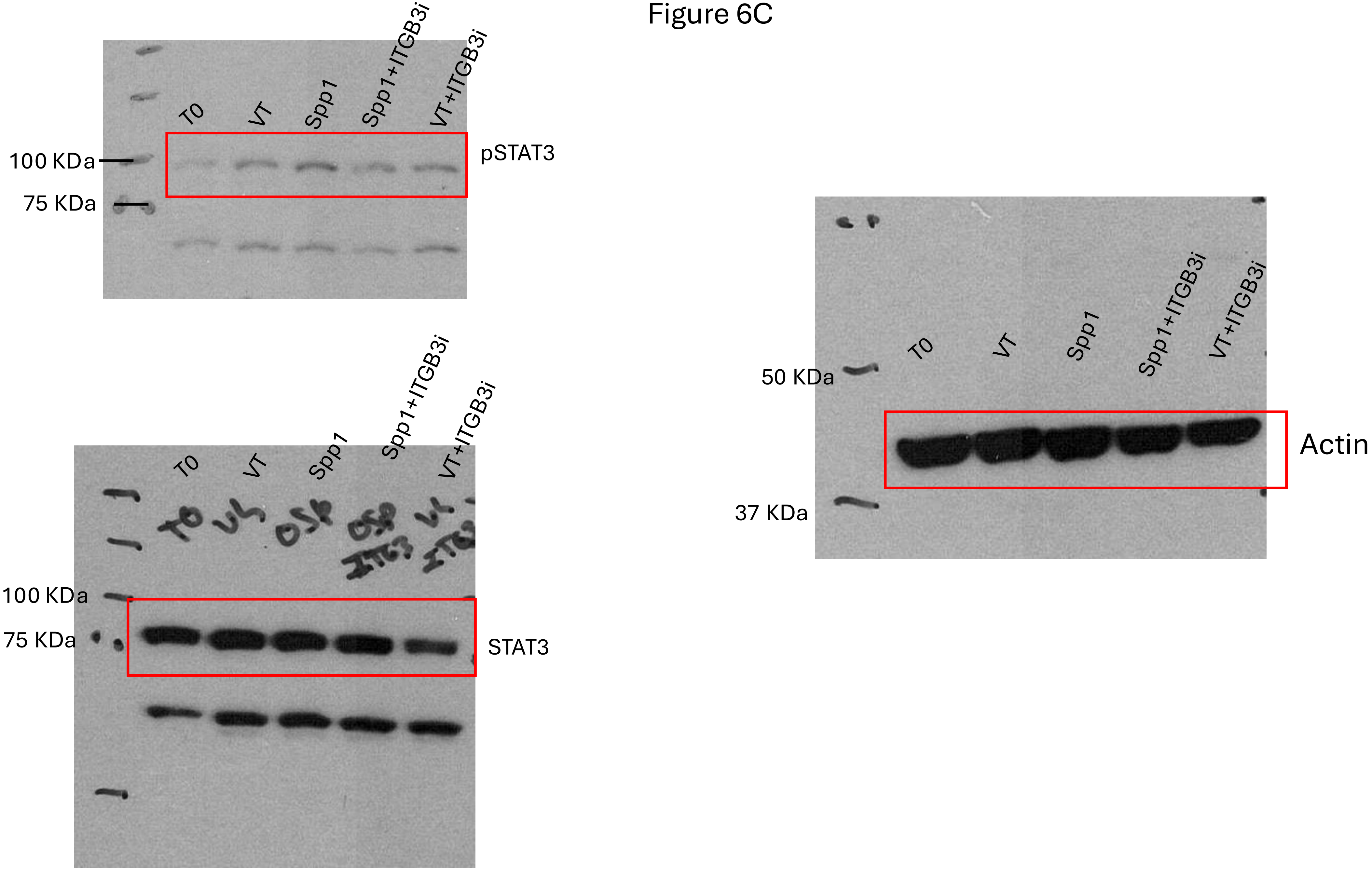
T0
VT
Spp1
Spp1+ITGB3i
VT+ITGB3i
100 KDa
75 KDa
pSTAT3
T0
VT
Spp1
Spp1+ITGB3i
VT+ITGB3i
100 KDa
75 KDa
STAT3
T0
VT
Spp1
Spp1+ITGB3i
VT+ITGB3i
50 KDa
37 KDa
Actin

Figure 6D

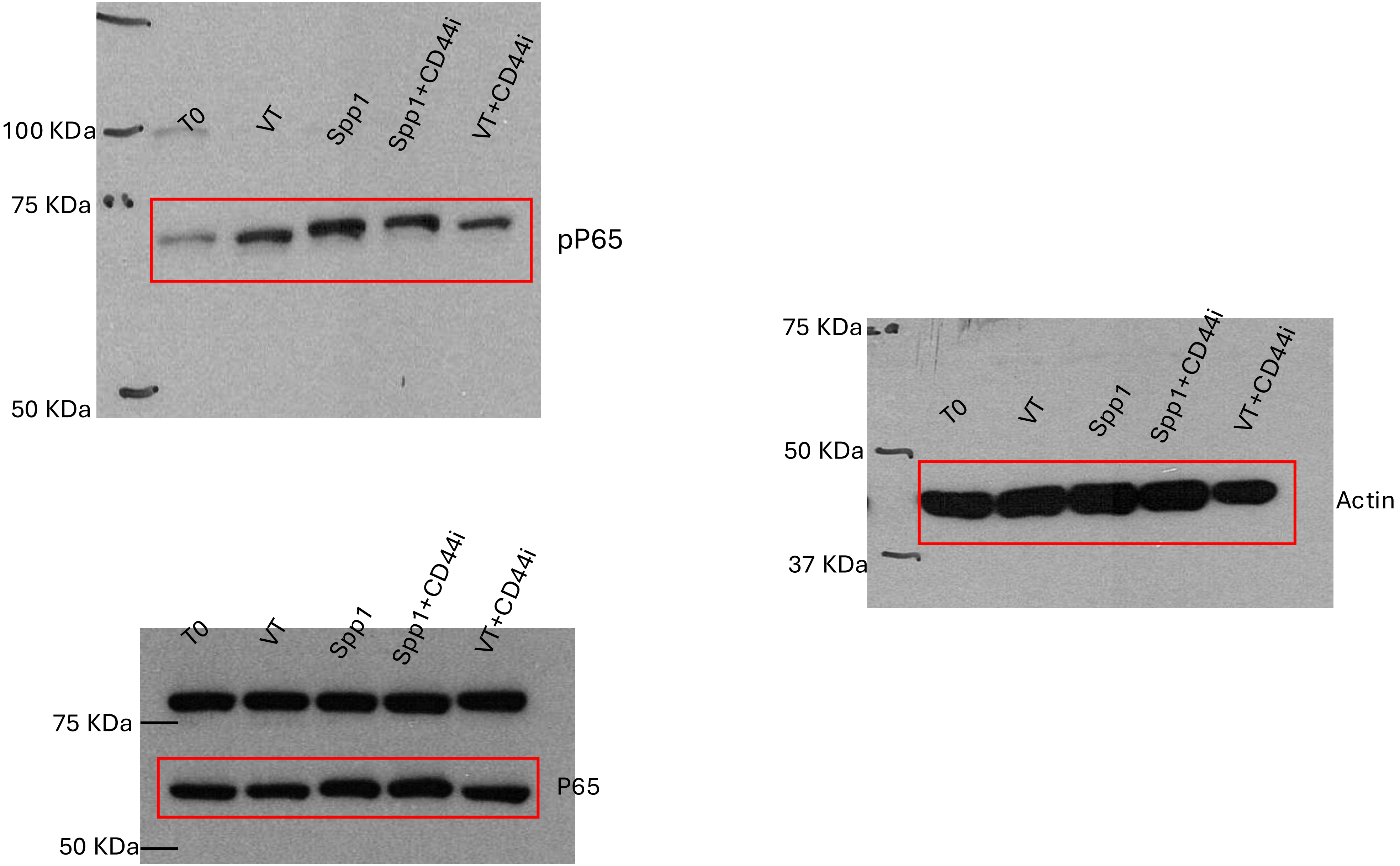
100 KDa
75 KDa
50 KDa
T0
VT
Spp1
Spp1+CD44i
VT+CD44i
pP65
75 KDa
50 KDa
T0
VT
Spp1
Spp1+CD44i
VT+CD44i
P65
75 KDa
50 KDa
37 KDa
T0
VT
Spp1
Spp1+CD44i
VT+CD44i
Actin

Figure 6D

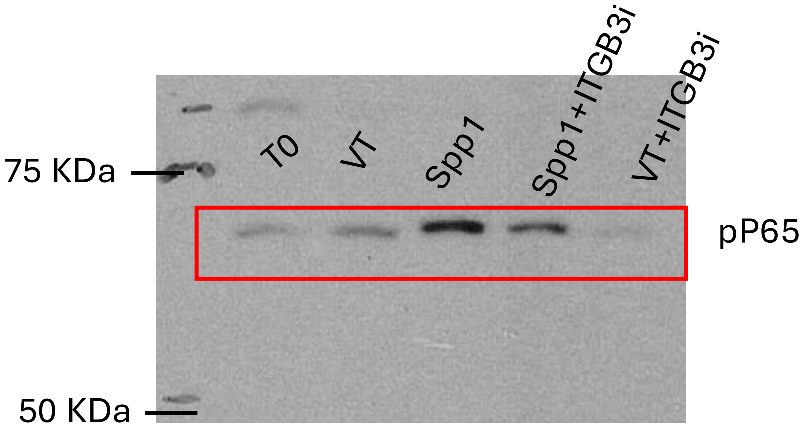

T0
VT
Spp1
Spp1+ITGB3i
VT+ITGB3i
75 KDa
50 KDa
pP65

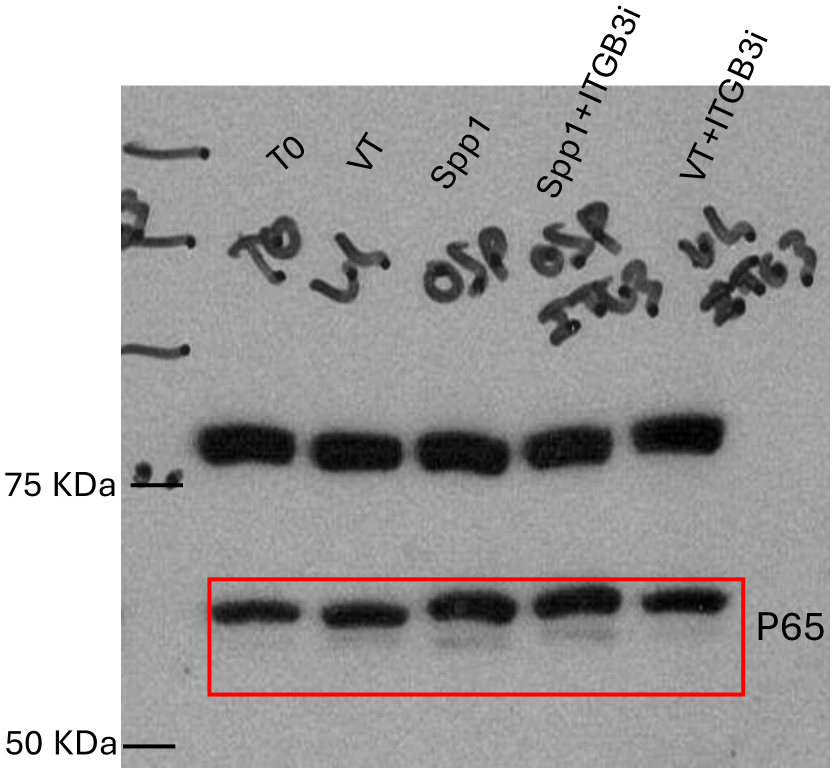

T0
VT
Spp1
Spp1+ITGB3i
VT+ITGB3i
75 KDa
50 KDa
P65

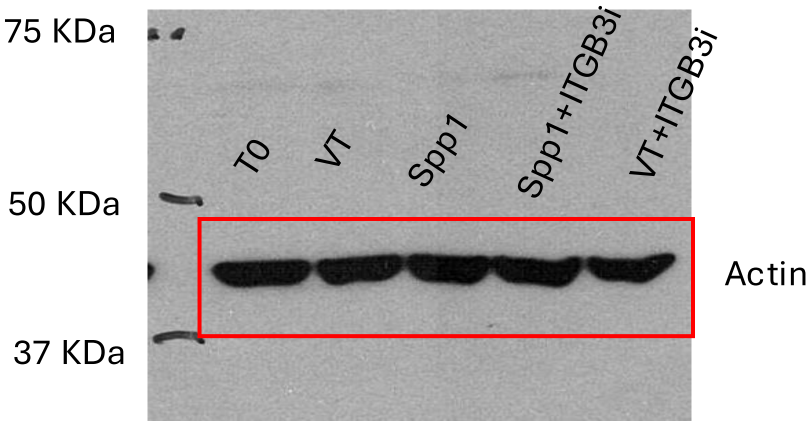

T0
VT
Spp1
Spp1+ITGB3i
VT+ITGB3i
75 KDa
50 KDa
37 KDa
Actin

Unedited blots

Supplemental Figure 13A

MBA-MB-231

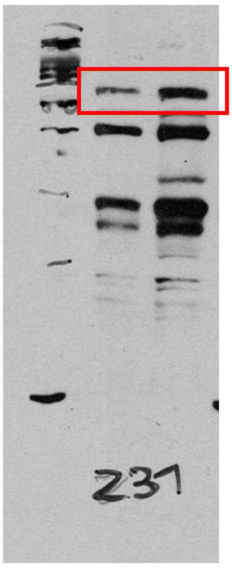

HIF1a

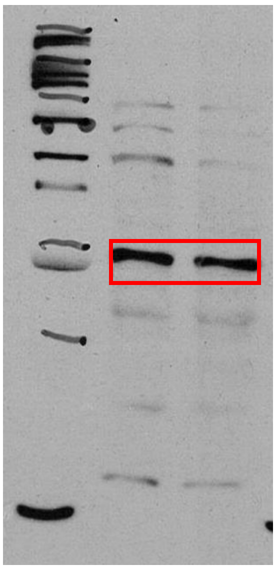

Tubulin

HCC1143

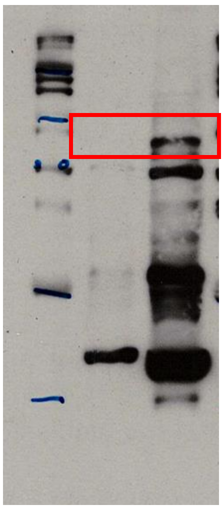

HIF1a

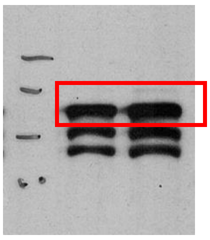

Vinculin

BT549

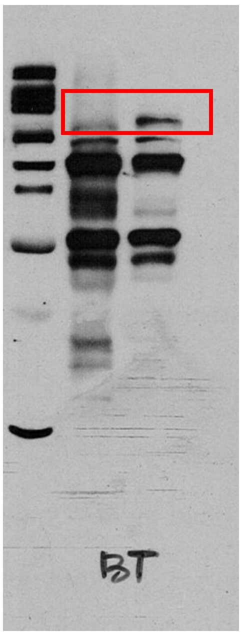

HIF1a

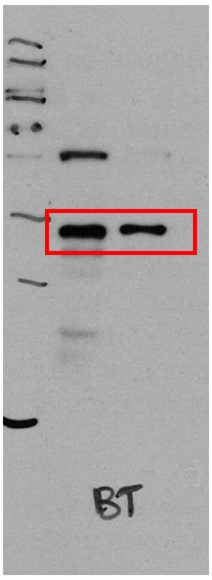

Tubulin

T47D

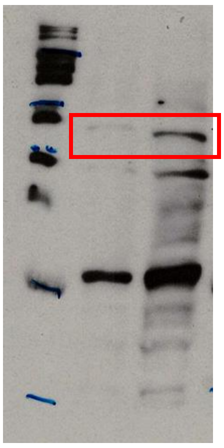

HIF1a

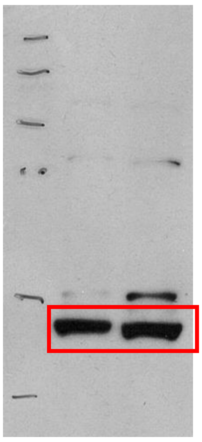

Tubulin

BRL1468

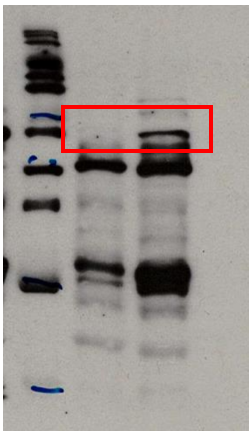

HIF1a

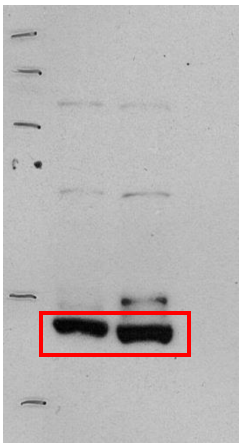

Tubulin
